# Validation of Smartphones in Arbitrary Positions Against Force Plate Standard for Balance Assessment

**DOI:** 10.3390/s25092639

**Published:** 2025-04-22

**Authors:** German Jack Ellsworth, Stephen M. Klisch, Britta Berg-Johansen, Eric Ocegueda

**Affiliations:** 1 Mechanical Engineering Department, College of Engineering, California Polytechnic State University, San Luis Obispo, CA 93407, USAsklisch@calpoly.edu (S.M.K.); 2 Biomedical Engineering Department, College of Engineering, California Polytechnic State University, San Luis Obispo, CA 93407, USA; bbergjoh@calpoly.edu

**Keywords:** balance assessment, center of mass, center of pressure, force plate, inverted pendulum, inertial measurement unit (IMU), smartphone, wearable sensors, rigid body kinematics

## Abstract

Balance assessment is a key metric for tracking the health and fall risk of individuals with balance impairment. Leveraging wearable sensors and mobile devices can increase clinical accessibility to objective balance metrics. Previous work has been conducted validating center of mass (COM) acceleration metrics from mobile devices against the gold standard force plate center of pressure (COP) position; however, most studies have been restricted to devices being placed close to the subject’s COM. In this study, rigid body kinematics and the inverted pendulum model were used to develop a novel methodology for calculating COM acceleration using mobile devices in arbitrary positions, as well as an approach for conversion of COM measurements to COP position for direct validation with force plate measurements. Validation of this methodology included a direct comparison of smartphone and force plate results for COM accelerations and COP positions, as well as statistical comparisons using Spearman’s rank correlation. The results show strong analysis performance for both approaches during a subject’s intentional swaying, with more limited results in cases of little motion. The strong performance warrants future work to further improve accessibility by removing dependence on motion capture systems or replacing them with cost-effective alternatives. The accurate tracking of COM acceleration and COP position information for mobile devices at arbitrary positions increases the flexibility for future mobile or at-home balance assessments.

## 1. Introduction

Assessment of human balance provides useful metrics to track the health and development of individuals. For example, poor balance increases the risk of falls, which are historically among the most prevalent sources of accidental injuries in all ages, and especially among the elderly [1,2]. In fact, impaired postural stability is a consistent predictor of fall risk [3,4]. Fall risk is further magnified by conditions that compromise motor functions in elderly people, such as Parkinson’s disease [5,6,7], dementia [8], and stroke [9]. In children, the development of postural stability is linked to physical activities that repeatedly use fundamental motor skills [10]. In fact, researchers have observed that strengthening motor skills has led to improved balance in children and adolescents with intellectual disabilities that impair motor function [11]. Thus, clinical balance assessments remain an important tool for objectively qualifying a patient’s health before determining fall prevention and motor rehabilitation strategies.

Clinical balance assessments typically rely on simple subjective tests that require little equipment or analysis. To assess fall risk, doctors often rely on patients recounting their fall histories to see trends; however, this metric can suffer from poor memory or recency bias [4]. For balance assessment, the Berg Balance Scale is commonly used and consists of a patient performing pre-determined tasks while a clinician assigns a score based on perceived ease of completion; these scores then determine the patient’s fall risk [12]. This scoring is often subjective since it requires visual evaluations and suffers from a ceiling effect (e.g., participants receiving the maximum score) due to the ease of the tasks [13,14]. The ceiling effect limits the effectiveness of such tests for patients with mild balance impairment, where the test may miss crucial improvements in balance [14]. Thus, there is a need for an objective and repeatable balance assessment with higher sensitivity to replace these subjective tests.

Since the 1950s, static and dynamic posturography—the quantitative evaluation of postural sway using force measurements—has been the prominent method for objective balance assessments [15]. Within static posturography, center of pressure (COP) displacement is the most frequently used recording due to its ease of measurement using the multiple load cells in force plates, and has thus become the “gold standard” in balance research and clinical studies [2,3,15,16]. The repeatability and objectivity of force plates are ideal for clinical testing; however, their use in practice is limited by the training, cost of equipment, and expertise required. Recognizing the inaccessibility of using force plates to measure the COP displacement, several researchers have shifted to alternatives, such as using mobile devices, to instead analyze the subject’s center of mass (COM) acceleration [4,6,17,18,19,20,21]. Mobile devices (smartphones and smartwatches) are convenient for acceleration-based balance research due to their embedded inertial measurement units (IMUs, generally consisting of MEMS accelerometers and gyrometers), relatively low cost, and high abundance among clinicians, patients, and the population at large [22].

Recently, Hsieh et al. [3] conducted balance tests on healthy participants to collect linear acceleration measurements from a smartphone held against the chest and COP information from a force plate. Their study successfully showed a strong correlation between RMS acceleration values (from the smartphone) and COP velocity data (from the force plate) [3]. Additionally, the smartphone data was found to be able to distinguish between participants with “high” and “low” fall risk (as measured by the physiological profile assessment) [3]. However, these results were limited to statistical comparisons between the phone’s acceleration and COP velocity, with the phone held near the COM. More recently, Lattanzi et al. [4] developed a post-processing technique to estimate COM acceleration using accelerometer data from a smartphone placed at approximately the height of the subject’s COM. Separately, using the classical “inverted pendulum” model proposed by Duarte et al. [15,23], Lattanzi et al. [4] also estimated the subject’s COM acceleration from force plate measurements to validate smartphone COM data. Results from two participants undergoing several balance tests (two-leg stance with open eyes, two-leg stance with closed eyes, one-leg stance with open eyes, and one-leg stance with closed eyes) showed strong agreement (from direct and feature comparison) between COM measurements from the smartphone and force plate. However, the post-processing method required the careful placement of the phone using a belt close to the subject’s COM.

To address the limitations of mobile device placement, Anthony et al. [24] explored using an anatomical calibration process (involving a calibration maneuver and Principal Component Analysis (PCA)) to allow the phone to be held anywhere by the user. Balance tests (consisting of double-leg stance, tandem stance, and single-leg stance) were conducted on 22 participants with one smartphone handheld and another placed in a body strap near the participant’s COM. After calibration, the handheld phone’s RMS angular velocity was found to have a moderate-to-strong correlation (using Pearson or Spearman correlation) with the RMS angular velocity of a phone placed in the body strap. Additionally, the handheld phone’s acceleration data were successfully able to detect differences in pose types. Although the calibration technique allows the phone to be handheld, the requirement of the calibration maneuver (a forward flexion) poses accessibility concerns for those with muscular injuries or neurological diseases. Furthermore, the method can detect pose differences but does not offer a direct prediction of COM acceleration or COP measurements.

The results of these previous studies show promise for replacing force plates with more accessible mobile devices for balance assessment. However, these studies have either relied on the mobile device being fastened near the subject’s COM to closely align the body and device axes or used pose/fall risk detection instead of directly measuring COM or COP data. The alignment increases setup complexity and requires suitable harnesses for each subject, imposing barriers for widespread use among the population. Additionally, past studies have validated mobile devices by correlating acceleration measurements, with no studies directly computing COP measurements, which remain the “gold standard” for balance assessments. Hence, this work aims to build on previous studies to (1) develop a methodology for the use of smartphones at arbitrary positions and orientations to track postural sway and (2) propose analysis techniques to transform smartphone measurements into COP displacement for direct comparison with force plates. For the first goal, kinematic data from two smartphones (one handheld and one in a harness) are used in conjunction with relative position tracking (achieved via motion capture cameras) to calculate the subject’s COM acceleration. These results were validated against COM accelerations obtained by applying the inverted pendulum model to force plate COP data. For the second goal, by further manipulating the inverted pendulum model in the frequency domain, the COM acceleration data of mobile devices were converted to COP displacement. This conversion allowed for backward compatibility of smartphone data with prior COP metrics and direct comparison of smartphone results to force plate COP data.

## 2. Materials and Methods

### 2.1. Trial Overview

**Figure 1:**
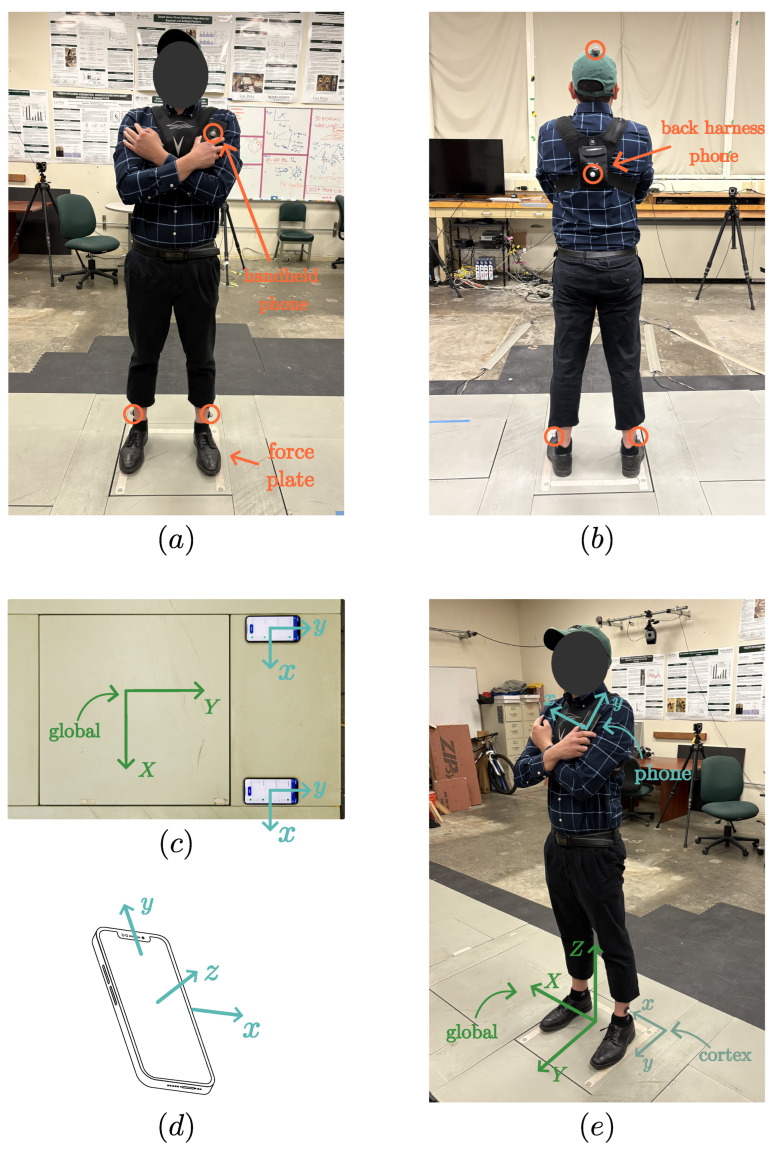
(**a**,**b**) Experimental setup showing phone locations, markers (circled), and force plate. (**c**) Initial phone placement aligned with desired global axis system. (**d**) Phone’s local coordinate system. (**e**) Example global coordinate system (XYZ) and device local coordinate systems (xyz).

Balance tests (eyes open, both feet stationary on a force plate in a natural shoulder width apart stance) were performed on one healthy young adult male subject with a mass and height of 101 kg and 187 cm, respectively. Ground reaction force (GRF) and center of pressure (COP) data were collected with a force plate (AccuGait, AMTI, Watertown, MA, USA), linear and angular kinematic data were collected by embedded IMUs in two smartphones (Apple iPhone X, Cupertino, CA, USA), and five marker position data were recorded using seven motion capture cameras (Motion Analysis, Santa Rosa, CA, USA). The experimental setup is depicted in Figure 1. Smartphones were placed in the subject’s hand and a harness on the subject’s back, and markers were placed on the subject’s ankles, the top of the subject’s head, the back of the handheld smartphone, and the pouch of the torso harness containing the second smartphone. The five marker locations were chosen to track the relative position between each phone and the subject’s center of mass, detailed in Section 2.3. Both smartphones were set on a stable surface in alignment with the subject’s anterior direction and the force plate’s global coordinate system, as shown in Figure 1c, before starting data collection and moving the devices to their respective positions for the trial.

Experiments lasted approximately 45 s and consisted of two data synchronization maneuvers (swaying and a sharp squat), approximately 30 s of motion for analysis, and a final synchronization maneuver for alignment verification. Motion was constrained to keep both feet in full natural contact with the force plate to ensure the validity of the inverted pendulum model used during analysis. Different motions were performed for each trial, as listed in Table 1. For each trial, intentional motion was engaged in pre-selected anatomical direction(s), referred to in this paper as the trial’s “engaged direction(s)”. Lateral anatomical directions that do not correspond to a trial’s deliberate swaying direction are referred to as “unengaged”. The term “unengaged” does not signify that no motion is expected; conversely, some swaying is anticipated. The terms “engaged” versus “unengaged” are instead used to distinguish between deliberate versus indeliberate swaying.

### 2.2. Data Acquisition

Data acquisition (DAQ) for each trial involved two smartphones, one force plate, and a 3D marker tracking system. Collection types and considerations for each device type are described below. To ease the acquisition procedure, the start times, stop times, and data acquisition frequencies were not synchronized between devices. After retrieval, the data were up-sampled to match the highest acquisition frequency before trimming to a synchronized time span. Additionally, each device collects data in its local coordinate system, so a coordinate transformation was applied to each dataset to match the body’s anatomical directions, as shown in Figure 1e.

Force plate data were taken with a Cortex processing environment (Version 6.2.13.1751, Motion Analysis, Rohnert Park, CA, USA) at a frequency of 150 Hz. Data included the resultant force vector and COP position on the surface of the plate with respect to Cortex’s coordinate system, shown in Figure 1e.

Three-dimensional marker position data were taken using the motion capture cameras connected to the Cortex processing environment at a frequency of 150 Hz. Positions were reported as vectors with respect to Cortex’s coordinate, shown in Figure 1e. In cases where a marker was not detected for one or more frames, gaps in position data were filled using a native “cubic fit” tool (though these gaps appeared infrequently and for no more than one-tenth of a second at a time).

Smartphone IMU data were taken using the MATLAB Mobile app (Version 9.8, MathWorks, Natick, MA, USA) on both smartphones at the maximum frequency of 100 Hz. Linear acceleration, angular orientation, and angular velocity were recorded for each test. MATLAB reports linear acceleration and angular velocity in the phone’s local coordinate directions (see Figure 1d) and orientation using Euler angles of azimuth, pitch, and roll [25]. The linear acceleration and angular velocity recordings contained high-frequency noise when the device was rested on a stationary surface, indicating the need for a low-pass filter. Implementation of this filter is described in Section 2.5.

### 2.3. Analysis

#### 2.3.1. Rigid Body Kinematics

Smartphone linear accelerations (${\vec{a}}_{P}$) were used to predict the subject’s COM accelerations (${\vec{a}}_{G}$) using a standard rigid body relative motion equation:

(1) $${\vec{a}}_{G}={\vec{a}}_{P}+\vec{\alpha }\times {\vec{r}}_{G/P}+\vec{\omega }\times \left(\vec{\omega }\times {\vec{r}}_{G/P}\right)$$

where $\vec{\alpha }$ is the angular acceleration of the participant’s body, ${\vec{r}}_{G/P}$ is the position of the COM with respect to the position of the smartphone, $\vec{\omega }$ is the angular velocity of the participant’s body, $a_{G}$ is the linear acceleration at the participant’s COM, and $a_{P}$ is the linear acceleration of the phone. The angular velocity and linear acceleration of the phone were directly reported by the smartphone, and the angular acceleration was approximated by differentiating the angular velocity data via finite difference, as described in Section 2.4.

**Figure 2:**
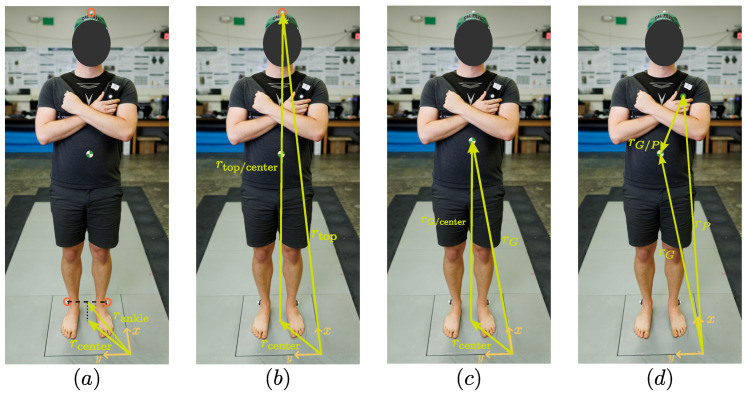
Steps for the determination of COM location. (**a**) Projection of ankle pivot onto force plate. (**b**,**c**) Determination of COM location. (**d**) COM position relative to handheld smartphone.

Lastly, the relative position vector, ${\vec{r}}_{G/P}$, was estimated using the markers and camera system. COM position during the crossed-arm position (as shown in Figure 1) was taken to be at 57% of the subject’s height, which is the average value between the arms down and arms raised stances analyzed by NASA [26]. This is consistent with other values used in inverted pendulum applications [15,27]. Figure 2 shows the process used to find the COM position with respect to the handheld phone, and the same procedure was employed for the back harness phone using its own marker position. First, averaging the ankle marker positions gives the position of the pivot in the inverted pendulum model, $r_{\mathrm{ankle}}$, and projecting this vector onto the force plate gives the lateral position of the COP and COM during perfect balance, $r_{\mathrm{center}}$. A vector can then be formed from this projected point to a marker at the top of the subject’s head, $r_{\mathrm{top}/\mathrm{center}}$. By assuming the COM lies on the line between the center and the marker on the subject’s head, $r_{\mathrm{top}/\mathrm{center}}$ can be scaled by 57% to obtain $r_{G/\mathrm{center}}$, which after adding $r_{\mathrm{center}}$ gives the vector position of the COM, $r_{G}$. Finally, the difference between this COM position and the position of the respective smartphone marker was used as ${\vec{r}}_{G/P}$ in Equation (1).

#### 2.3.2. Inverted Pendulum Model: Force Plate COP Displacement to COM Acceleration

The inverted pendulum model has been well-established in the literature and used for estimating COM acceleration from force plate data [15,23,28]. A full derivation and corresponding free body diagrams are included in Appendix A. In summary, the inverted pendulum model presupposes that the bottom face of the foot makes full contact with the supporting surface (i.e., the force plate), the point equidistant from both ankles acts as a fixed point of rotation for the body above them, the body from the ankles upward acts as a rigid body, and the body undergoes only small rotations about the pivot [15]. Additionally, it is assumed that the participant’s stance is two-legged with feet side by side. A consideration for stance width was performed [28], but its inclusion is beyond the scope of this analysis. A small angle approximation constrains the COM acceleration to a lateral plane (i.e., vertical accelerations are assumed to be zero). Evaluation of this model in the antero–posterior (AP) and medio–lateral (ML) directions (${\hat{e}}_{y}$ and ${\hat{e}}_{x}$ respectively) each results in equations of motion describing the subject’s COM in that direction:

(2) $$\frac{d^{2}}{dt^{2}}\left({\vec{r}}_{G}\cdot \hat{e}\right)=\frac{mgd}{I_{p}}\left({\vec{r}}_{G}-{\vec{r}}_{COP}\right)\cdot \hat{e}$$

where ${\vec{r}}_{COP}$ is the displacement of the COP, ${\vec{r}}_{G}$ is the displacement of the COM, $\hat{e}$ is either ${\hat{e}}_{y}$ for the antero–posterior direction motion or ${\hat{e}}_{x}$ for the medio–lateral direction, *g* is the magnitude of gravity, *d* is the distance between the ankle and the COM, *m* is the subject’s mass, and $I_{p}$ is the mass moment of inertia about the pivot point in the lateral perpendicular direction (e.g., the medio–lateral direction for the ODE describing antero–posterior motion). The mass moment of inertia about the COM was calculated following linear regression equations by Ledebt and Breniere [29]:

(3) $$I_{G}=kmH^{2}$$

where *H* is the height of the subject and *k* is the regression slope taking values 0.0572 for the antero–posterior direction and 0.0533 in the medio–lateral direction [29,30]. This can be translated to a mass moment inertia of the body about the ankle pivot point via parallel axis theorem [15,23,27]:

(4) $$I_{p}=I_{G}+m{\left(0.57H-0.039H\right)}^{2}$$

Conversion of Equation (2) to the frequency domain using the Fourier transformation results in a transfer function that facilitates conversion of COP position to COM position. For example, for motion in the sagittal plane,

(5) $$\frac{{\hat{r}}_{G,\;y}\left(\omega \right)}{{\hat{r}}_{COP,\;y}\left(\omega \right)}=\frac{{\omega_{0}}^{2}}{{\omega_{0}}^{2}+\omega^{2}}$$

where the $\;\hat{\cdot }\;$ symbol denotes the Fourier transformed versions of each position time series, ω is the angular frequency, and $\omega_{0}=\sqrt{mgd/I_{p}}$ is the natural frequency of the system [15]. Application of the fast Fourier transform (FFT) facilitates the conversion of force plate COP data to the frequency domain, where the transfer function and inverse FFT can be applied to find the COM position. Subsequent application of a second-order finite difference (see Section 2.4) generates COM acceleration components that can be compared with those found from smartphones, as described in Section 2.3.1.

#### 2.3.3. Inverted Pendulum Model: Smartphone COM Acceleration to COP Displacement

Although the approach of Section 2.3.2 has been well established, here a different approach that builds off the inverted pendulum model is proposed. The aim is to estimate the COP displacement by solving the equations of motion, which will allow the smartphone COM acceleration from Section 2.3.1 to be converted to COP displacement.

First, rearranging Equation (2) in terms of COP displacement (${\vec{r}}_{COP}$) and COM acceleration (${\vec{a}}_{G}$):

(6) $${\vec{r}}_{COP}=\int_{0}^{t}\int_{0}^{\tau }{\vec{a}}_{G}\left(s\right)\;ds\;d\tau -\frac{{\vec{a}}_{G}}{{w_{0}}^{2}},$$

where $\int_{0}^{t}\int_{0}^{\tau }{\vec{a}}_{G}\left(s\right)\;ds\;d\tau$ is the center of gravity displacement rewritten in terms of center of gravity acceleration. Conversion of the integral term to the frequency domain would yield additional terms that inform the zero frequency response, or mean tendency. Since balance tests are performed in a relatively stationary position, this mean value should be effectively zero. Additionally, any error incurred from this omission is removed through a high-pass filter on the COP results, as described in Section 2.5. Thus, the Fourier transform of an integral term would simplify to

(7) $$F\{\int_{-\infty }^{t}\int_{-\infty }^{\tau }f\left(s\right)\;ds\;d\tau \}=\left(-\frac{1}{\omega^{2}}\right)\hat{f}\left(\omega \right)$$

where F is the Fourier transform. Applying Equation (7) to the double integral in Equation (6) provides the transfer function:

(8) $$\frac{{\hat{r}}_{COP}\left(\omega \right)}{{\hat{a}}_{G}\left(\omega \right)}=-\left(\frac{\omega^{2}+{\omega_{0}}^{2}}{\omega^{2}\;{\omega_{0}}^{2}}\right)$$

where $\omega_{0}=\sqrt{mgd/I_{p}}$ is the natural frequency of the system [15]. This transfer function facilitates the conversion of smartphone COM acceleration data to COP displacement.

When integrated, any bias in the linear acceleration and angular velocity of the IMU data results in an undesirable drift in the output for cases like that shown in Equation (6). While the approach described in this section avoids integration drift, the static offset instead manifests itself as an undesirable low-frequency response when the FFT and inverse FFT are employed. This further warrants the use of a high-pass filter, as described in Section 2.5.

### 2.4. Finite Difference

In all cases where a derivative was needed (first derivative in Section 2.3.1 and second derivative in Section 2.3.2), a 7-point finite difference formula was selected [31,32]. Data points were selected to be as centered as possible, with calculations near the tail ends of the datasets being asymmetric (e.g., using the first seven data points in the set when calculating the derivative for the first four frames of data).

### 2.5. Filtering

Filtering bookended the analysis with a low-pass filter applied to the initial smartphone data reported by MATLAB and a high-pass filter applied to the COP displacement data computed from the smartphone measurements. Both the low-pass and high-pass filter were applied only to smartphone data with no filtering performed to the force plate data. A fourth-order, zero-phase Butterworth filter was applied in both instances, with a low-pass cutoff frequency of 2 Hz and a high-pass cutoff frequency of 0.12 Hz. This Butterworth filter is commonly used for biomechanics applications, and cutoff frequencies were determined by inspection of residual plots, as described in the work by Winter [23,33]. The chosen low-pass cutoff frequency was on the lower end compared to other studies wherein accelerometers were used for balance applications, where values of 3–6 Hz are common [6,34,35]. For data that are differentiated, such as the determination of angular acceleration from angular velocity, recommendation formulas report cutoffs as high as 9 Hz [23,36]. However, this model showed increased performance for lower cutoff frequencies, so a lower frequency was chosen. The limitations of this selection are discussed further in Section 4.4.

### 2.6. Error Propagation

Confidence intervals are estimated for the COM acceleration computed from the smartphone data (following Section 2.3.1) to aid in comparison with force plate COM data. Measurement and statistical uncertainties were considered before being propagated in the time domain using Equation (9)

(9) $$U_{f}\left(x_{1},x_{2},\ldots x_{i}\right)=\sqrt{\sum_{i=1}^{n}{\left(\frac{\partial f}{\partial x_{i}}\right)}^{2}{U_{x_{i}}}^{2}}$$

where *f* is a function of variables $x_{i}$, *n* is the number of variables containing uncertainties for consideration, and $U_{x_{i}}$ is their respective uncertainty. For uncertainty in COM acceleration, *f* follows Equation (1), with the following uncertainties used. Statistical uncertainties in the phone’s angular velocity and linear acceleration were computed from data collected while phones were left stationary. The uncertainty in the angular acceleration was found by applying Equation (9) to the finite difference method for each frame of data. The smartphone IMUs are not located precisely at their respective markers, so an uncertainty was used to account for this offset. The uncertainty in the height of the participant’s COM (the 57% scaling) was determined by propagating NASA’s reported COM model uncertainties [26]. Lastly, the smartphone’s angular position and cortex’s reported marker location were assumed to have negligible uncertainty.

Confidence intervals for any data computed from force plates (COM acceleration or COP displacement) were neglected since the force plate serves as the “gold standard”. Meanwhile, confidence intervals for the COP displacement determined from smartphone data (following Section 2.3.3) were not found since it would require propagating uncertainties through the forward and inverse Fourier transform, which is out of the scope of this work.

### 2.7. Results Correlation

The Spearman’s rank correlation coefficient and *p*-value were used as objective measures of the degree of agreement between the smartphone and the force plate results (for both COM acceleration and COP position data) [37]. This coefficient was computed for each component, vector magnitude, and smartphone, leading to eight correlation coefficients per model.

### 2.8. Analysis Summary

To summarize the approach: data are acquired and put through pre-processing to ensure compatibility and reduce noise. For the first analysis pathway, smartphone data and marker positions are used in the rigid body kinematics computation to determine the COM acceleration. Applying FFT to the force plate’s COP position data converts the signal to the frequency domain where the inverted pendulum transfer function is applied to form the COM position in the frequency domain, and an inverse FFT generates the COM position in the time domain where finally a finite difference method is applied twice. This facilitates the comparison of COM acceleration results from the smartphones and force plate.

**Figure 3:**
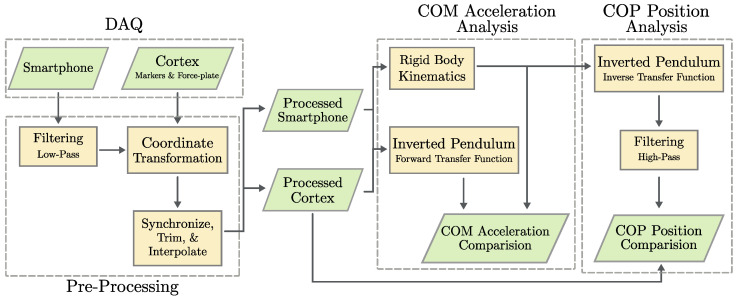
Methodology overview. Diagram shows a high level summary of all methodology steps and their connection to each other, including both proposed analysis pathways: COM acceleration and COP position.

For the second analysis pathway, the COM acceleration output from the previous rigid body kinematics analysis is considered. Applying FFT to smartphone COM acceleration components facilitates the conversion to the frequency domain and the transfer function is applied to form COP position in the frequency domain; then, an inverse FFT generates the COP result in the time domain. This facilitates the direct comparison of smartphone COP position components with force plate COP displacement data. Figure 3, shows these analysis steps.

The force plate is widely used as a validation standard for balance research [3,4,38]; hence, the performance of both models was based on their agreement with force plate results. This consistency is shown in the results in two main ways. First, the force plate’s COM acceleration curves should remain within the uncertainty window of the smartphone results they are plotted against. This verification is further illustrated by error plots that have been generated by taking the difference between the two curves in each COM acceleration component. Since validation consists of matching the force plate and smartphone data, an error plot showing a difference of 0 consistently captured by the uncertainty window indicates agreement between devices. Second, smartphone results were compared to force plate results by calculating the Spearman’s rank correlation coefficient and root mean square errors (RMSE) between the two datasets.

## 3. Results

Below we show results for trial 2, with the participant swaying in the ML direction. The remaining trials’ results (no swaying, swaying in the AP direction, and random swaying) are found in Supplementary Figures S1–S18, with similar trends as those observed in trial 2.

### 3.1. Center of Mass Acceleration Comparison

**Figure 4:**
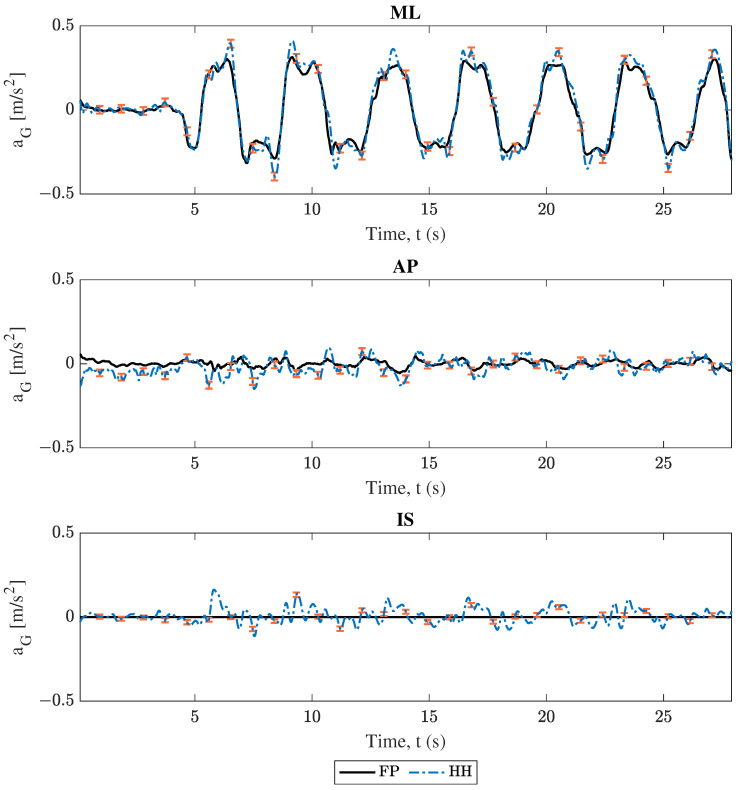
Trial 2 center of mass acceleration ($a_{G}$) comparison. Force plate (FP) versus handheld smartphone (HH), where ML, AP, and IS represent the medio–lateral, antero–posterior, and inferior–superior axes respectively. The error bars represent confidence intervals.

**Figure 5:**
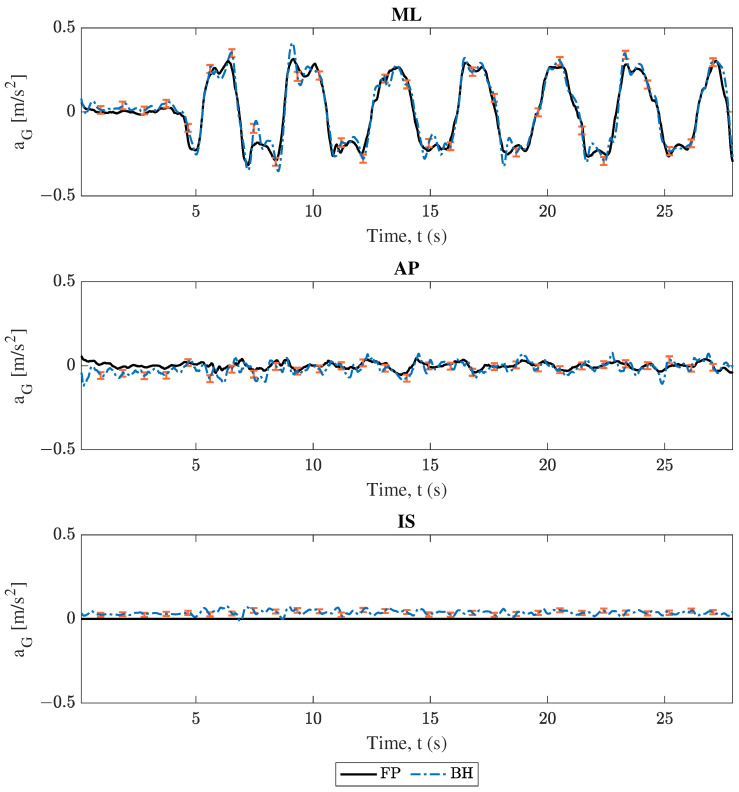
Trial 2 center of mass acceleration ($a_{G}$) comparison. Force plate (FP) versus back harness (BH) smartphone, where ML, AP, and IS represent the medio–lateral, antero–posterior, and inferior–superior axes respectively. The error bars represent confidence intervals.

**Figure 6:**
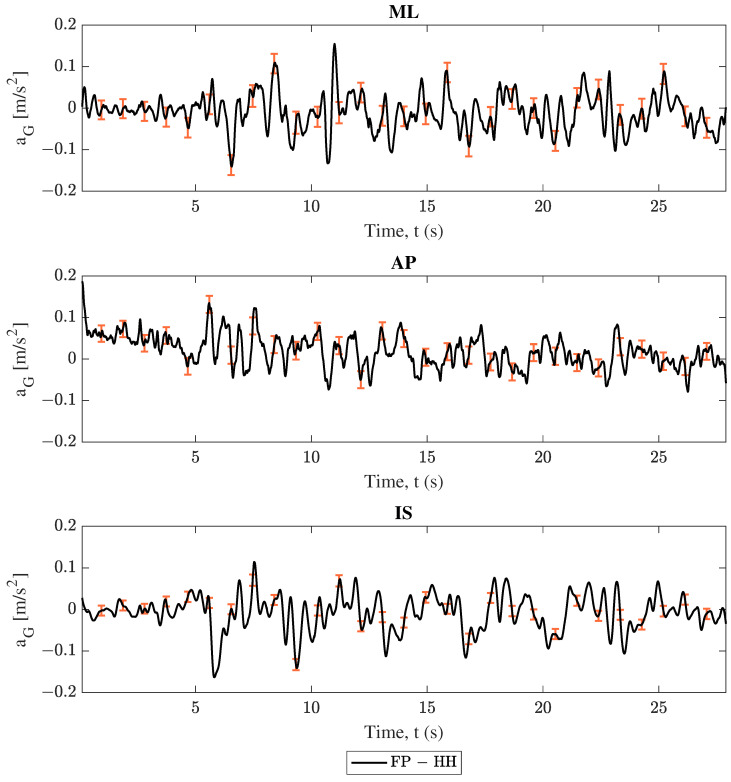
Trial 2 center of mass acceleration ($a_{G}$) difference: force plate (FP) subtracted from handheld (HH) smartphone, where ML, AP, and IS represent the medio–lateral, antero–posterior, and inferior–superior axes respectively. The error bars represent confidence intervals.

**Figure 7:**
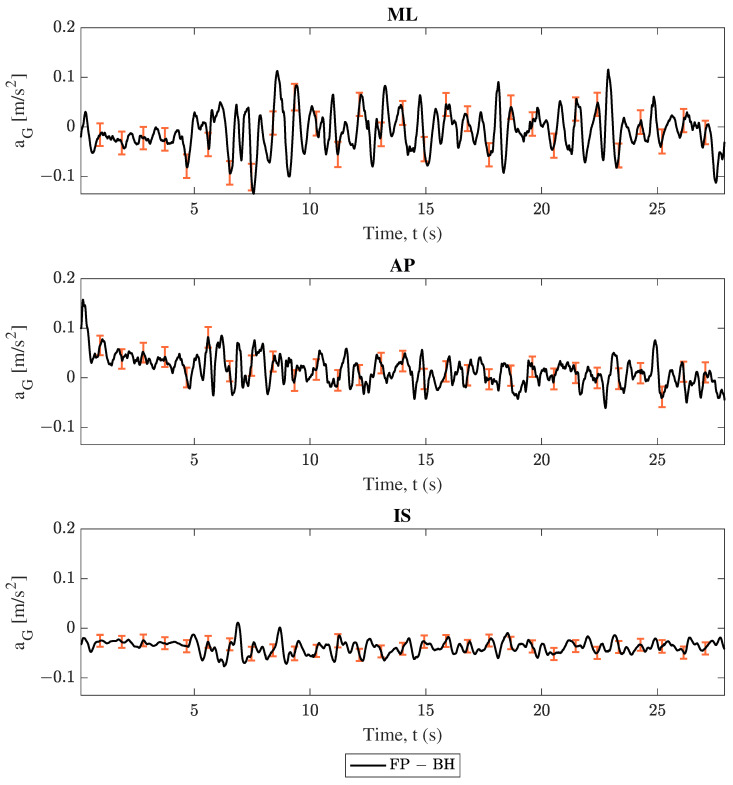
Trial 2 center of mass acceleration ($a_{G}$) difference: force plate (FP) subtracted from back harness (BH) smartphone, where ML, AP, and IS represent the medio–lateral, antero–posterior, and inferior–superior axes respectively. The error bars represent confidence intervals.

As described in Section 2, COM acceleration estimations were compared between each phone and the force plate. Projections from both smartphones followed Equation (1), and force plate data followed Equation (5). The comparison between these results for trial 2 (swaying in the medio–lateral direction) are shown in Figure 4, Figure 5, Figure 6 and Figure 7. Force plate (FP) results are compared to the handheld (HH) and back harness (BH) smartphones in Figure 4 and Figure 5, respectively. The differences of results plotted in Figure 4 and Figure 5 are shown in Figure 6 and Figure 7, respectively. All smartphone curves include confidence intervals computed following Section 2.6.

All data in Figure 4, Figure 5, Figure 6 and Figure 7 show moderate agreement between the smartphone and force plate results following the criteria described in Section 2.3. For the “engaged” medio–lateral (ML) direction, Figure 4 and Figure 5 show that the force plate data curves generally stay within the plotted uncertainty limits of the smartphone data, while Figure 6 and Figure 7 show the difference curve’s consistently capturing 0 in the uncertainty window. However, the “unengaged” antero-posterior (AP) direction and the inferior-superior (IS) component showed limited accuracy and in some cases disagreement between the force plate and smartphone data. These trends are observed irrespective of smartphone considered as well as in the other trials found in the Supplementary Figures, with engaged directions showing moderate agreement and unengaged directions having limited accuracy.

### 3.2. Center of Pressure Comparison

**Figure 8:**
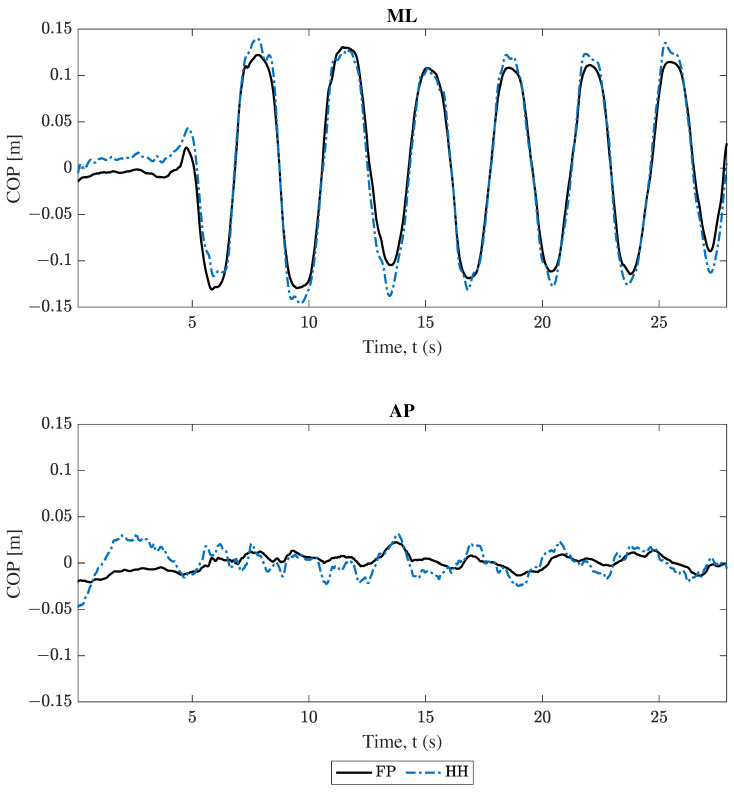
Trial 2 COP position comparison. Force plate (FP) versus handheld (HH) smartphone, where ML and AP represent the medio–lateral and antero–posterior axes, respectively.

**Figure 9:**
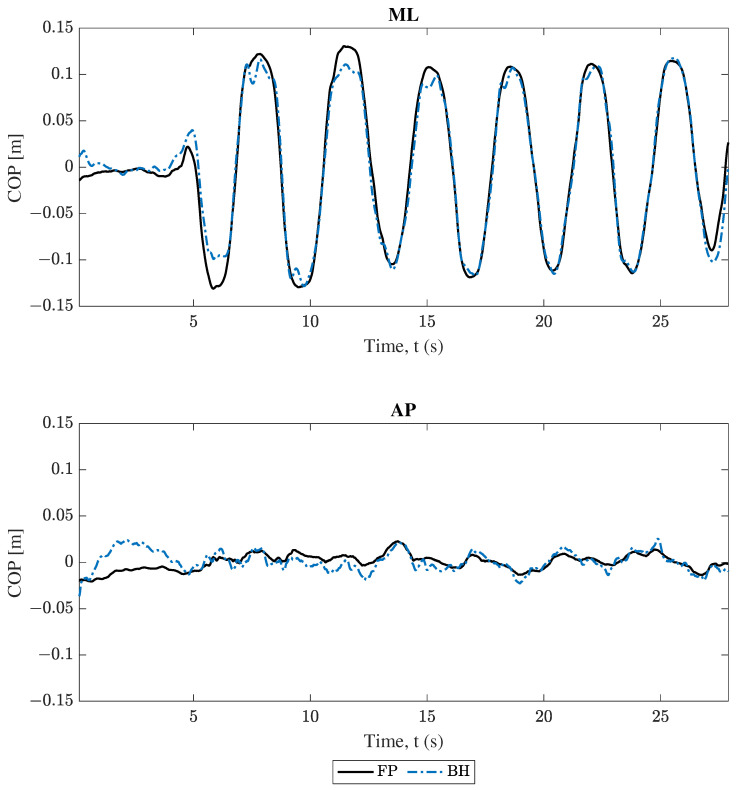
Trial 2 COP position comparison. Force plate (FP) versus back harness (BH) smartphone, where ML and AP represent the medio–lateral and antero–posterior axes, respectively.

Following completion of all COM analysis, COP position estimations were compared. Projections from both smartphones followed Equation (8) and were compared to data from force plates. The comparison between these results for trial 2 (swaying in the medio–lateral direction) are plotted in Figure 8 and Figure 9, where force plate (FP) results are compared to the handheld (HH) and back harness (BH) smartphones, respectively. Both Figure 8 and Figure 9 show good agreement for the “engaged” ML component and poor agreement for the “unengaged” AP direction for both handheld and back harness phones. These same trends hold for the other trials found in the Supplementary Figures.

### 3.3. Data Correlation

Further inspection of trial data was found through application of the Spearman’s rank correlation coefficient. A correlation coefficient was found comparing the data depicted in each result’s figure subplot, as described in Section 2.7. Table 2 shows the Spearman’s rank correlation coefficient between the force plate and each smartphone result for all trials, as well as their respective *p* values. For all trials and both phones, engaged directions exhibit correlation values greater than 0.95, while the unengaged directions have values in the range of 0.4–0.6, and finally, the IS direction has correlation values of 0. When comparing smartphones, the correlation coefficients are all within 0.04 of each other for engaged directions, with the highest value varying by trial and axis.

### 3.4. Root Mean Square Error

The agreement of trial data was also verified by computing the root mean square error (RMSE) between the force plate and smartphone data. Table 3 shows the RMSE between the force plate and each smartphone result for all trials, directions, and both models (COM acceleration and COP position). For all trials, models, and phones, no clear difference in error is found between the engaged and unengaged directions. For example, trial 2 shows the RMSE of the “engaged” ML direction and the “unengaged” AP direction are all within 0.0054 of each other, with neither error consistently smaller or larger. In contrast, compared by model, the RMSE of the COP position is persistently below the COM acceleration’s RMSE (by a factor of 3–10) for all directions, trials, and smartphones. Lastly, comparing by phone, no consistent difference is found between RMSE values, with neither phone’s error smaller across all trials, directions, and models.

## 4. Discussion

### 4.1. Key Findings

All data in Figure 4, Figure 5, Figure 6, Figure 7, Figure 8 and Figure 9 and Table 2 and Table 3 show moderate agreement between the smartphone and force plate results for both models (conversion of smartphone data to COM acceleration and determination of the COP position from COM acceleration) and both smartphones (handheld and back harness phones). For example, trial 2’s (ML direction swaying) COM acceleration plots (Figure 4, Figure 5, Figure 6 and Figure 7) show good agreement for the “engaged” ML component, though limited agreement for the “unengaged” AP direction. COP position plots (Figure 8 and Figure 9) show similar trends, with good agreement in engaged directions and limited agreement in unengaged components. The Spearman’s rank correlation coefficients for both models (Table 2) reflect this observation, with higher correlation values for engaged directions over the unengaged values. The root mean square error (RMSE) in Table 3 initially fails to show the same distinction between engaged and unengaged direction, with instead comparable RMSE for all directions. However, since the RMSE is an absolute error, the similar RMSE between engaged and unengaged components indicates a *smaller relative error* for the larger valued, engaged direction data. For example, by normalizing trial 2’s RMSE of the handheld COM acceleration by the corresponding component’s range, we obtain relative RMSEs of 0.0655 and 0.2459 for the ML (engaged) and AP (unengaged) directions, respectively. All these findings indicate both models and smartphones are successfully able to measure postural sway with comparable accuracy to the “gold standard” force plates.

Finally, it is apparent from the data figures as well as the coefficient table that the COM acceleration showed poor performance in the IS direction. This performance is due to the limitations of the inverted pendulum model used to generate COM accelerations from the force plate COP position. This limitation is further discussed in Section 4.4. Additionally, the magnitude of the analyzed vectors also showed poorer coefficient scores, but this should be expected since the limited accuracy of the unengaged and IS directions contributed to the magnitude’s behavior.

### 4.2. Device Location Comparison: Handheld Versus Back Harness Phone

The first goal of this study was to develop a methodology for the use of smartphones at arbitrary positions and orientations to track postural sway. When comparing the performance of the two smartphone locations (handheld and back harness), little distinction was found. Figure 4, Figure 5, Figure 6, Figure 7, Figure 8 and Figure 9 show that both phones generally report oscillations of the COM acceleration within uncertainty limits of the force plates. When compared by smartphone type, Table 2 shows correlation coefficients all within 0.04 of each other for engaged directions. Additionally, the results’ resolutions are comparable between devices, since the uncertainty ranges were comparable in magnitude for COM acceleration results from all trials and devices. Lastly, Table 3 shows no consistent better performer between the two phones, with the smaller RMSE phone varying across all trials, directions, and model. These findings indicate that the choice of using a handheld phone or one placed in a back harness did not have a significant impact on the model’s performance, accuracy, or precision. Thus, the proposed model allows the mobile device to be held anywhere, rather than having to be placed near the subject’s COM, for a more flexible set-up without compromising accuracy of balance assessment.

### 4.3. Model Results Comparison: COM Acceleration Versus COP Position

The second aim of this study was to propose an analysis technique to transform smartphone COM acceleration measurements into COP displacement for direct comparison with force plates. By comparing the two models (conversion of smartphone data to COM acceleration and determination of the COP position from COM acceleration), it is unclear which shows better agreement with the force plate reference. Spearman’s coefficients in Table 2 do show distinctions between the COM acceleration and COP displacement results, but the lead performer varied between trials. In trial 2, the COP position results show *higher* Spearman’s rank correlation coefficients than COM acceleration results across all axes. However, in trials 3 and 4, COP position results show *lower* Spearman’s rank correlation coefficients compared to COM accelerations across all axes. The difference in coefficients varies from 0.03 to 0.08 in the active direction. The lack of consistency across trials warrants further testing to determine whether trial 2 is an outlier, whether the higher performer is random, or whether there is a factor driving the model’s performance. Lastly, Table 3 shows that the RMSE for the COP position is consistently a factor of 3 to 10 times smaller than the COM acceleration’s RMSE. However, this difference in absolute error is again due to the COP position’s smaller scale, which is also about 3 times smaller. All these results indicate a comparable performance of the two models and that there is no accuracy loss when converting smartphone COM acceleration to COP displacement, allowing for the use of COP metrics from mobile device measurements.

### 4.4. Limitations

#### 4.4.1. Model Limitations

The formulation of both models (conversion of smartphone data to COM acceleration and determination of the COP position from COM acceleration) contained several limiting assumptions, including the treatment of the subject’s body above the ankles as one rigid member (rigid body kinematics, Section 2.3.1) and the assertion that bending in the subject’s body only takes place at the ankles with small angular rotations about that pivot point (inverted pendulum, Section 2.3.2 and Section 2.3.3). These assumptions explain two of the erroneous behaviors found in the results. First, in the engaged directions, the smartphone COM acceleration results of both models tend to overshoot the force plate metric during peak motion (see Figure 4, Figure 5, Figure 6 and Figure 7). During swaying, it is likely the subject is bending at their midsection, which would cause points above the COM to undergo increased motion compared to the COM. Since both mobile devices are above the subject’s COM, the midsection bending would cause the smartphones to report larger accelerations, and consequently larger COM accelerations. Thus, the model’s limitations on the body being rigid for the purposes of rigid body kinematics and inverted pendulum application prevent it from accounting for this bending. Second, in all COM acceleration figures, the force plates reported no acceleration in the IS direction, resulting in no correlation between IS results for any trial (Table 2). The supposition that the body only undergoes small angular rotations about the ankle results in a restriction of the COM acceleration to the lateral plane for outputs of the inverted pendulum model, resulting in only zero values for the force plate’s COM acceleration in the IS direction. Thus, the use of this model prevents any accurate validation of the COM acceleration results in the IS direction. Additionally, since the inverted pendulum model is used to convert the smartphone’s COM acceleration data into the COP position, the mobile device’s non-zero IS acceleration components could drive undesirable behavior in the COP displacement results. Further testing on the effects the IS components of the COM acceleration have on the methodology is required.

Beyond performance results, the inverted pendulum model also limits the types of trials that are possible using this methodology. The model requires that both feet are side-by-side in full contact with the ground, restricting the balance tests possible. Researchers have found ways to account for variations in stance width [28], but further pose validation is necessary to extend the model to other balance poses, such as the single-leg stance or tandem stance.

#### 4.4.2. Filtering

The utility of the model also depends on the determination of the cut-off frequency for all filtering steps. For this study, good performance was found with a low-pass cut-off frequency of 2 Hz applied to the smartphone data, which is relatively low for balance evaluation [6,34,35]. This increased performance could be due to its proximity to the rate of oscillation in the engaged direction(s) to the cut-off frequency. It is also possible that bending at the midsection took place at frequencies just higher than 2 Hz, meaning error from the model’s limiting assumptions could be mitigated by this low-pass filtering. In any case, applications of low-pass filtering around 2 Hz will dampen the mid-ranged frequencies analyzed for patient diagnosis, such as those in the 5–10 Hz range, which can indicate tremors from Parkinson’s disease [20]. For subjects exhibiting notable behaviors at these mid-range frequencies, further testing is needed to see whether the model performs well in this range despite behaviors from the model’s limiting assumptions.

### 4.5. Future Work

The model showed good sway tracking for moderate ranges of motion and limited performance for peak motion and static (good) balance. Further investigation of this performance is necessary to understand the current model for clinical usage. First, additional trials of varying oscillation frequency and amplitude can be used to validate key findings and further define the scope of the model’s limitations. Second, testing of the model on a wider range of subjects, some of whom exhibit balance impairments, will serve to further validate the model’s good and poor performance ranges. Particularly, the subject tested here was a healthy adult, who exhibited good balance in the unengaged direction when swaying; thus, the observed poor performance in the unengaged direction may become less pronounced when testing on a subject with impaired balance. Third, the methodology can easily be tested on trials more common in clinical testing, such as standing still with eyes closed, to directly test for clinical application. Lastly, the final COP data acquired (COP displacement) can be post-processed into other common balance metrics, such as COP velocity RMS, maximum COP velocity, or 95% Confidence Ellipse Area [2,3,39,40,41,42]. These additional metrics will only involve one numerical derivative, following Section 2.4, and minor manipulation of the resulting time series, but will serve as a more robust metric of balance performance used widely in previous balance research [2,39,40,41,42].

Further adjustments could also be made to address limitations described in Section 4.4. One such extension is the double inverted pendulum model proposed by researchers [43] to allow for bending at the ankles *and* waist. The double inverted pendulum model could help diminish the overshoot observed at the peak COM acceleration. The inclusion of the double inverted pendulum could also mitigate the low cut-off frequency described in Section 4.4.2. The low cut-off frequency was selected due to mid-frequency responses from the mobile device measurements; since these responses were not detected in the force plate data, it is possible that they are caused by rotations about the waist, as described in Section 4.4. Hence, application of a double inverted pendulum model would account for the bending, allowing for a higher cut-off frequency, which would be useful for clinical balance assessments [20].

Lastly, the accessibility of the model’s performance is limited by the use of an expensive motion capture system to determine various position vectors. This limitation could be removed through the use of motion tracking tools such as the Microsoft Kinect (Microsoft, Redmond, WA, USA), or computer vision tools such as OpenCV (Version 4.11.0, Palo Alto, CA, USA) and DeepLabCut (UPMWMATHIS LAB, EPFL, Geneva, Switzerland). Researchers have recently shown the ability to use Kinect’s motion tracking systems for human biomechanics research [44]. DeepLabCut, an image recognition tool, has also been used to track the motion of human and animal anatomy [45,46], though the tool has only been implemented using one video feed to perform 2D tracking or pose recognition. Low-cost solutions like these would allow for the 3D position tracking necessary for the rigid body kinematics model without the use of an expensive camera system. Alternatively, estimates of the position vector could be obtained through anatomical measurements [23,26], eliminating the need for a camera system entirely. Applying these anatomical measurements would allow the model outlined in this paper to reproduce this study’s results using only a smartphone and participant information. Replacing motion capture cameras with smartphone cameras, or removing 3D position tracking entirely, would pave the way for a fully mobile balance laboratory.

## 5. Conclusions

This study investigated the novel approach of determining COM accelerations for balance assessments using mobile devices and further used these results, in conjunction with the inverted pendulum model, to produce COP displacements. Smartphone IMU data taken in two locations (handheld and in a harness) were compared to force plate data in balance experiments with one healthy subject, engaging in swaying motion in different anatomical directions. Smartphone results for both COM acceleration and COP displacements showed a good correlation with force plate results along engaged directions of motion. Performance was nearly indistinguishable between the handheld and back harness smartphones, indicating that the increase in flexibility of having a handheld smartphone had no notable effect on the model’s performance. Both stages of the model (COM acceleration and COP displacement) also performed equally well, as shown by the correlation results. However, the model showed limitations through lower accuracy in unengaged directions, overshooting during peak motions, and relatively low cut-off frequencies for filtering. These limitations are attributed to the assumptions of the inverted pendulum model not accounting for bending at the waist. Future work expanding to the double-inverted pendulum model could account for these limitations. Additional future research should be conducted to perform robust testing of the methodology on a larger sample size of participants and improve the analysis’ accessibility by removing dependence on motion capture systems.

## Figures and Tables

**Table 1:** Description of motion performed for each trial. Trial 2 (in bold) used for results in Section 3, with the remaining trials’ results included in Supplementary Figures S1–S18.

Trial	Engaged Direction	Motion Description
1	None	Stable balance
**2**	**ML**	**Swaying in medio–lateral direction**
3	AP	Swaying in antero–posterior direction
4	Both	Random swaying in both directions

**Table 2:** Spearman’s rank correlation coefficients (ρ) and *p*-values between force plate and smartphone’s resultant COM acceleration and COP position. Correlation results are broken down by phone (handheld and back harness), trial number (No.), vector components (Comp.), and vector magnitude (Norm).

Trial		COM Acceleration				COP Position			
No.	Comp.	Handheld		Back Harness		Handheld		Back Harness	
		ρ	p	ρ	p	ρ	p	ρ	p
**1**	**Norm**	0.271	0.000	0.334	0.000	−0.133	0.000	−0.018	0.446
	**ML**	0.404	0.000	0.566	0.000	0.385	0.000	0.6949	0.0000
	**AP**	0.333	0.000	0.470	0.000	−0.102	0.000	−0.1594	0.0000
	**IS**	−0.000	1.000	0.000	1.000				
**2**	**Norm**	0.934	0.000	0.915	0.000	0.963	0.000	0.9622	0.0000
	**ML**	0.985	0.000	0.980	0.000	0.988	0.000	0.9883	0.0000
	**AP**	0.442	0.000	0.498	0.000	0.459	0.000	0.4594	0.0000
	**IS**	0.000	1.000	0.000	1.000				
**3**	**Norm**	0.949	0.000	0.874	0.000	0.855	0.000	0.7561	0.0000
	**ML**	0.597	0.000	0.649	0.000	0.521	0.000	0.1587	0.0000
	**AP**	0.991	0.000	0.983	0.000	0.970	0.000	0.9462	0.0000
	**IS**	−0.000	1.000	−0.000	1.000				
**4**	**Norm**	0.742	0.000	0.811	0.0000	0.728	0.000	0.7153	0.0000
	**ML**	0.921	0.000	0.962	0.000	0.910	0.000	0.9503	0.0000
	**AP**	0.956	0.000	0.962	0.000	0.948	0.000	0.9073	0.0000
	**IS**	−0.000	1.000	−0.000	1.000				

**Table 3:** Root mean square error (RMSE) between force plate and smartphone’s COM acceleration and COP position. Errors are broken down by phone (handheld and back harness), trial number (No.), vector components (Comp.), and vector magnitude (Norm).

Trial		COM Acceleration RMSE		COP Position RMSE	
No.	Comp.	Handheld	Back Harness	Handheld	Back Harness
**1**	**Norm**	0.0419	0.0373	0.0049	0.0035
	**ML**	0.0157	0.0194	0.0025	0.0022
	**AP**	0.0300	0.0152	0.0069	0.0053
	**IS**	0.0345	0.0359		
**2**	**Norm**	0.0497	0.0412	0.0137	0.0120
	**ML**	0.0434	0.0393	0.0143	0.0126
	**AP**	0.0451	0.0339	0.0129	0.0102
	**IS**	0.0434	0.0397		
**3**	**Norm**	0.0827	0.0777	0.0235	0.0227
	**ML**	0.0289	0.0316	0.0057	0.0078
	**AP**	0.0775	0.0743	0.0238	0.0235
	**IS**	0.0403	0.0409		
**4**	**Norm**	0.0967	0.0784	0.0178	0.0174
	**ML**	0.0965	0.0665	0.0154	0.0116
	**AP**	0.1056	0.0858	0.0197	0.0204
	**IS**	0.0761	0.0429		

**Figure A1:**
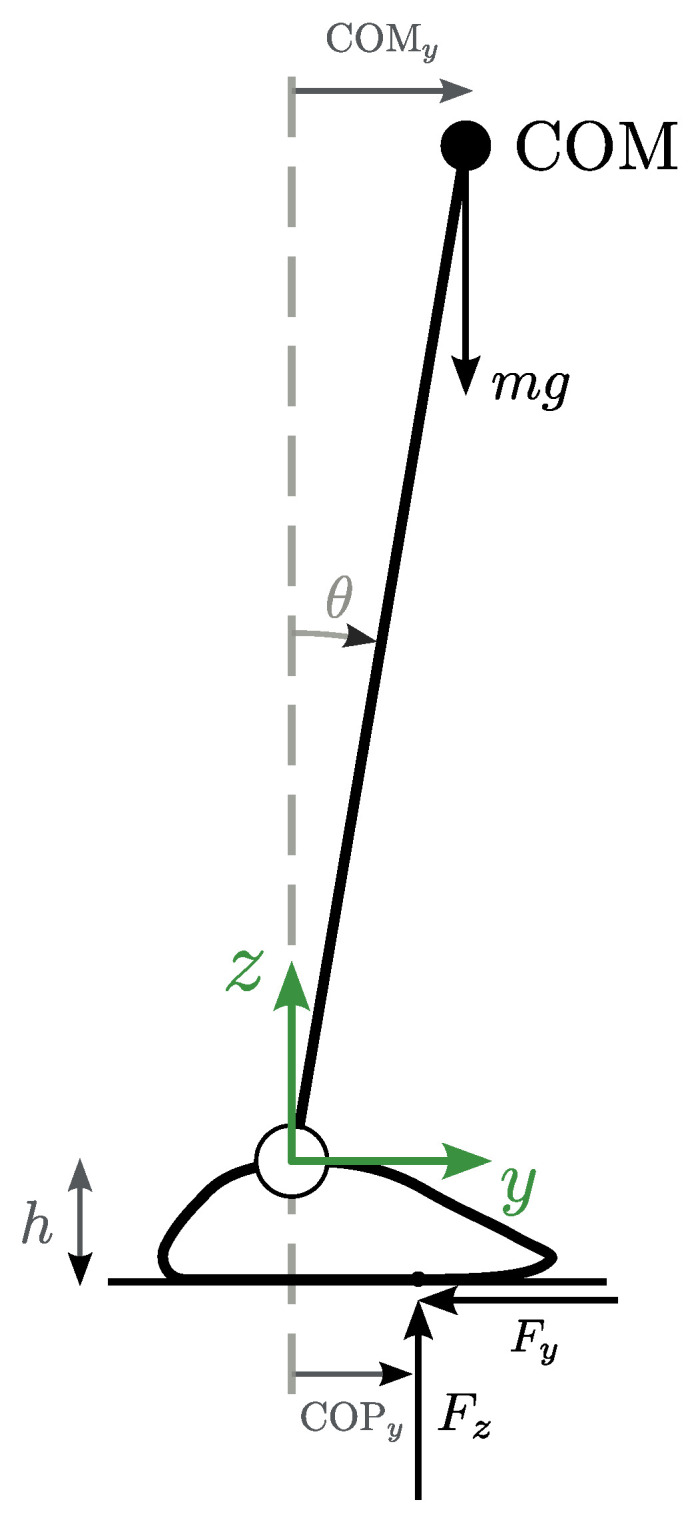
System level free body diagram in inverted pendulum model, side view of sagittal plane.

**Figure A2:**
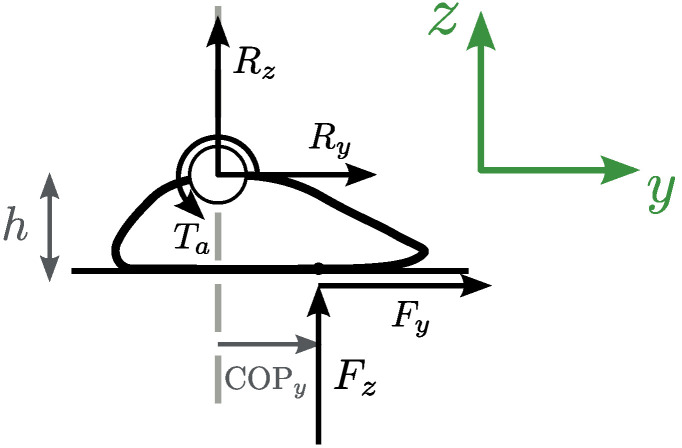
Free body diagram of foot in inverted pendulum model, sagittal view.

**Figure A3:**
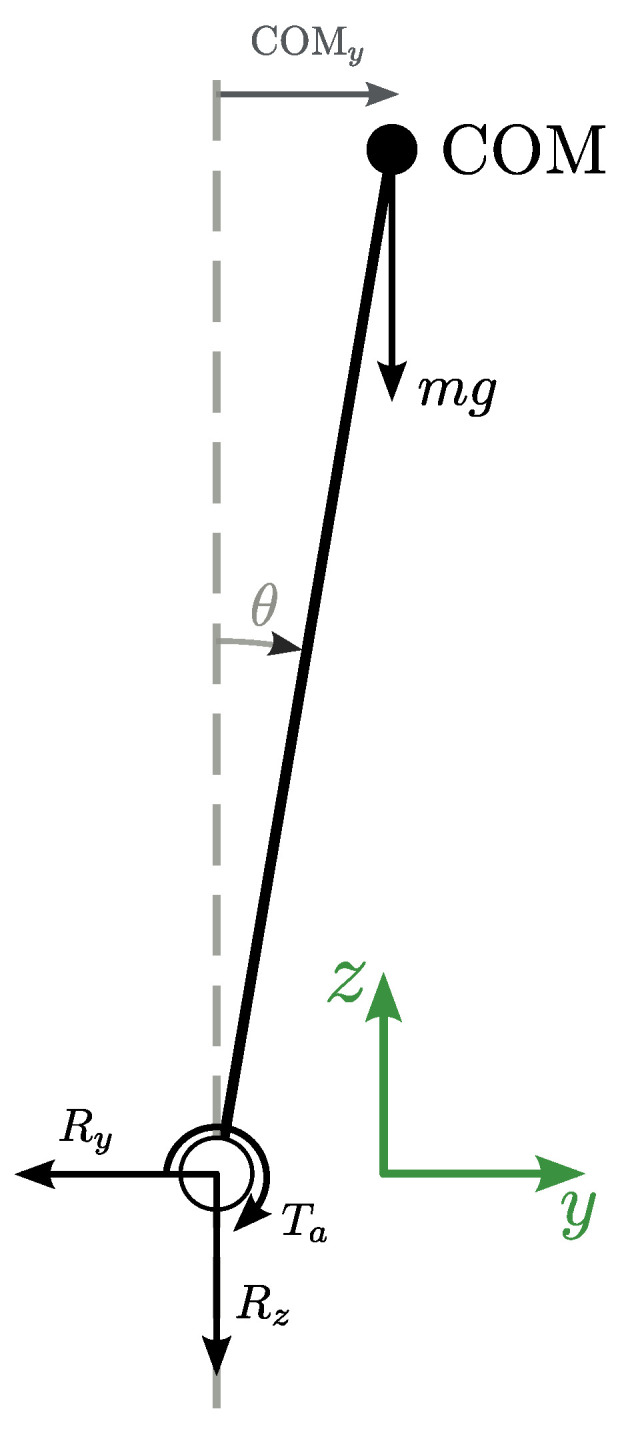
Free body diagram of body (disconnected from foot), sagittal view.

## Data Availability

Please direct data inquiries to Eric Ocegueda, at ocegueda@calpoly.edu.

## Supplementary Materials

The following supporting information can be downloaded at: https://www.mdpi.com/article/10.3390/s25092639/s1. Results figures for additional trials. Figure S1: Trial 1 center of mass acceleration ($a_{G}$) comparison. Force plate (FP) versus handheld smartphone (HH), where ML, AP, and IS represent the medio–lateral, antero–posterior, and inferior–superior axes respectively. The error bars represent confidence intervals. Figure S2: Trial 1 center of mass acceleration ($a_{G}$) comparison. Force plate (FP) versus back harness smartphone (BH), where ML, AP, and IS represent the medio–lateral, antero–posterior, and inferior–superior axes respectively. The error bars represent confidence intervals. Figure S3: Trial 1 center of mass acceleration ($a_{G}$) difference: force plate (FP) subtracted from handheld (HH) smartphone, where ML, AP, and IS represent the medio–lateral, antero–posterior, and inferior–superior axes respectively. The error bars represent confidence intervals. Figure S4: Trial 2 center of mass acceleration ($a_{G}$) difference: force plate (FP) subtracted from back harness (BH) smartphone, where ML, AP, and IS represent the medio–lateral, antero–posterior, and inferior–superior axes respectively. The error bars represent confidence intervals. Figure S5: Trial 1 COP position comparison. Force plate (FP) versus handheld (HH) smartphone, where ML and AP represent the medio–lateral and antero–posterior axes, respectively. Figure S6: Trial 1 COP position comparison. Force plate (FP) versus back harness (BH) smartphone, where ML and AP represent the medio–lateral and antero–posterior axes, respectively. Figure S7: Trial 3 center of mass acceleration ($a_{G}$) comparison. Force plate (FP) versus handheld smartphone (HH), where ML, AP, and IS represent the medio–lateral, antero–posterior, and inferior–superior axes respectively. The error bars represent confidence intervals. Figure S8: Trial 3 center of mass acceleration ($a_{G}$) comparison. Force plate (FP) versus back harness smartphone (BH), where ML, AP, and IS represent the medio–lateral, antero–posterior, and inferior–superior axes respectively. The error bars represent confidence intervals. Figure S9: Trial 3 center of mass acceleration ($a_{G}$) difference: force plate (FP) subtracted from handheld (HH) smartphone, where ML, AP, and IS represent the medio–lateral, antero–posterior, and inferior–superior axes respectively. The error bars represent confidence intervals. Figure S10: Trial 3 center of mass acceleration ($a_{G}$) difference: force plate (FP) subtracted from back harness (BH) smartphone, where ML, AP, and IS represent the medio–lateral, antero–posterior, and inferior–superior axes respectively. The error bars represent confidence intervals. Figure S11: Trial 3 COP position comparison. Force plate (FP) versus handheld (HH) smartphone, where ML and AP represent the medio–lateral and antero–posterior axes, respectively. Figure S12: Trial 3 COP position comparison. Force plate (FP) versus back harness (BH) smartphone, where ML and AP represent the medio–lateral and antero–posterior axes, respectively. Figure S13: Trial 4 center of mass acceleration ($a_{G}$) comparison. Force plate (FP) versus handheld smartphone (HH), where ML, AP, and IS represent the medio–lateral, antero–posterior, and inferior–superior axes respectively. The error bars represent confidence intervals. Figure S14: Trial 4 center of mass acceleration ($a_{G}$) comparison. Force plate (FP) versus back harness smartphone (BH), where ML, AP, and IS represent the medio–lateral, antero–posterior, and inferior–superior axes respectively. The error bars represent confidence intervals. Figure S15: Trial 4 center of mass acceleration ($a_{G}$) difference: force plate (FP) subtracted from handheld (HH) smartphone, where ML, AP, and IS represent the medio–lateral, antero–posterior, and inferior–superior axes respectively. The error bars represent confidence intervals. Figure S16: Trial 4 center of mass acceleration ($a_{G}$) difference: force plate (FP) subtracted from back harness (BH) smartphone, where ML, AP, and IS represent the medio–lateral, antero–posterior, and inferior–superior axes respectively. The error bars represent confidence intervals. Figure S17: Trial 4 COP position comparison. Force plate (FP) versus handheld (HH) smartphone, where ML and AP represent the medio–lateral and antero–posterior axes, respectively. Figure S18: Trial 4 COP position comparison. Force plate (FP) versus back harness (BH) smartphone, where ML and AP represent the medio–lateral and antero–posterior axes, respectively.

## Appendix A. Inverted Pendulum Derivation

The inverted pendulum model has been established in the literature [15,23,28], but a derivation of the model is reiterated here to support a further discussion of its limitations. This model supports motion in all directions, but the derivation will be limited here to the sagittal plane for brevity. It is trivial to repeat this analysis in the coronal plane to complete the model, and the resulting equation of motion is listed here next to its sagittal counterpart.

### Appendix A.1. Assumptions and Definitions

The inverted pendulum model assumes the bottom faces of the feet make full contact with the supporting surface, the point equidistant from both ankles acts as a fixed point of rotation for the body above them, the body from the ankles upward act as a rigid body, and the body undergoes only small rotations about the pivot [15]. Additionally, it is assumed that the participant’s stance is two-legged with feet side by side. Key terms in the derivation are defined in Table A1.

**Table A1:** Specification of terms used in inverted pendulum derivation. Coordinate directions form a right-handed coordinate system with ${\hat{e}}_{x}$, ${\hat{e}}_{y}$, and ${\hat{e}}_{z}$ directed in the lateral, anterior, and superior directions, respectively.

Name	Symbolic Form
Angular Acceleration	$\vec{\alpha }=\alpha_{x}{\hat{e}}_{x}+\alpha_{y}{\hat{e}}_{y}+\alpha_{z}{\hat{e}}_{z}$
COP Position	${\vec{r}}_{COP}=COP_{x}{\hat{e}}_{x}+COP_{y}{\hat{e}}_{y}-h\;{\hat{e}}_{z}$
COM Position	${\vec{r}}_{COM}=COM_{x}{\hat{e}}_{x}+COM_{y}{\hat{e}}_{y}$
Ground reaction force	$\vec{F}=F_{x}{\hat{e}}_{x}+F_{y}{\hat{e}}_{y}+F_{z}{\hat{e}}_{z}$
Angle of Body Rotation	$\vec{\theta }=\theta_{x}{\hat{e}}_{x}+\theta_{y}{\hat{e}}_{y}$
Ankle Height	*h*

### Appendix A.2. Static Analysis

A free body diagram of the foot is shown in Figure A2. Applying a static analysis to the foot in the sagittal plane, the torque exerted at the ankle by the rest of the body ($T_{a}$) is found by summing moments about the ankle, point *a*.

(A1) $$\begin{array}{cc} {\vec{T}}_{a} & =-{\vec{r}}_{COP}\times \vec{F}\\ \; & =\left(-hF_{y}-COP_{y}F_{z}\right){\hat{e}}_{x}+\left(COP_{x}F_{z}+hF_{x}\right){\hat{e}}_{y}+\left(COP_{y}F_{x}-COP_{x}F_{y}\right){\hat{e}}_{z} \end{array}$$

For the sagittal view shown in Figure A1, only the ${\hat{e}}_{x}$ component is considered.

(A2) $${\vec{T}}_{a}\cdot {\hat{e}}_{x}\;=-hF_{y}-COP_{y}F_{z}$$

Assuming $hF_{y}\ll COP_{y}F_{z}$,

(A3) $${\vec{T}}_{a}\cdot {\hat{e}}_{x}\;=-mg\;COP_{y}$$

### Appendix A.3. Dynamic Analysis

Next, a dynamic analysis of the body above the ankles is considered. A diagram of this member is shown in Figure A3. The following assumptions are applied to simplify the dynamic analysis: the body is rigid above the ankles, allowing for the use of rigid body dynamics. The body is symmetric about the sagittal plane to trivialize the use of the inertia tensor. The ankle is considered a fixed point of rotation for the body above it, simplifying the analysis of the torque and COM motion. A small angle approximation constrains the COM acceleration to a lateral plane (i.e., vertical accelerations are assumed to be zero).

As a preliminary step, the body’s kinematics are considered. To determine angular acceleration α in terms of the COM position, the geometric relation is considered:

(A4) $$\sin\theta_{x}=\frac{COM_{y}}{d}$$

where $d=∥{\vec{r}}_{COM}∥$. Differentiating twice with respect to time gives an expression for the body’s angular acceleration, α.

(A5) $$(\cos\theta_{x}){\ddot{\theta }}_{x}-(\sin\theta_{x}){{\dot{\theta }}_{x}}^{2}=\frac{1}{d}\;\left({\vec{a}}_{G}\cdot {\hat{e}}_{y}\right)$$

where ${\vec{a}}_{G}=\frac{d^{2}}{dt^{2}}\left({\vec{r}}_{COM}\right)$. Substituting ${\ddot{\theta }}_{x}=-\alpha_{x}$ and applying a small angle assumption to $\theta_{x}$ reduces the first term and removes the second.

(A6) $$\alpha_{x}=-\frac{1}{d}\;{\vec{a}}_{G}\cdot {\hat{e}}_{y}$$

Next, applying dynamic analysis to Figure A3, the torque exerted at the ankle is found to be

(A7) $${\vec{T}}_{a}\cdot {\hat{e}}_{x}=-I_{xx}\alpha_{x}{\hat{e}}_{x}-mg\;COM_{y}\;{\hat{e}}_{x}$$

where $I_{xx}$ and $\alpha_{x}$ are the mass moment of inertia and angular acceleration of the body about the ${\hat{e}}_{x}$ direction. Substituting Equation (A6) for $\alpha_{x}$:

(A8) $${\vec{T}}_{a}\cdot {\hat{e}}_{x}=\frac{I_{xx}}{d}\left({\vec{a}}_{G}\cdot {\hat{e}}_{x}\right){\hat{e}}_{x}-mg\;COM_{y}{\hat{e}}_{x}$$

### Appendix A.4. Linking Static and Dynamic Results

Equating the above two solutions for ${\vec{T}}_{a}\cdot {\hat{e}}_{x}$ results in

(A9) $$mg\;COP_{y}{\hat{e}}_{x}=\frac{-I_{xx}}{d}\left({\vec{a}}_{G}\cdot {\hat{e}}_{x}\right){\hat{e}}_{x}+mg\;COM_{y}{\hat{e}}_{x}.$$

Rearranging this equation results in the inverted pendulum ODE’s found in the literature [15,23]. The following equations show the result of this rearrangement, as well as the result for analysis of the coronal view.

(A10) $$\begin{array}{cc} I_{xx}\left({\vec{a}}_{G}\cdot {\hat{e}}_{x}\right) & =mgd\left(COM_{y}-COP_{y}\right)\\ I_{yy}\left({\vec{a}}_{G}\cdot {\hat{e}}_{y}\right) & =mgd\left(COM_{x}-COP_{x}\right) \end{array}$$
