# Supplementary material for: Validation of Smartphones in Arbitrary Positions Against Force Plate Standard for Balance Assessment
Source: Sensors (Basel). 2025 Apr 22;25(9):2639. doi: 10.3390/s25092639 (PMC12073742; doi:10.3390/s25092639)
Supplement: Supplementary file 1 [file sensors-25-02639-s001.zip › Supplementary.pdf]

1 Supplementary Results Figures

Results figures for the remaining Trials 1 (stable balance), 3 (swaying in antero–posterior direction) and 4 (random swaying) listed in Table 1 are provided here.

1.1 Trial 1

Center of Mass Acceleration Comparison

As described in Section 2 of the paper, COM acceleration estimations were compared between each phone and the force plate. The comparison between these results for Trial 1 (stable balance) are shown in Figures S1 – S4. Force plate results (FP) are compared to the handheld (HH) and back harness (BH) smartphones in Figures S1 and S2, respectively. The differences of results plotted in Figures S1 and S2 are shown in Figures S3 and S4, respectively.

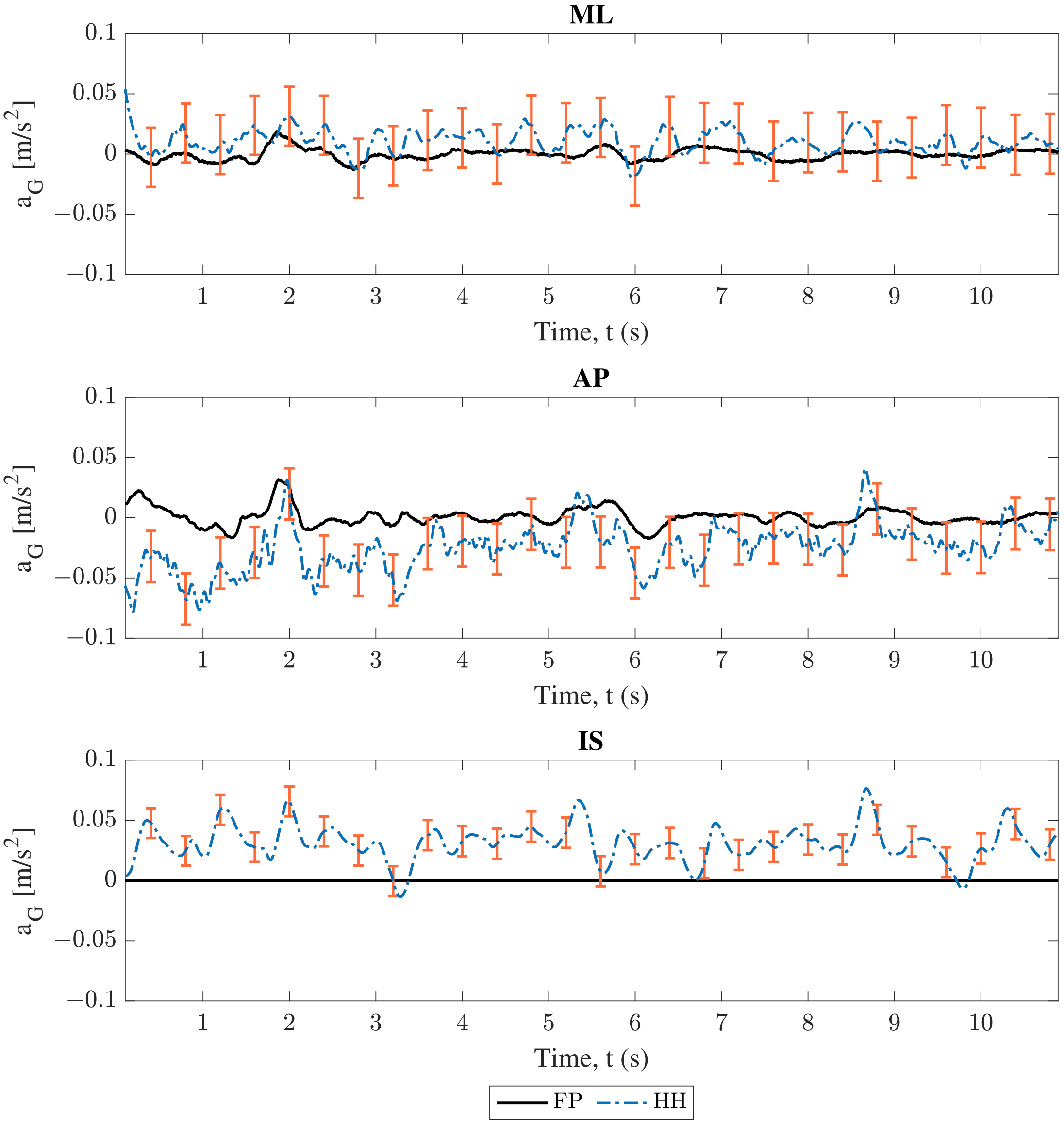

Figure S1: Trial 1 center of mass acceleration (a_G) comparison. Force plate (FP) versus handheld smartphone (HH), where ML, AP, and IS represent the medio–lateral, antero–posterior, and inferior–superior axes respectively. The error bars represent confidence intervals.

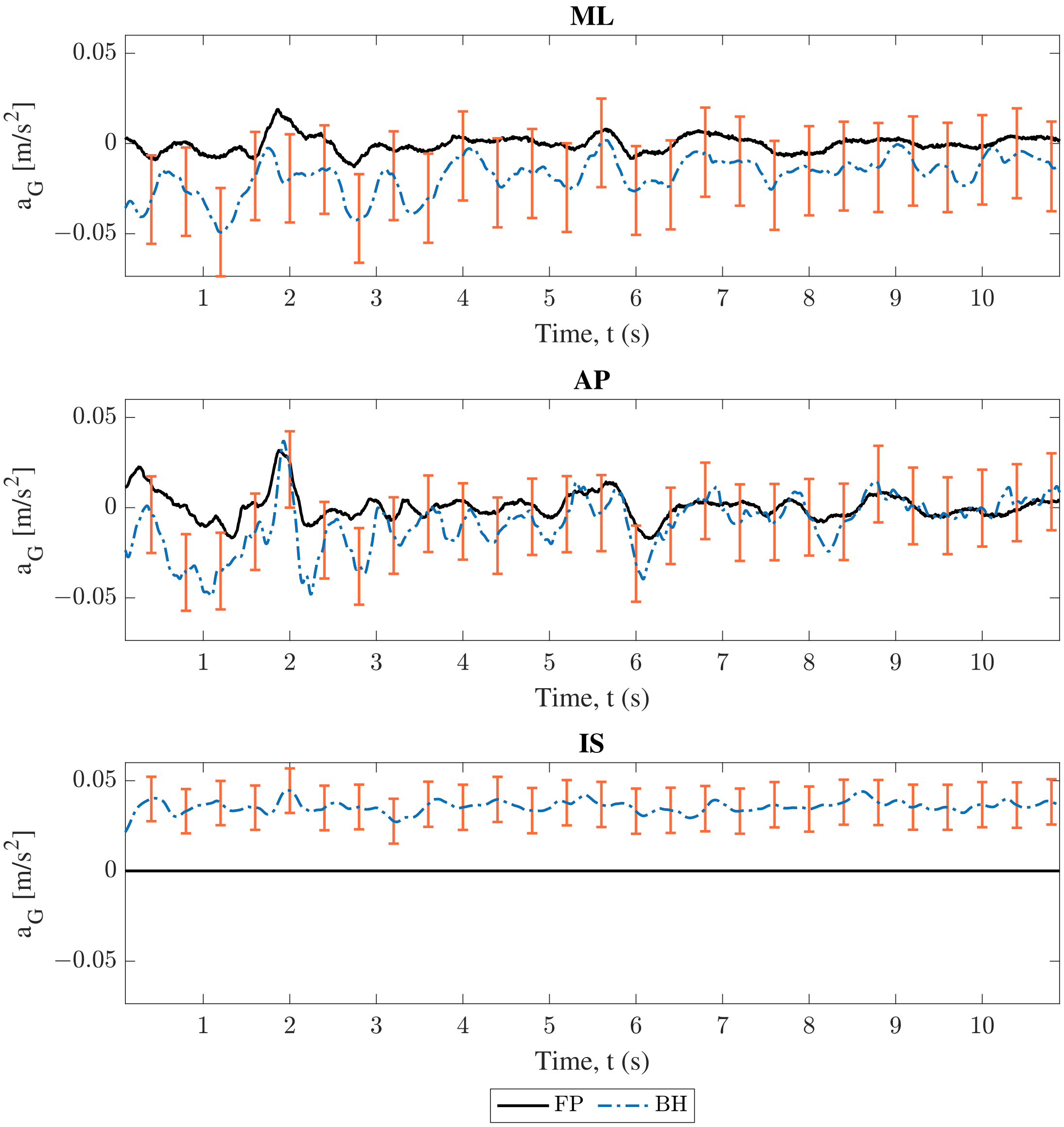

Figure S2: Trial 1 center of mass acceleration (a_G) comparison. Force plate (FP) versus back harness smartphone (BH), where ML, AP, and IS represent the medio–lateral, antero–posterior, and inferior–superior axes respectively. The error bars represent confidence intervals.

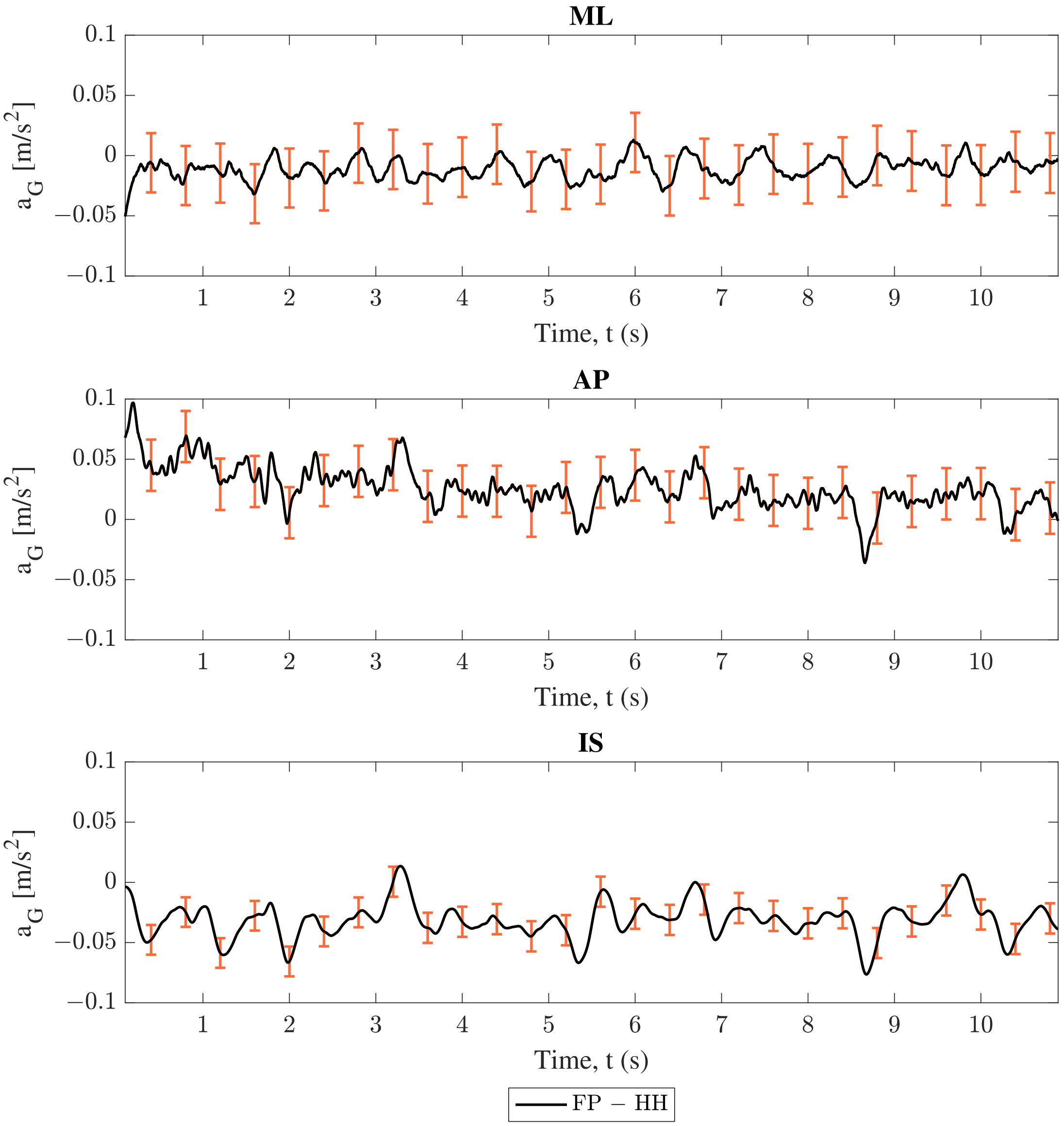

Figure S3: Trial 1 center of mass acceleration (a_G) difference: force plate (FP) subtracted from handheld (HH) smartphone, where ML, AP, and IS represent the medio–lateral, antero–posterior, and inferior–superior axes respectively. The error bars represent confidence intervals.

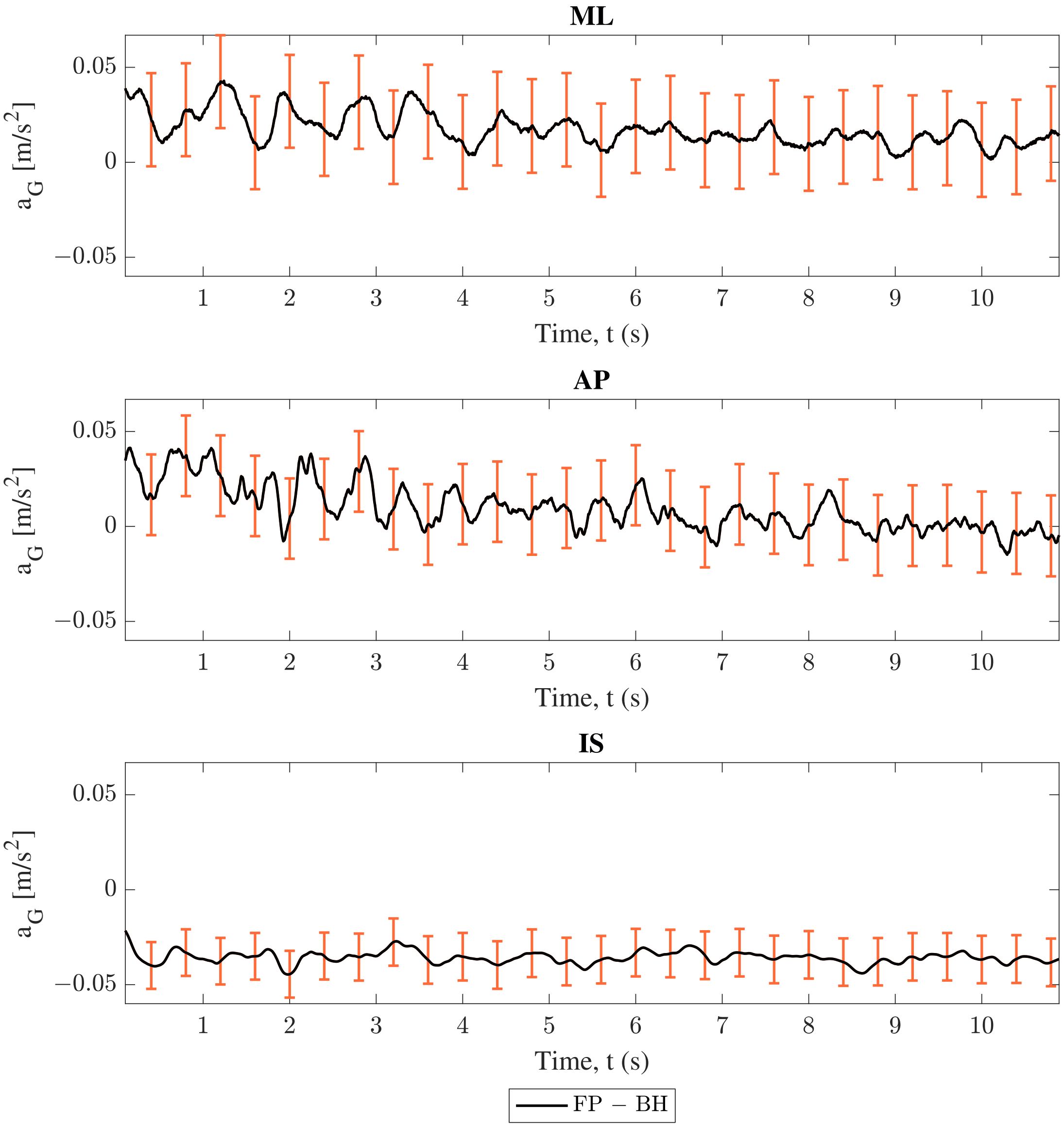

Figure S4: Trial 2 center of mass acceleration (a_G) difference: force plate (FP) subtracted from back harness (BH) smartphone, where ML, AP, and IS represent the medio–lateral, antero–posterior, and inferior–superior axes respectively. The error bars represent confidence intervals.

Center of Pressure Comparison

Center of pressure projections from smartphones were compared to force plate data. The comparison between these results for Trial 1 (stable balance) are plotted in Figures S5 and S6, where force plate results (FP) are compared to the handheld (HH) and back harness (BH) smartphones, respectively.

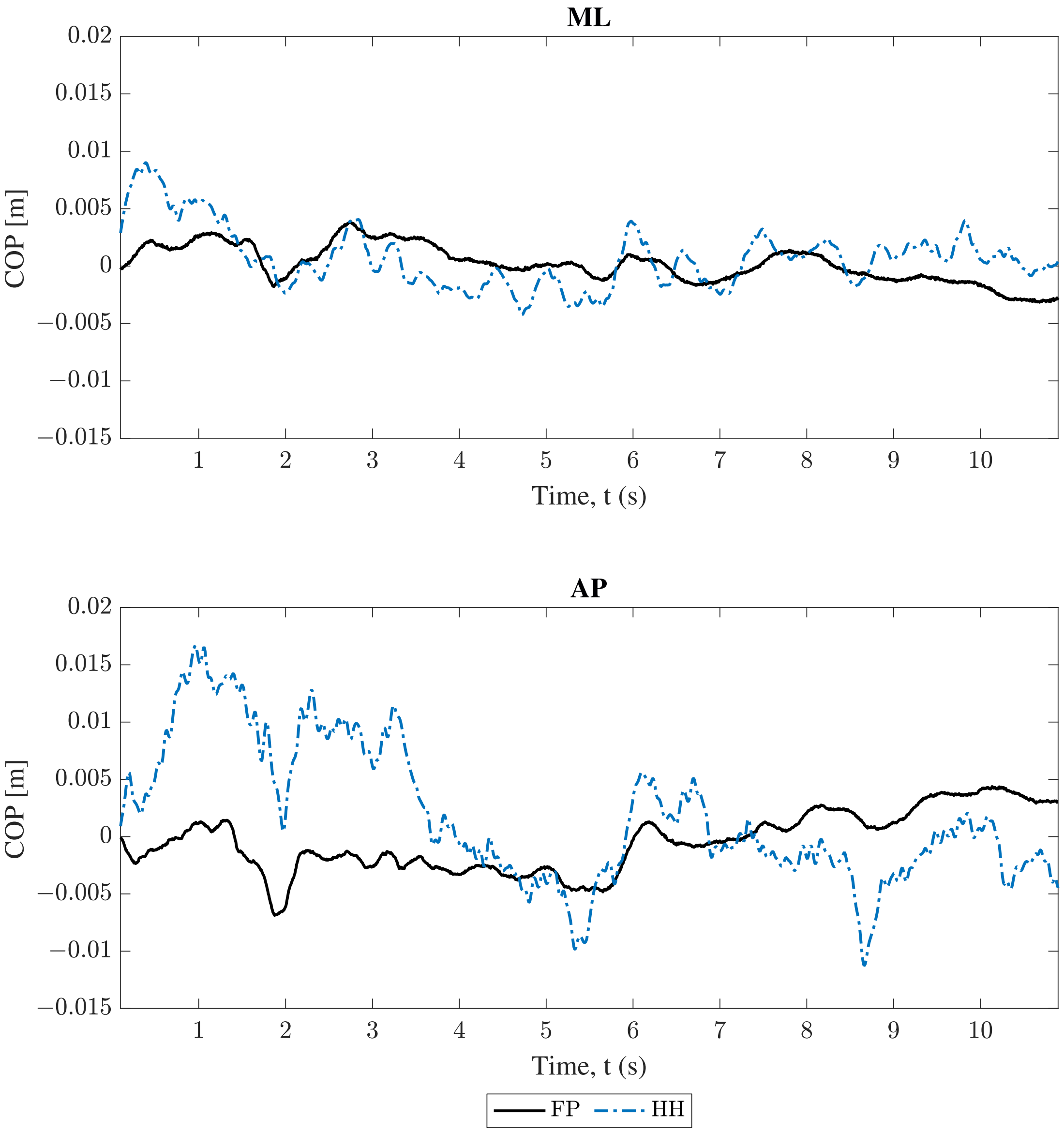

Figure S5: Trial 1 COP position comparison. Force plate (FP) versus handheld (HH) smartphone, where ML and AP represent the medio–lateral and antero–posterior axes, respectively.

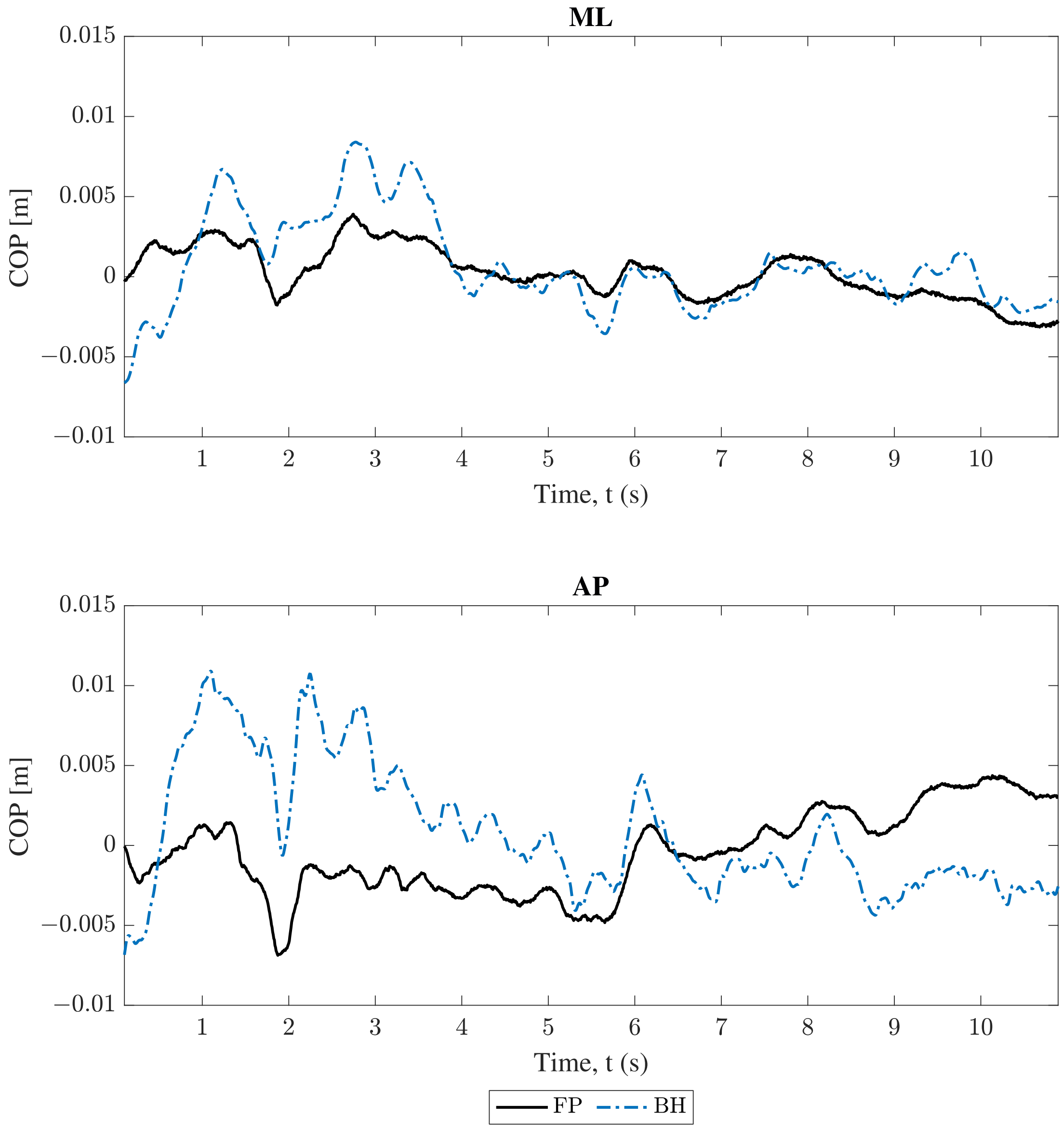

Figure S6: Trial 1 COP position comparison. Force plate (FP) versus back harness (BH) smartphone, where ML and AP represent the medio–lateral and antero–posterior axes, respectively.

1.2 Trial 3

Center of Mass Acceleration Comparison

As described in Section 2 of the paper, COM acceleration estimations were compared between each phone and the force plate. The comparison between these results for Trial 3 (swaying in antero–posterior direction) are shown in Figures S7 – S10. Force plate results (FP) are compared to the handheld (HH) and back harness (BH) smartphones in Figures S7 and S8, respectively. The differences of results plotted in Figures S7 and S8 are shown in Figures S9 and S10, respectively.

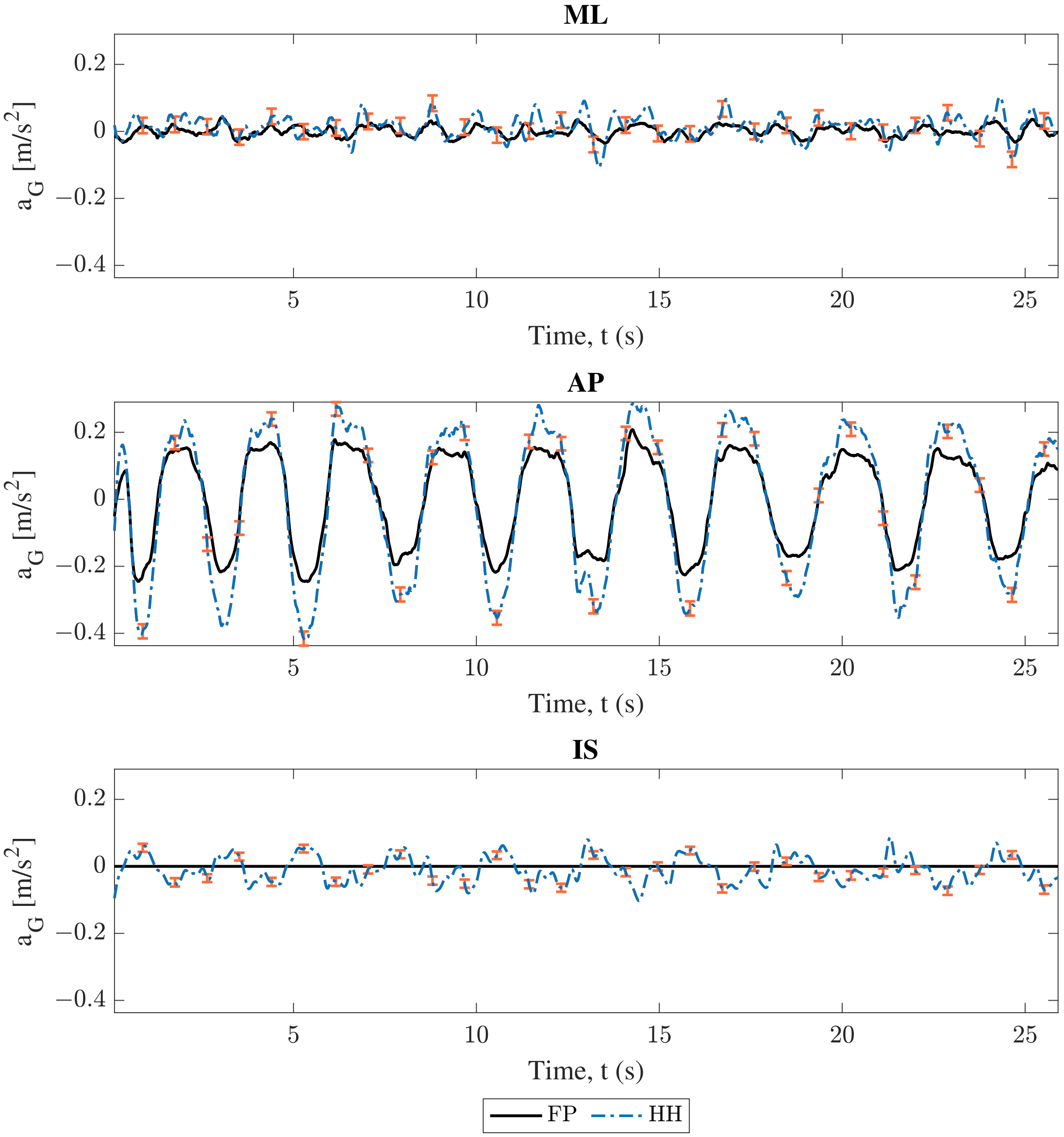

Figure S7: Trial 3 center of mass acceleration (a_G) comparison. Force plate (FP) versus handheld smartphone (HH), where ML, AP, and IS represent the medio–lateral, antero–posterior, and inferior–superior axes respectively. The error bars represent confidence intervals.

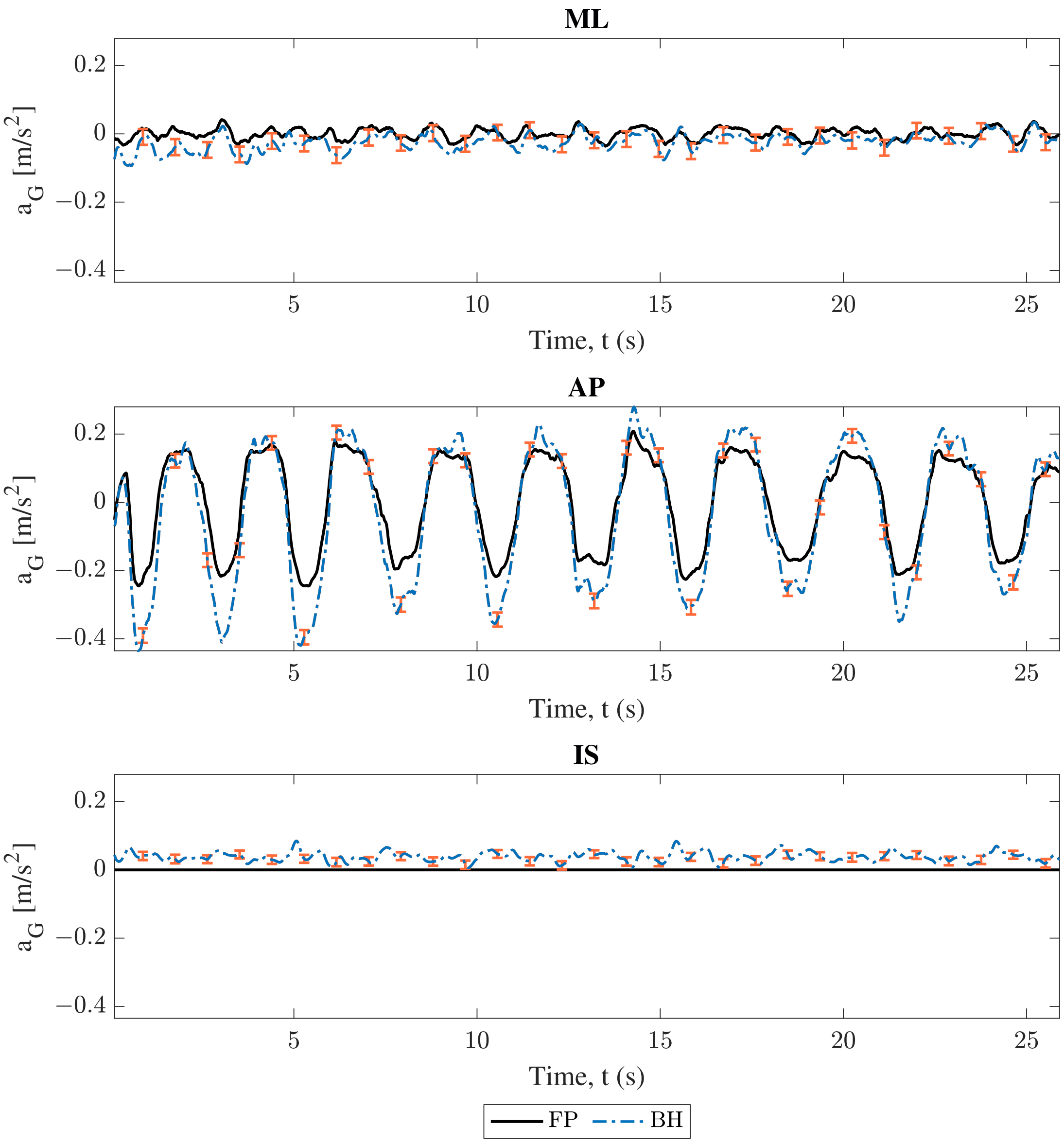

Figure S8: Trial 3 center of mass acceleration (a_G) comparison. Force plate (FP) versus back harness smartphone (BH), where ML, AP, and IS represent the medio–lateral, antero–posterior, and inferior–superior axes respectively. The error bars represent confidence intervals.

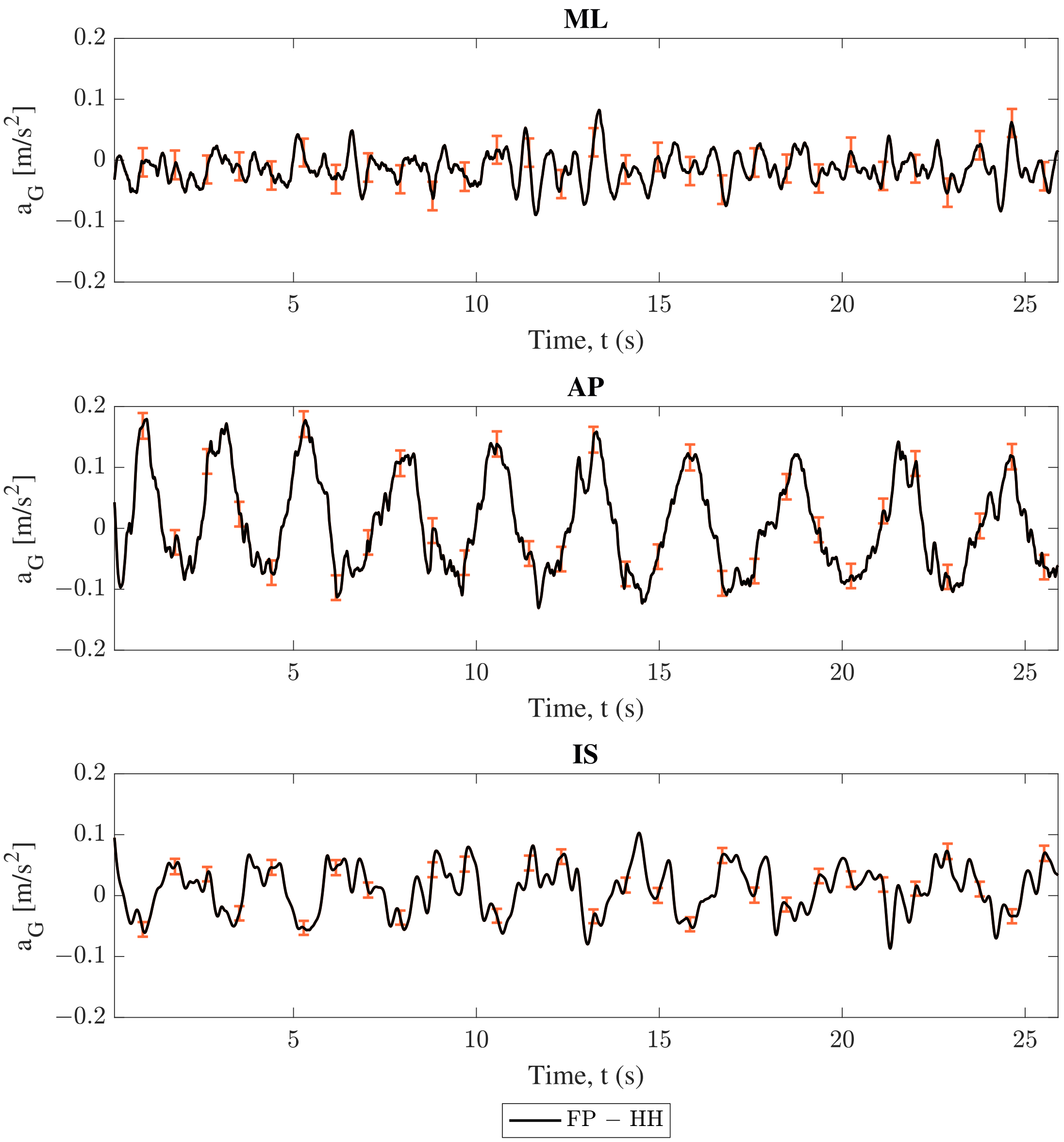

Figure S9: Trial 3 center of mass acceleration (a_G) difference: force plate (FP) subtracted from handheld (HH) smartphone, where ML, AP, and IS represent the medio–lateral, antero–posterior, and inferior–superior axes respectively. The error bars represent confidence intervals.

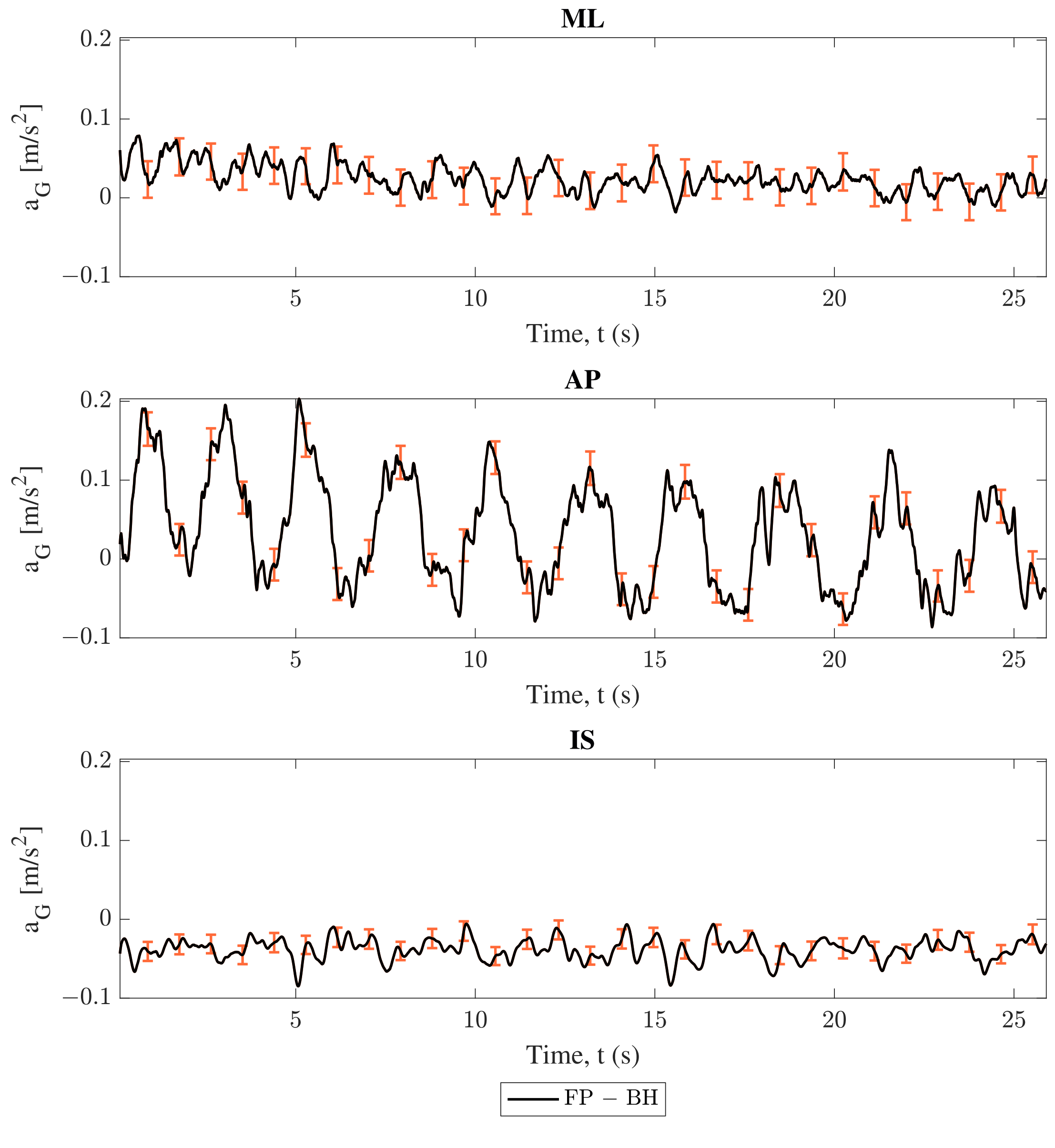

Figure S10: Trial 3 center of mass acceleration (a_G) difference: force plate (FP) subtracted from back harness (BH) smartphone, where ML, AP, and IS represent the medio–lateral, antero–posterior, and inferior–superior axes respectively. The error bars represent confidence intervals.

Center of Pressure Comparison

Center of pressure projections from smartphones were compared to force plate data. The comparison between these results for Trial 3 (swaying in antero–posterior direction) are plotted in Figures S11 and S12, where force plate results (FP) are compared to the handheld (HH) and back harness (BH) smartphones, respectively.

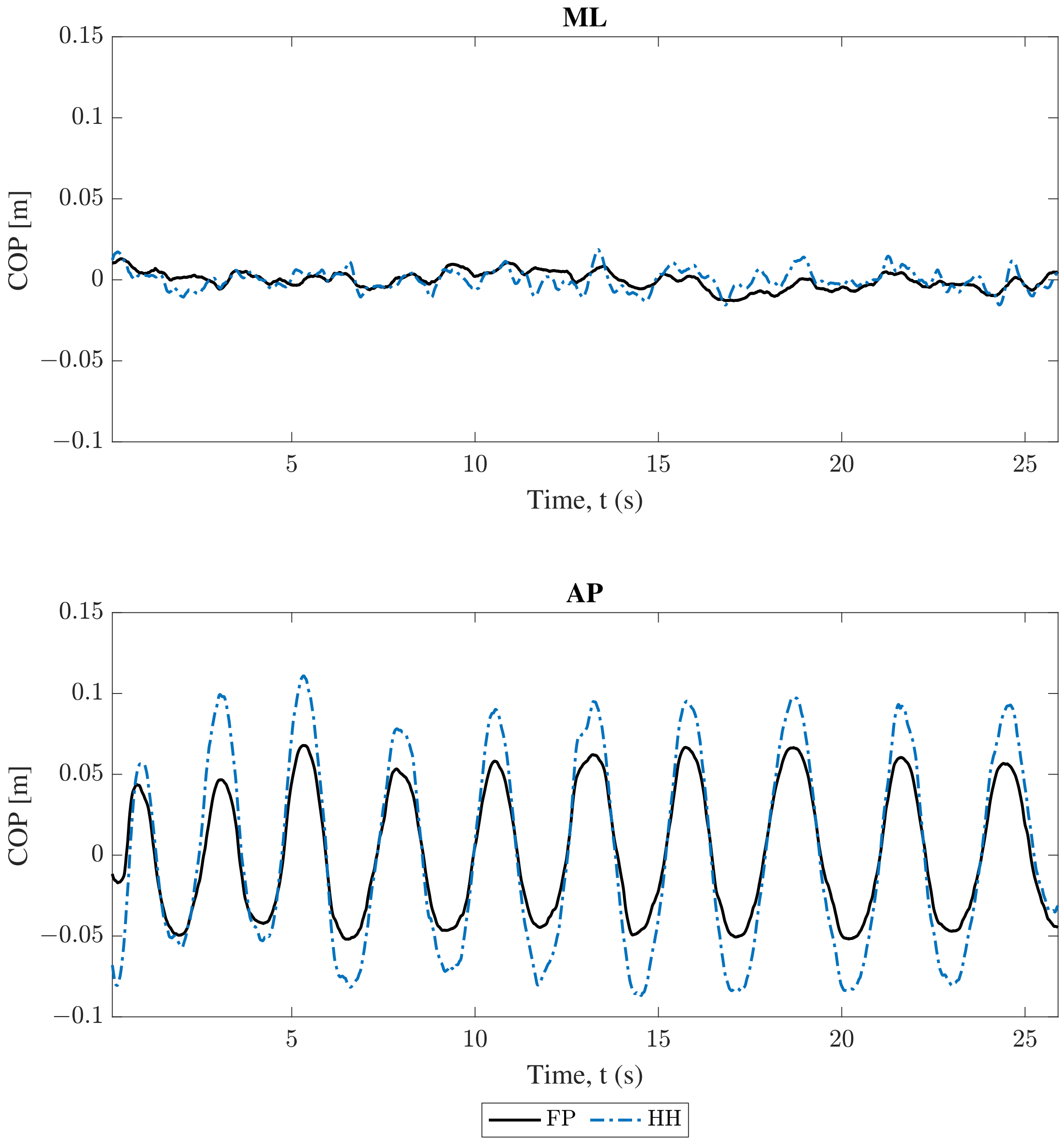

Figure S11: Trial 3 COP position comparison. Force plate (FP) versus handheld (HH) smartphone, where ML and AP represent the medio–lateral and antero–posterior axes, respectively.

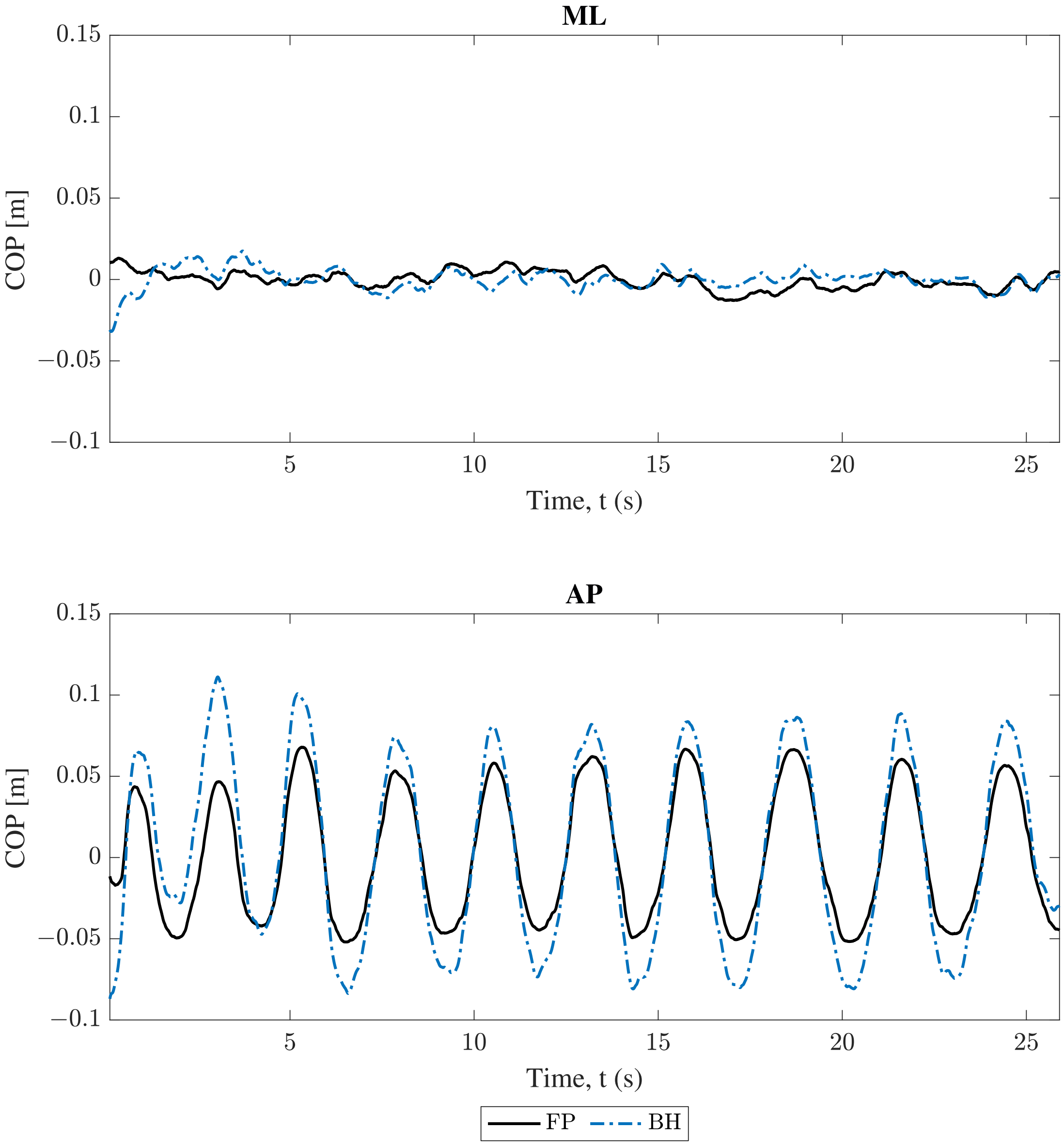

Figure S12: Trial 3 COP position comparison. Force plate (FP) versus back harness (BH) smartphone, where ML and AP represent the medio–lateral and antero–posterior axes, respectively.

1.3 Trial 4

Center of Mass Acceleration Comparison

As described in Section 2 of the paper, COM acceleration estimations were compared between each phone and the force plate. The comparison between these results for Trial 3 (random swaying in both directions) are shown in Figures S13 – S16. Force plate results (FP) are compared to the handheld (HH) and back harness (BH) smartphones in Figures S13 and S14, respectively. The differences of results plotted in Figures S13 and S14 are shown in Figures S15 and S16, respectively.

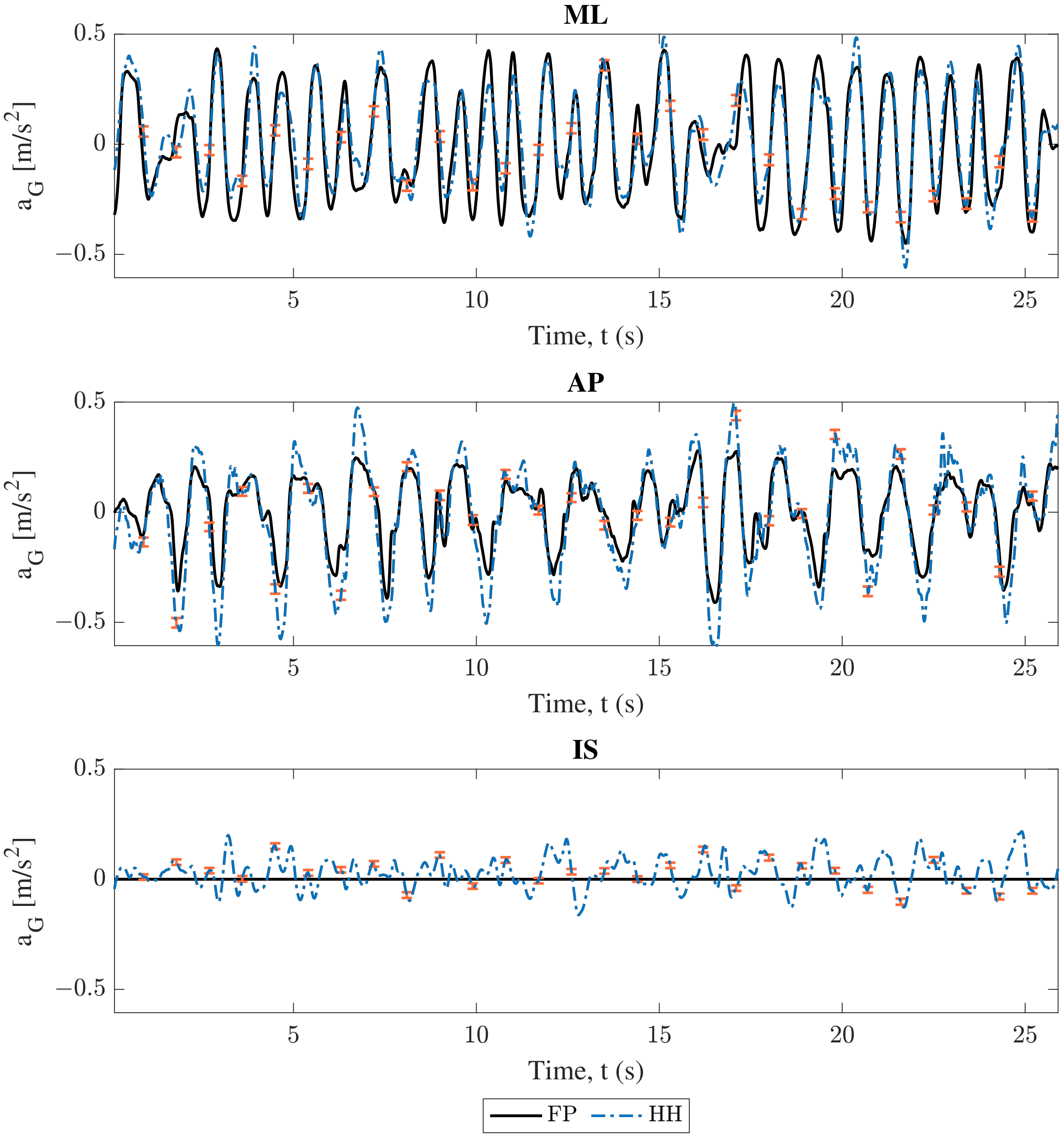

Figure S13: Trial 4 center of mass acceleration (a_G) comparison. Force plate (FP) versus handheld smartphone (HH), where ML, AP, and IS represent the medio–lateral, antero–posterior, and inferior–superior axes respectively. The error bars represent confidence intervals.

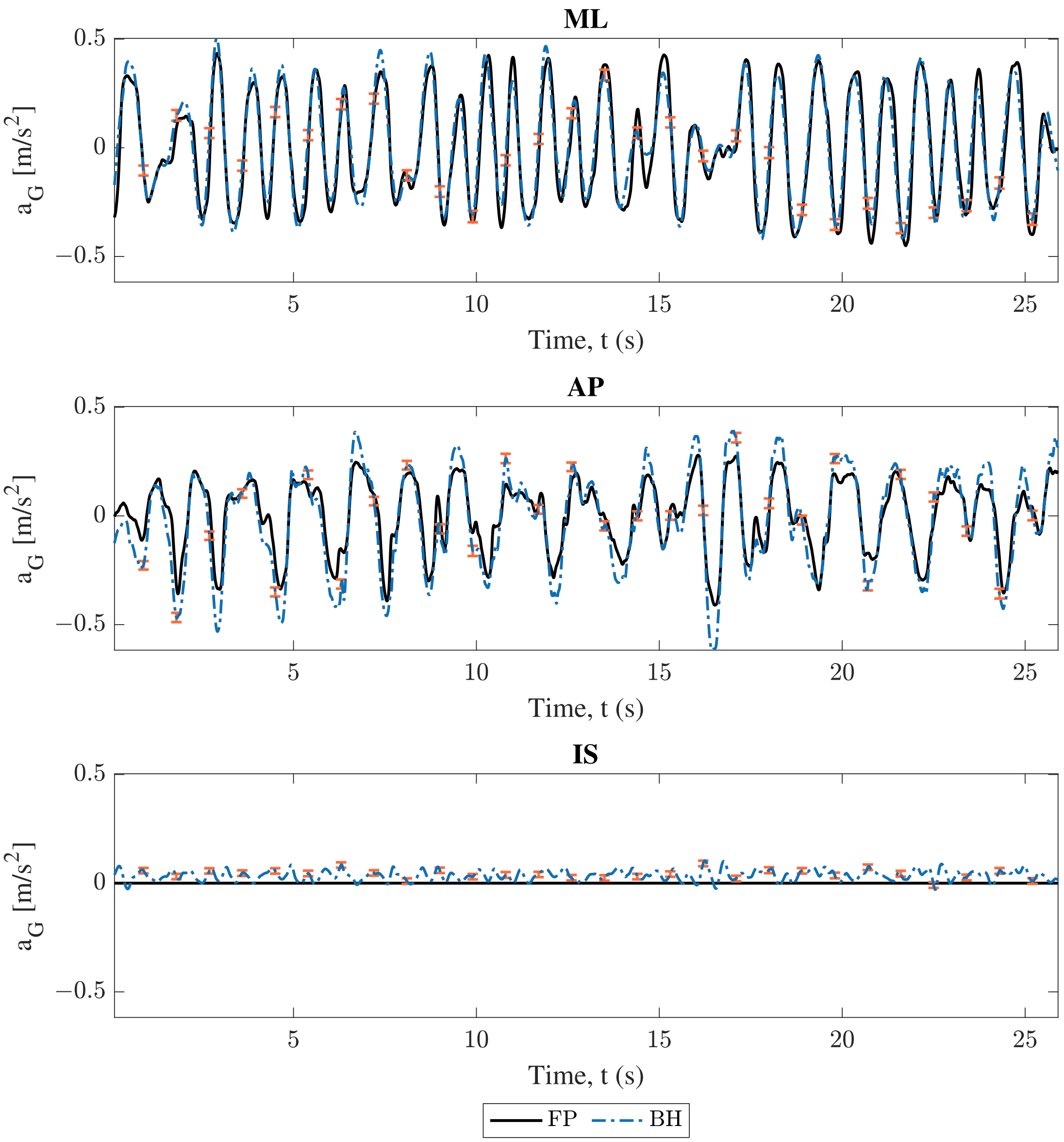

Figure S14: Trial 4 center of mass acceleration (a_G) comparison. Force plate (FP) versus back harness smartphone (BH), where ML, AP, and IS represent the medio–lateral, antero–posterior, and inferior–superior axes respectively. The error bars represent confidence intervals.

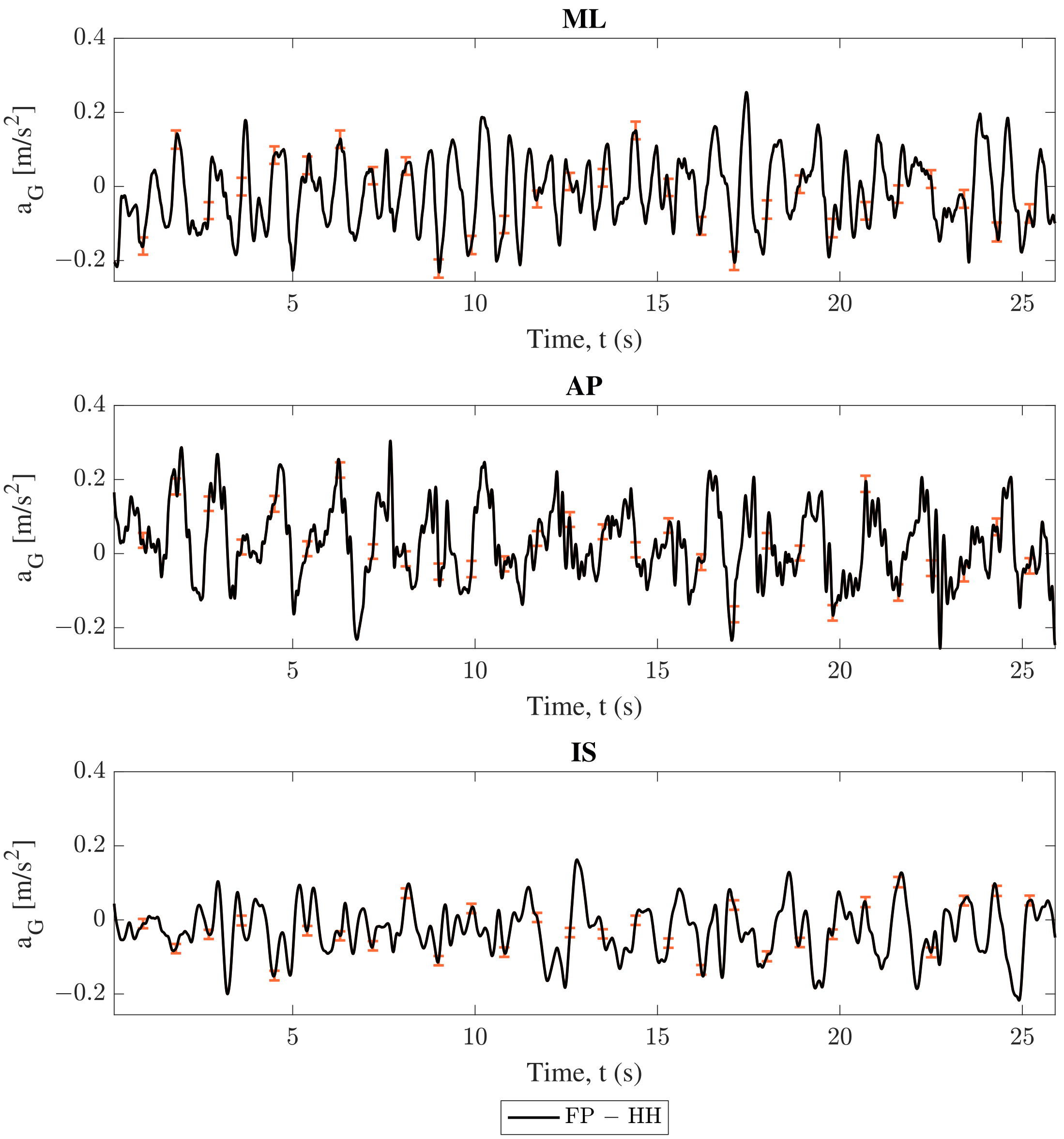

Figure S15: Trial 4 center of mass acceleration (a_G) difference: force plate (FP) subtracted from handheld (HH) smartphone, where ML, AP, and IS represent the medio–lateral, antero–posterior, and inferior–superior axes respectively. The error bars represent confidence intervals.

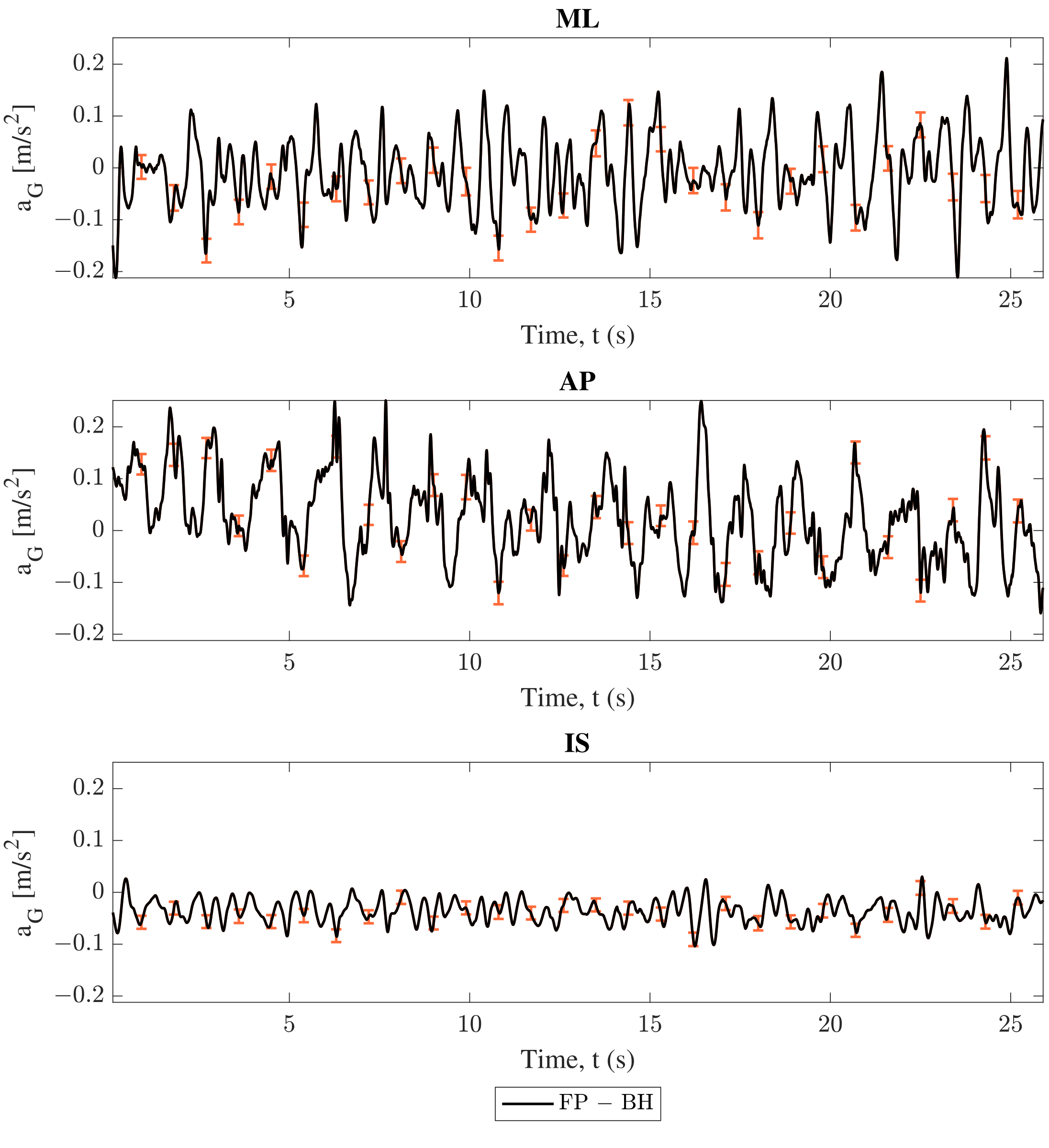

Figure S16: Trial 4 center of mass acceleration (a_G) difference: force plate (FP) subtracted from back harness (BH) smartphone, where ML, AP, and IS represent the medio–lateral, antero–posterior, and inferior–superior axes respectively. The error bars represent confidence intervals.

Center of Pressure Comparison

Center of pressure projections from smartphones were compared to force plate data. The comparison between these results for Trial 3 (random swaying in both directions) are plotted in Figures S17 and S18, where force plate results (FP) are compared to the handheld (HH) and back harness (BH) smartphones, respectively.

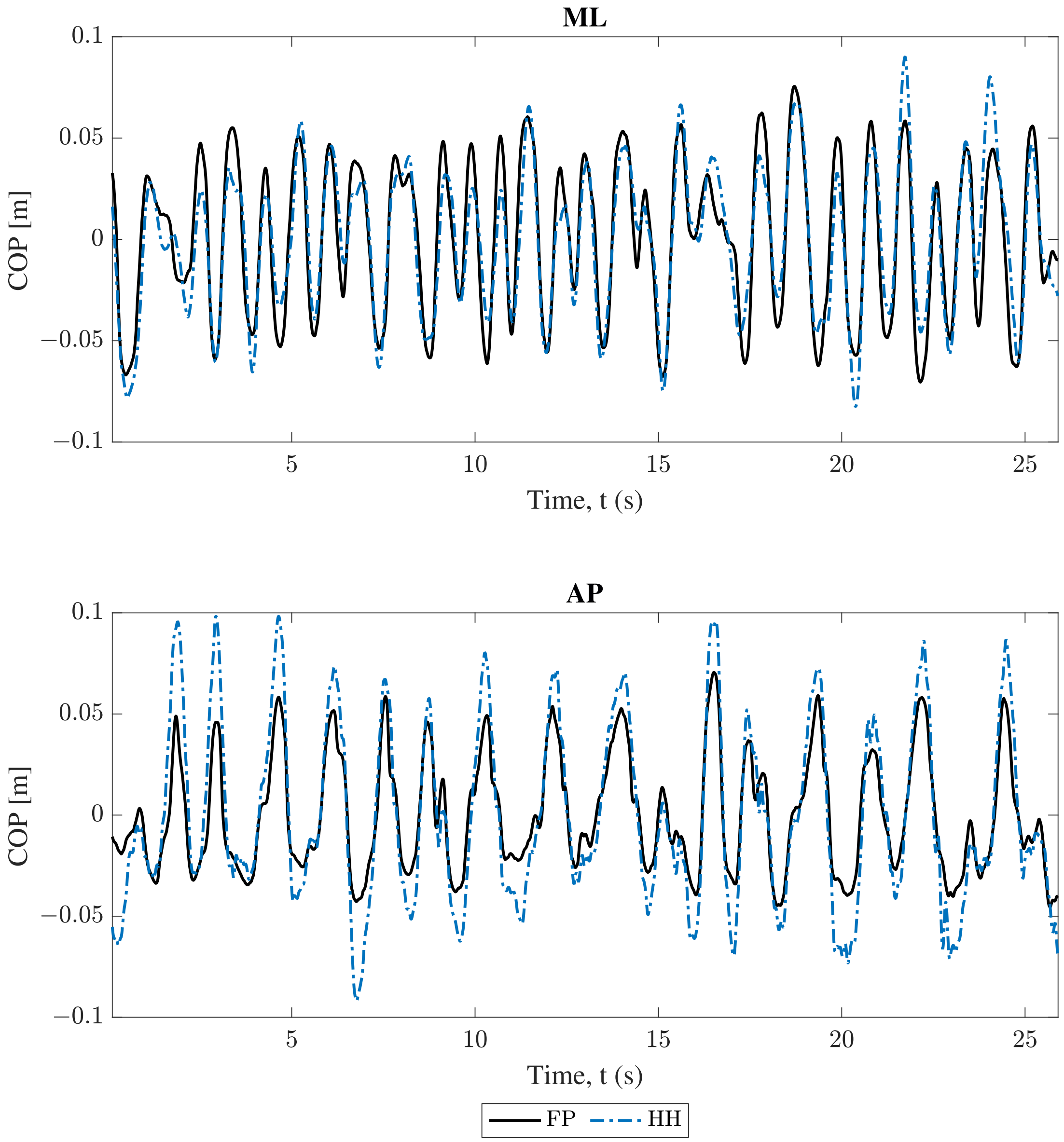

Figure S17: Trial 4 COP position comparison. Force plate (FP) versus handheld (HH) smartphone, where ML and AP represent the medio–lateral and antero–posterior axes, respectively.

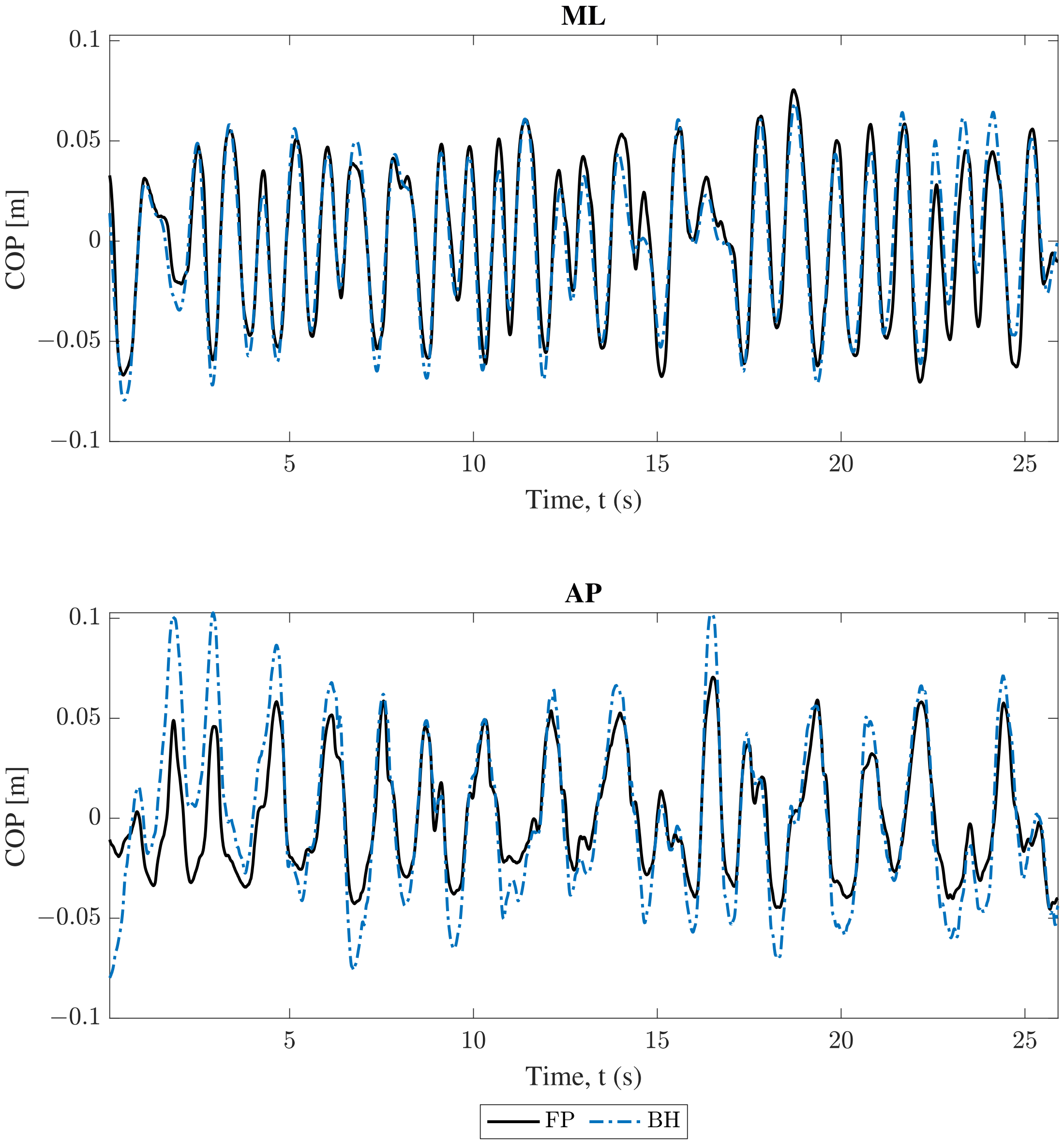

Figure S18: Trial 4 COP position comparison. Force plate (FP) versus back harness (BH) smartphone, where ML and AP represent the medio–lateral and antero–posterior axes, respectively.
