# Supplementary material for: Validation of Smartphones in Arbitrary Positions Against Force Plate Standard for Balance Assessment
Source: Sensors (Basel). 2025 Apr 22;25(9):2639. doi: 10.3390/s25092639 (PMC12073742; doi:10.3390/s25092639)
Supplement: Supplementary file 1 [file sensors-25-02639-s001.zip › Validation of Mobile Devices w Force Plates for Balance Assessment/FinalEdits.pdf]

Manuscript ID: sensors-3529895
Title: Validation of Smartphones in Arbitrary Positions Against Force Plate Standard For Balance Assessment
Authors: German Jack Ellsworth, Stephen M. Klisch, Britta Berg-Johansen, and Eric Ocegueda

Author's Final Proofreading

Here we provide a list of modifications we made with changes marked in red in the manuscript. Additionally, the * refers to items that addressed highlighted comments from the production team.

1. ORCID added for authors.

2.* Paragraph 3 of Introduction: we removed the italics from "posturography".

3. Paragraph 4 of Introduction: corrected wrong tense "were" → "was". Improved clarity by adding author's name to statement.

4. Paragraph 5 of Introduction: minor changes to wording.

5.* Paragraph 1 of Section 2.1: added city to devices listed.

6.* Table 1 caption: added Supplementary Figure numbers.

7. Paragraph 2 of Section 2.1: clarified wording at the start of paragraph ("had a duration of" → "lasted") and replaced "s" with "seconds".

8.* Section 2.2: Added company cities and version number for MATLAB Mobile app.

9. Section 2.3.2: Added abbreviations ML and AP during first appearance in section.

10. End of Section 2.8: Added mention of computing root square mean error along with Spearman's rank correlation.

11. Section 3: Fixed typo "participating" → "participant" and added Supplementary Figure numbers.

12. Figures 4–9: Replaced hyphen with minus sign in all occurrences.

13.* Section 4.4.1: Removed italics for "small".

14. Section 4.4.2: Added clarification that filtering was only applied to smartphone data.

15.* Section 4.5: Added version and company location.

16. Section 5: Added statement to address the Academic Editor's comment.

17.* Reference 33: added date reference was accessed.

18.* Figure captions added to Supplementary Materials Section.

19.* Institutional Review: we mentioned that review was waived from our institution since the participants were the researchers.

20. Throughout manuscript: for consistency added "–" between medio–lateral and antero-posterior at all occurances.
