# Supplementary figures and images for: Validation of Smartphones in Arbitrary Positions Against Force Plate Standard for Balance Assessment
Source: Sensors (Basel). 2025 Apr 22;25(9):2639. doi: 10.3390/s25092639 (PMC12073742; doi:10.3390/s25092639)

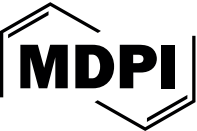

Supplement: Supplementary file 1 [file sensors-25-02639-s001.zip › Validation of Mobile Devices w Force Plates for Balance Assessment/Definitions/logo-mdpi-eps-converted-to.pdf]

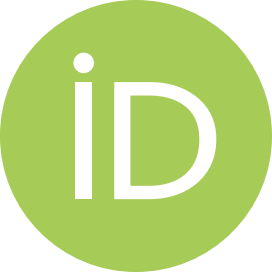

Supplement: Supplementary file 1 [file sensors-25-02639-s001.zip › Validation of Mobile Devices w Force Plates for Balance Assessment/Definitions/logo-orcid.pdf]

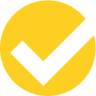

check for  
updates

Supplement: Supplementary file 1 [file sensors-25-02639-s001.zip › Validation of Mobile Devices w Force Plates for Balance Assessment/Definitions/logo-updates-eps-converted-to.pdf]

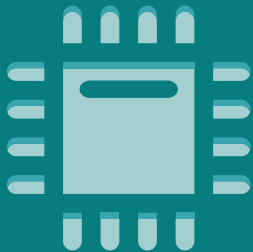

*sensors*

Supplement: Supplementary file 1 [file sensors-25-02639-s001.zip › Validation of Mobile Devices w Force Plates for Balance Assessment/Definitions/sensors-logo-eps-converted-to.pdf]

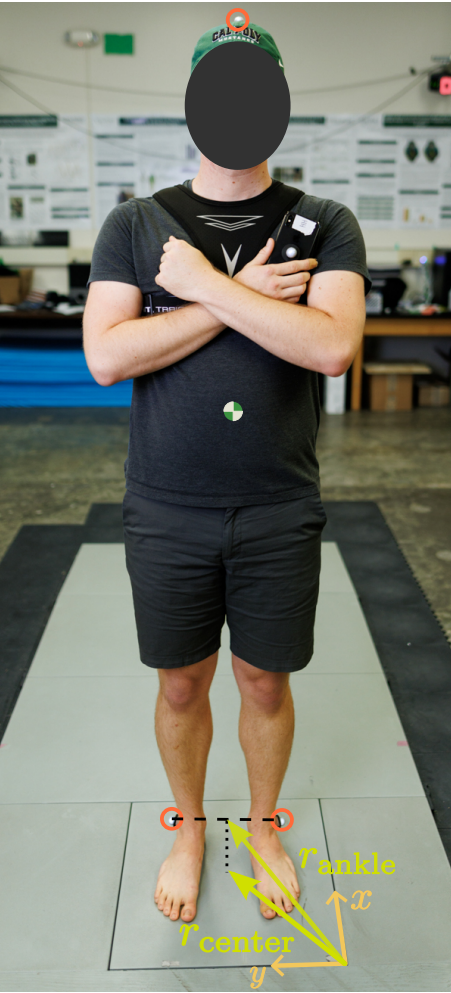

(a)

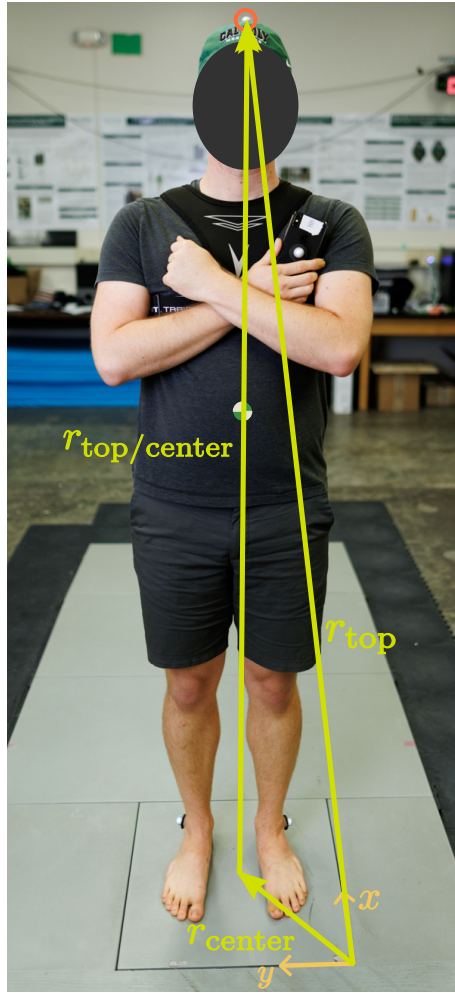

(b)

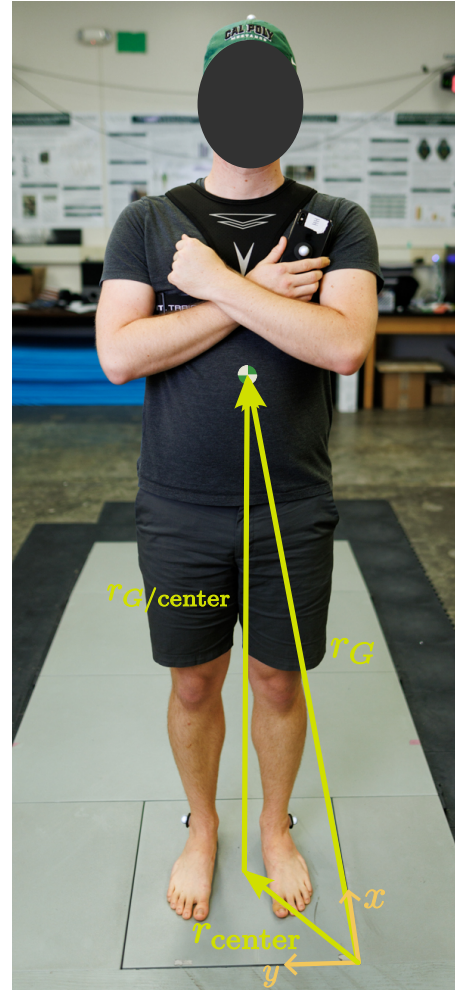

(c)

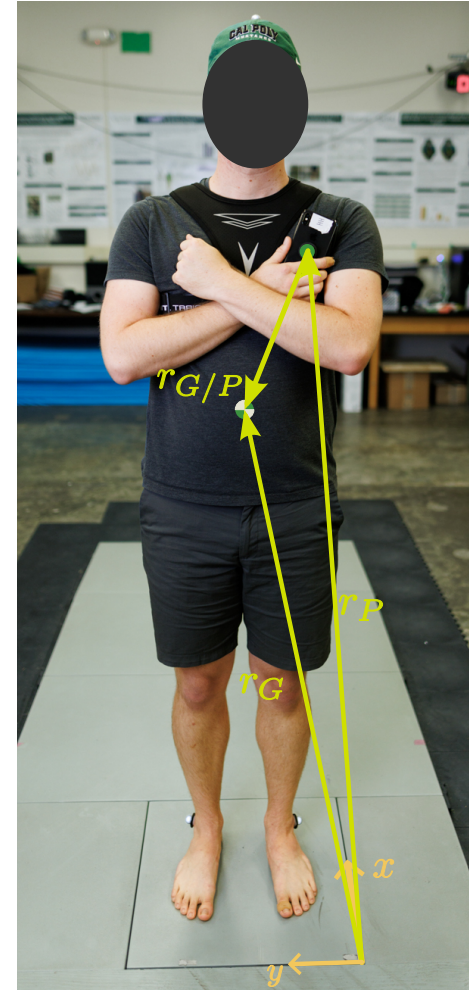

(d)

Supplement: Supplementary file 1 [file sensors-25-02639-s001.zip › Validation of Mobile Devices w Force Plates for Balance Assessment/figures/COM_Vector_Construct_2.pdf]

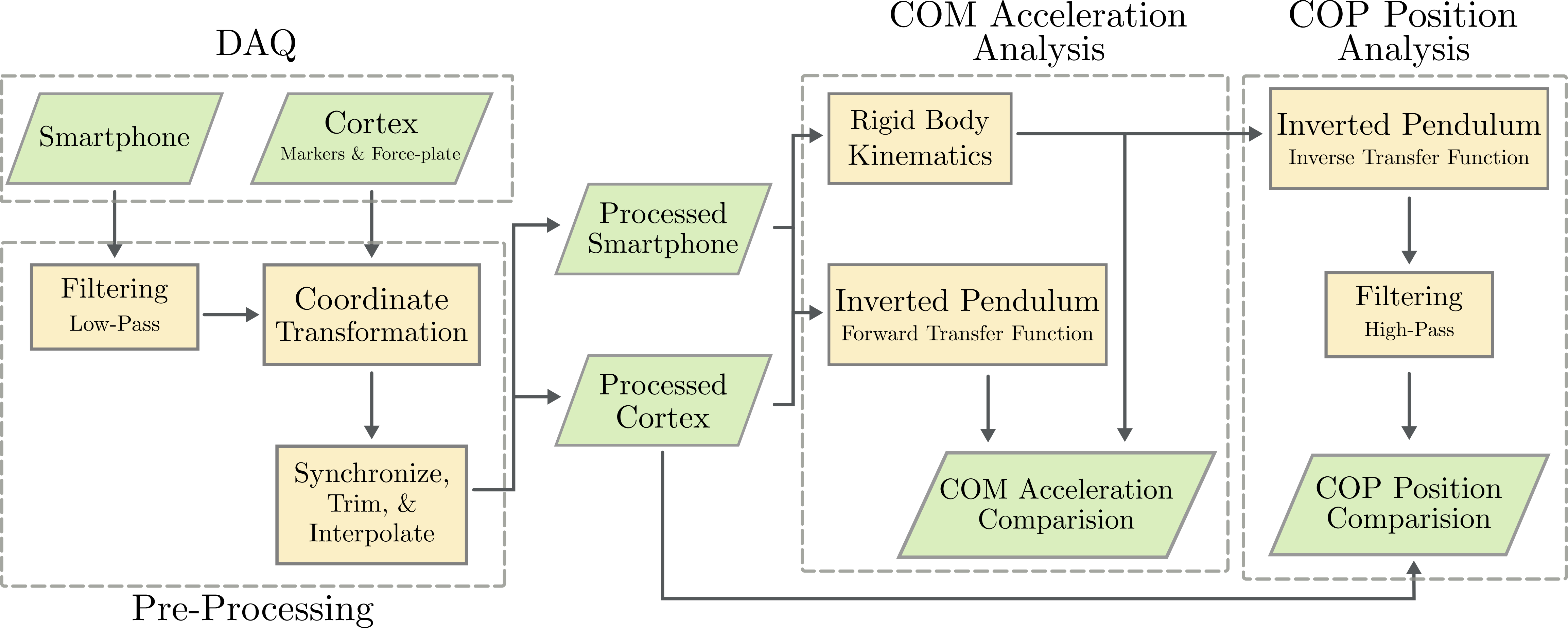

Supplement: Supplementary file 1 [file sensors-25-02639-s001.zip › Validation of Mobile Devices w Force Plates for Balance Assessment/figures/FlowChart_2.pdf]

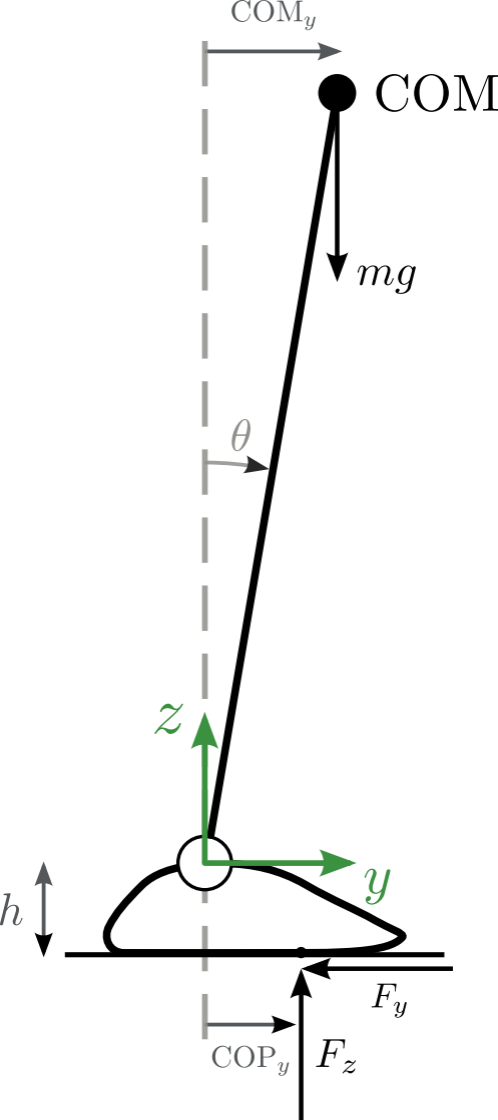

Supplement: Supplementary file 1 [file sensors-25-02639-s001.zip › Validation of Mobile Devices w Force Plates for Balance Assessment/figures/InvertedPendulum.pdf]

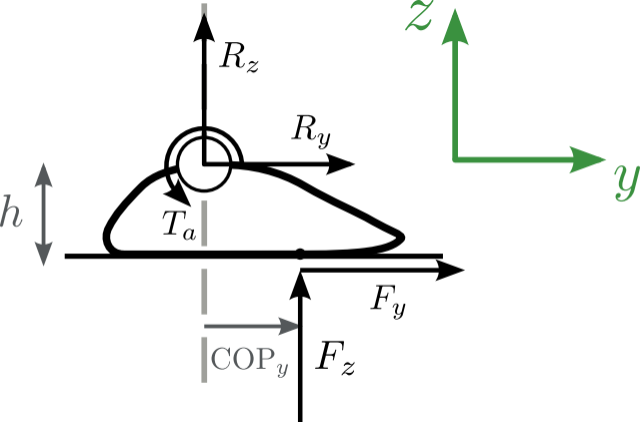

Supplement: Supplementary file 1 [file sensors-25-02639-s001.zip › Validation of Mobile Devices w Force Plates for Balance Assessment/figures/InvertedPendulum_Ankle.pdf]

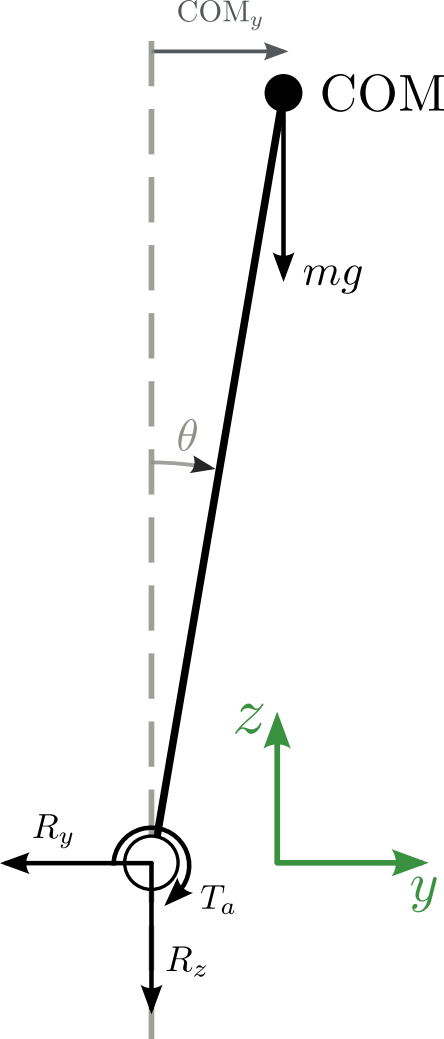

Supplement: Supplementary file 1 [file sensors-25-02639-s001.zip › Validation of Mobile Devices w Force Plates for Balance Assessment/figures/InvertedPendulum_Body.pdf]

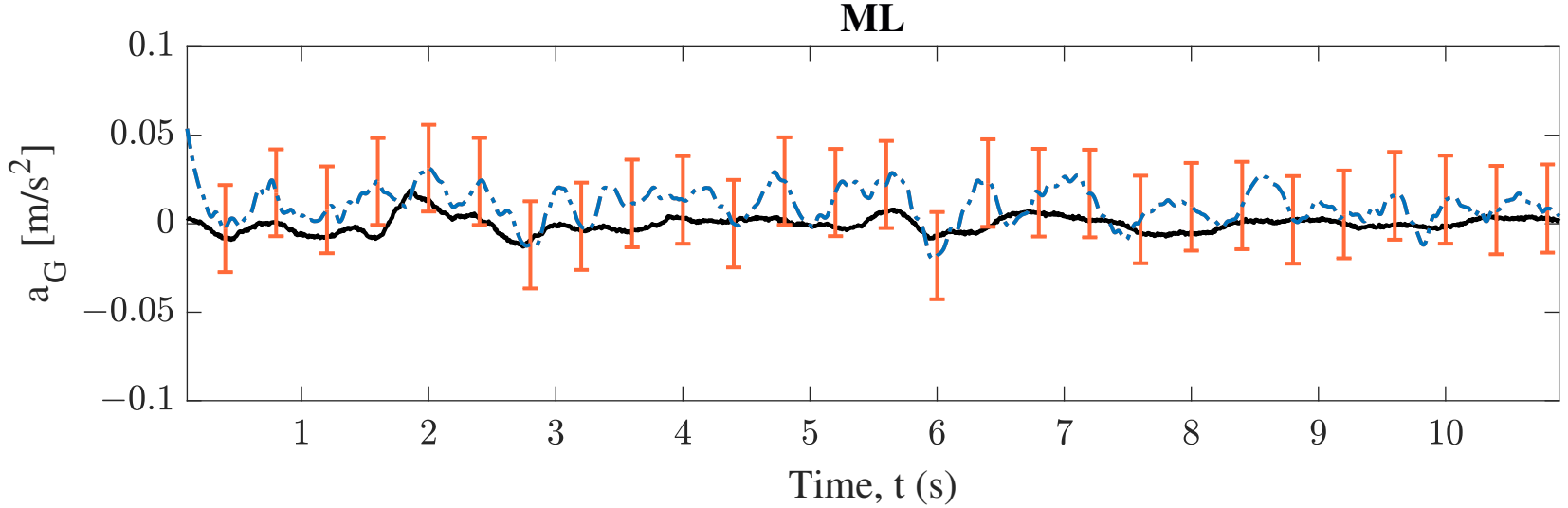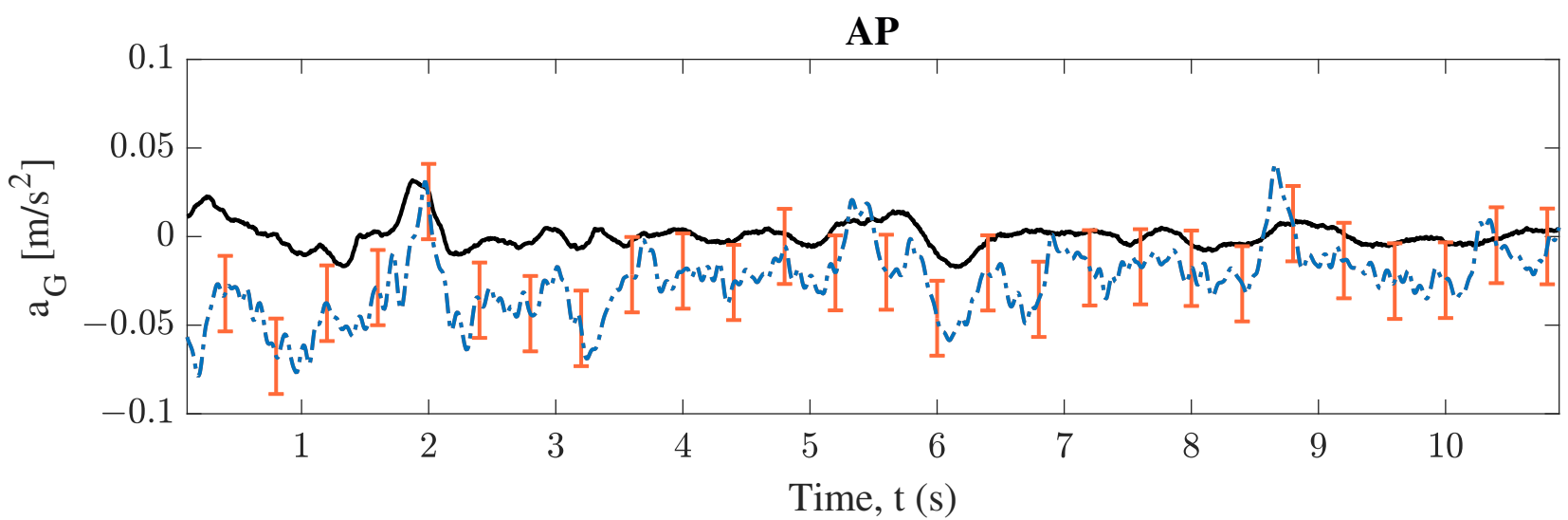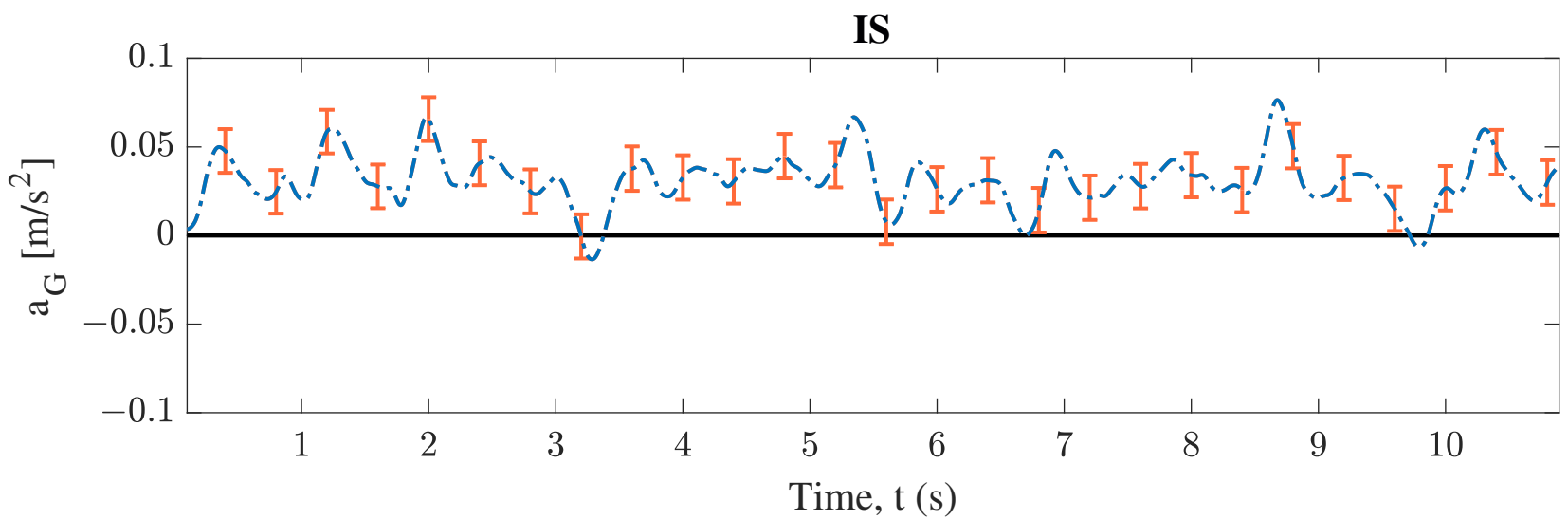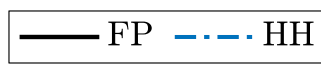

Supplement: Supplementary file 1 [file sensors-25-02639-s001.zip › Validation of Mobile Devices w Force Plates for Balance Assessment/figures/Results/Trial1_Fig1.pdf]

**ML**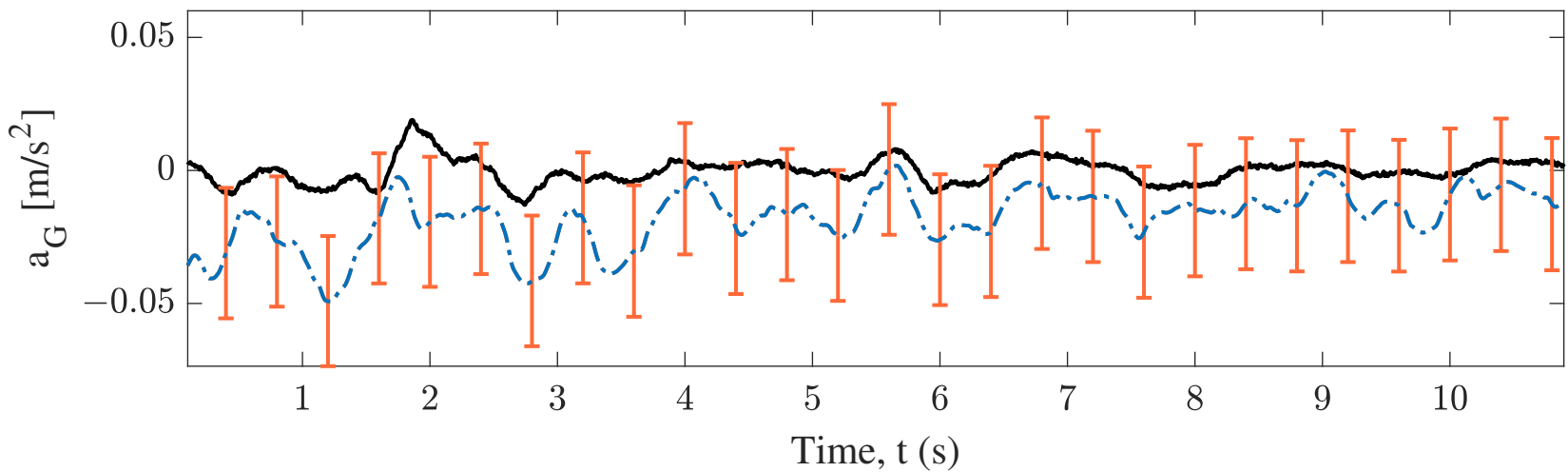**AP**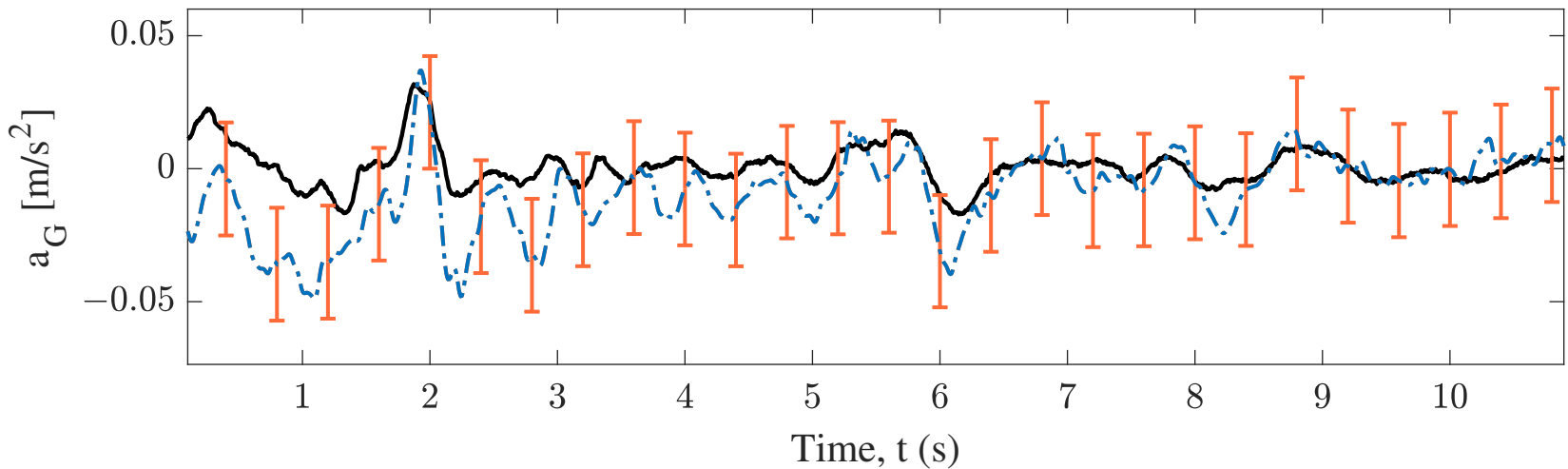**IS**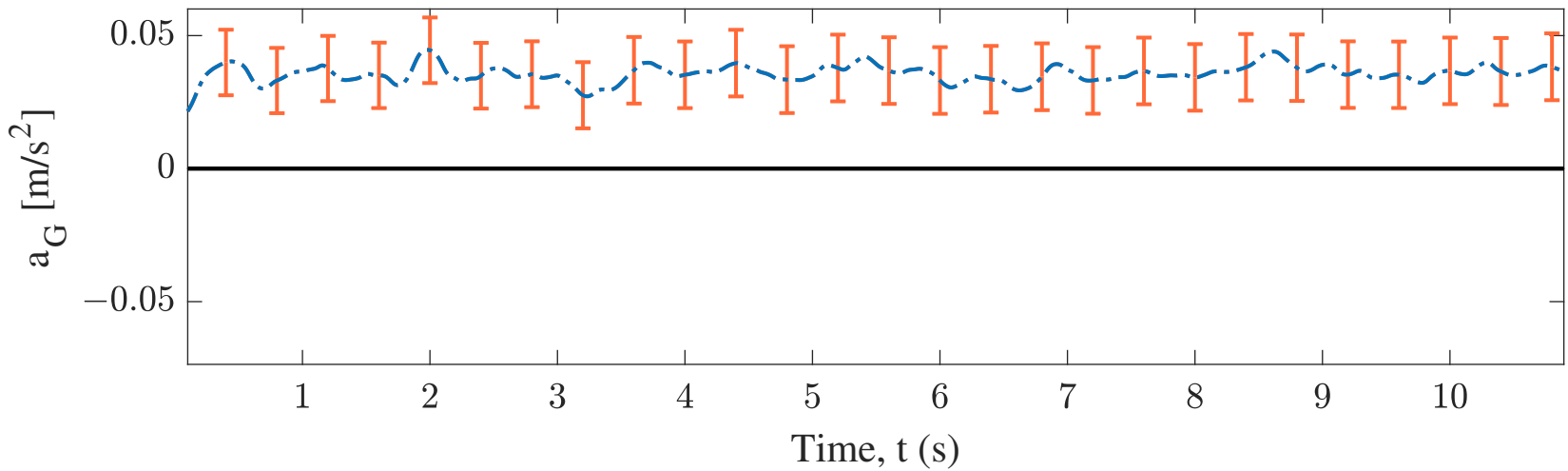

— FP    -.- BH

Supplement: Supplementary file 1 [file sensors-25-02639-s001.zip › Validation of Mobile Devices w Force Plates for Balance Assessment/figures/Results/Trial1_Fig2.pdf]

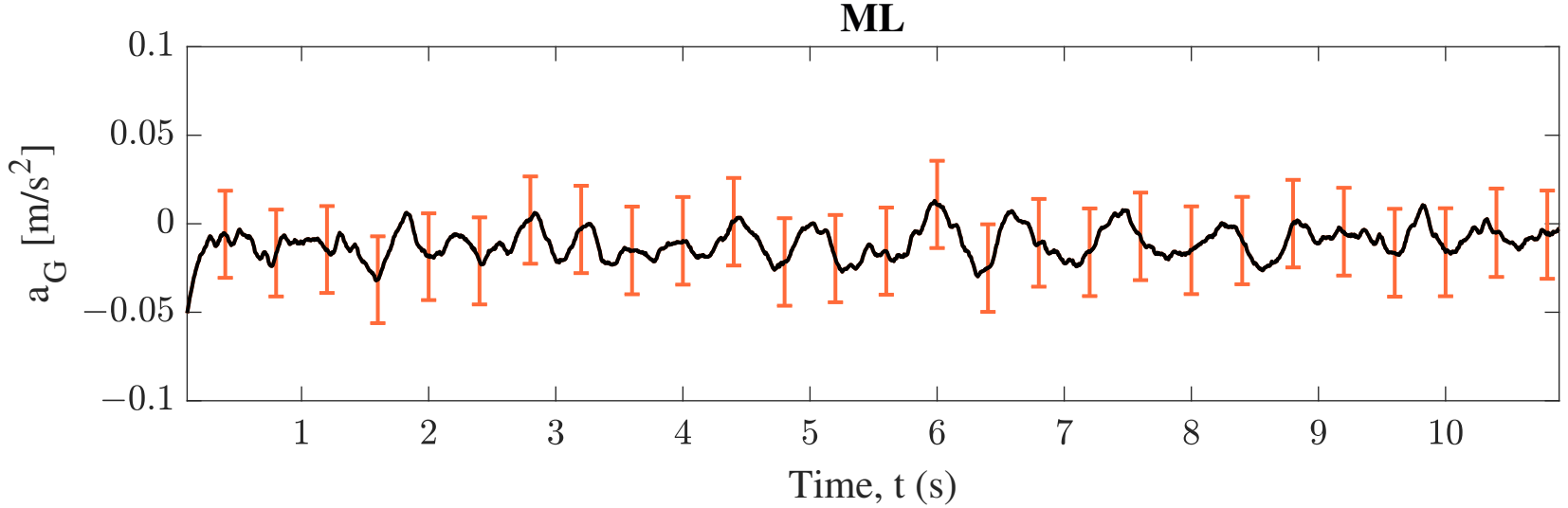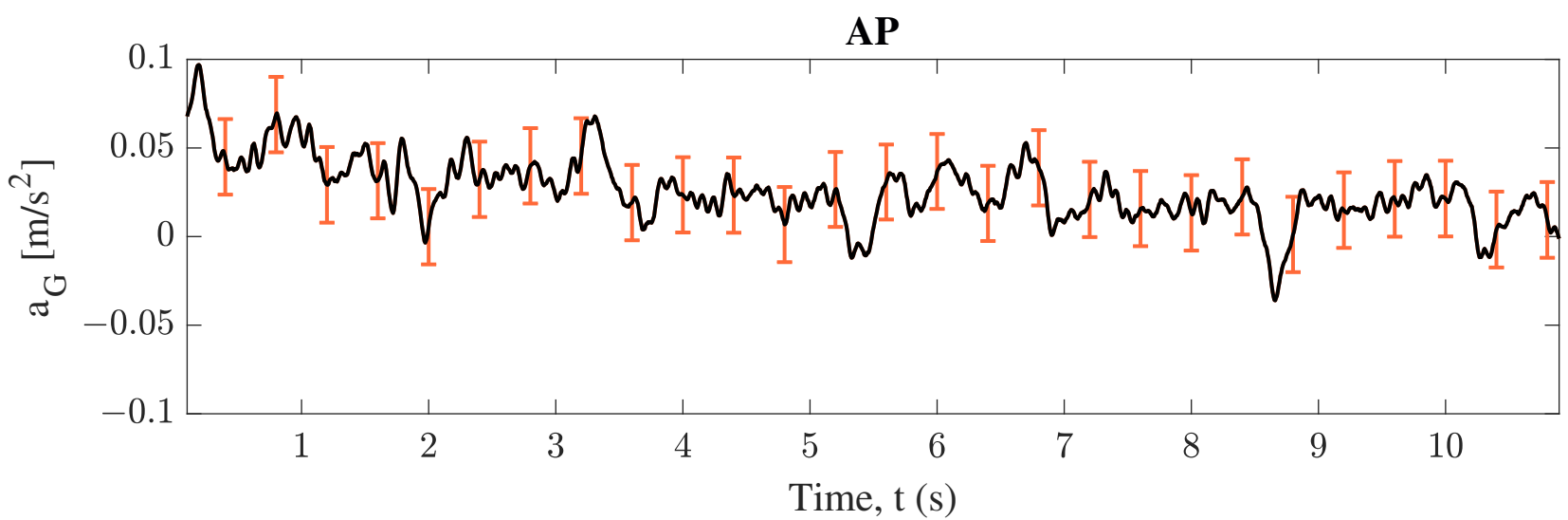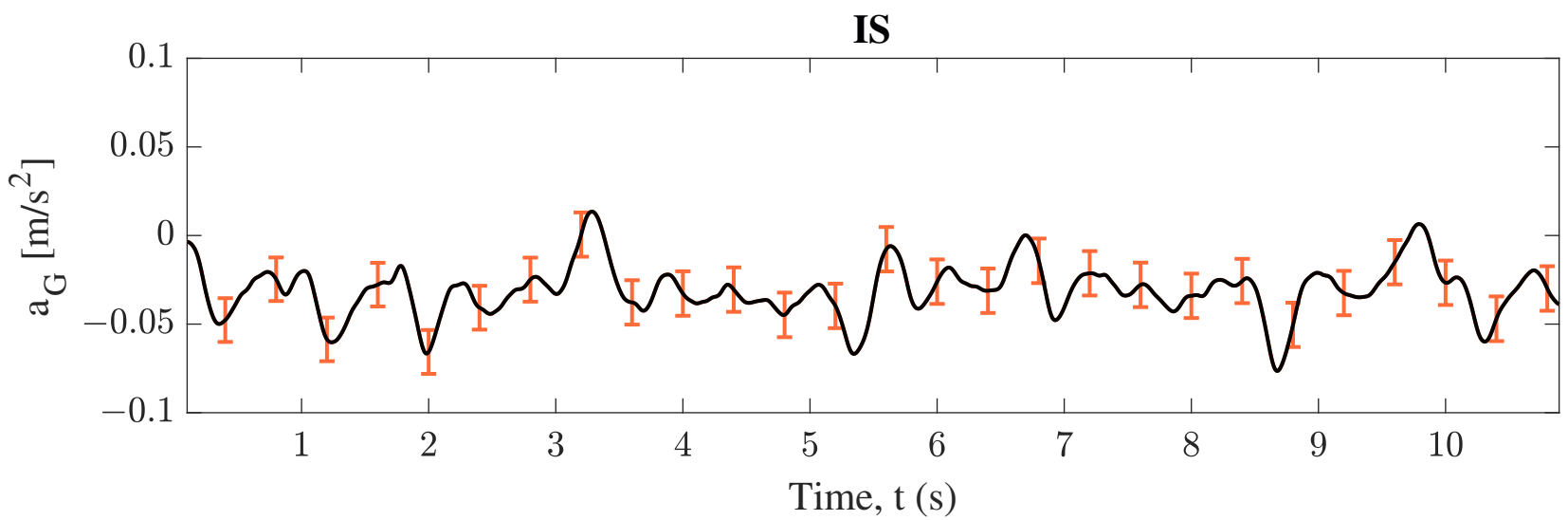

— FP — HH

Supplement: Supplementary file 1 [file sensors-25-02639-s001.zip › Validation of Mobile Devices w Force Plates for Balance Assessment/figures/Results/Trial1_Fig3.pdf]

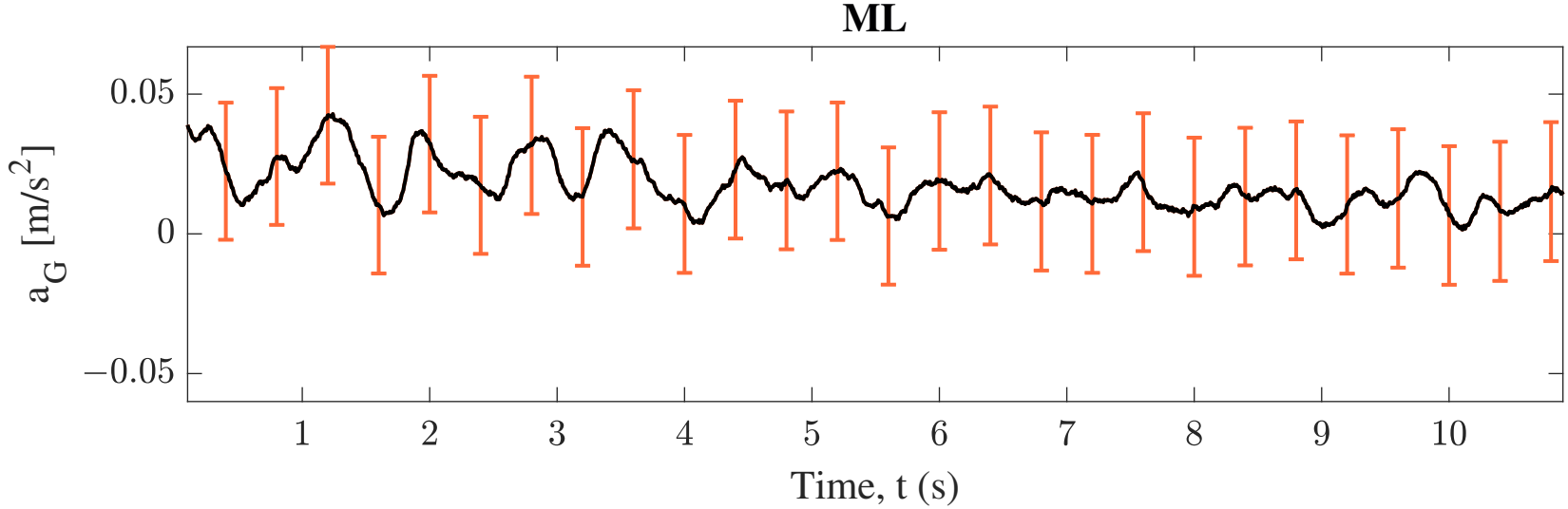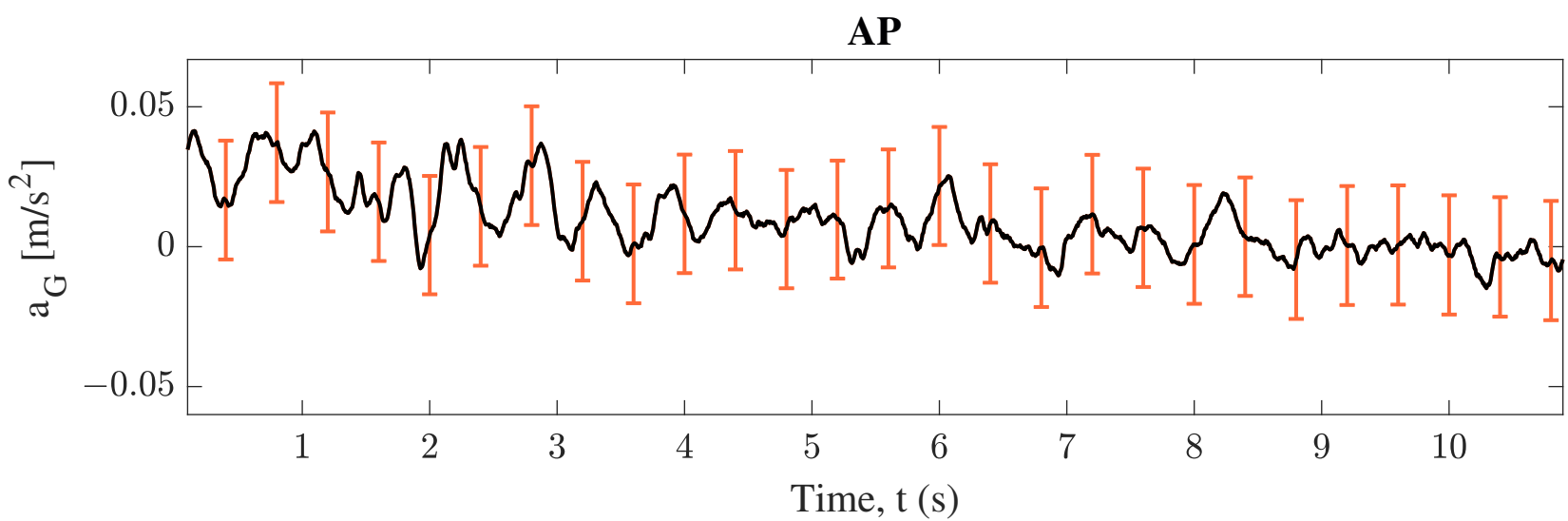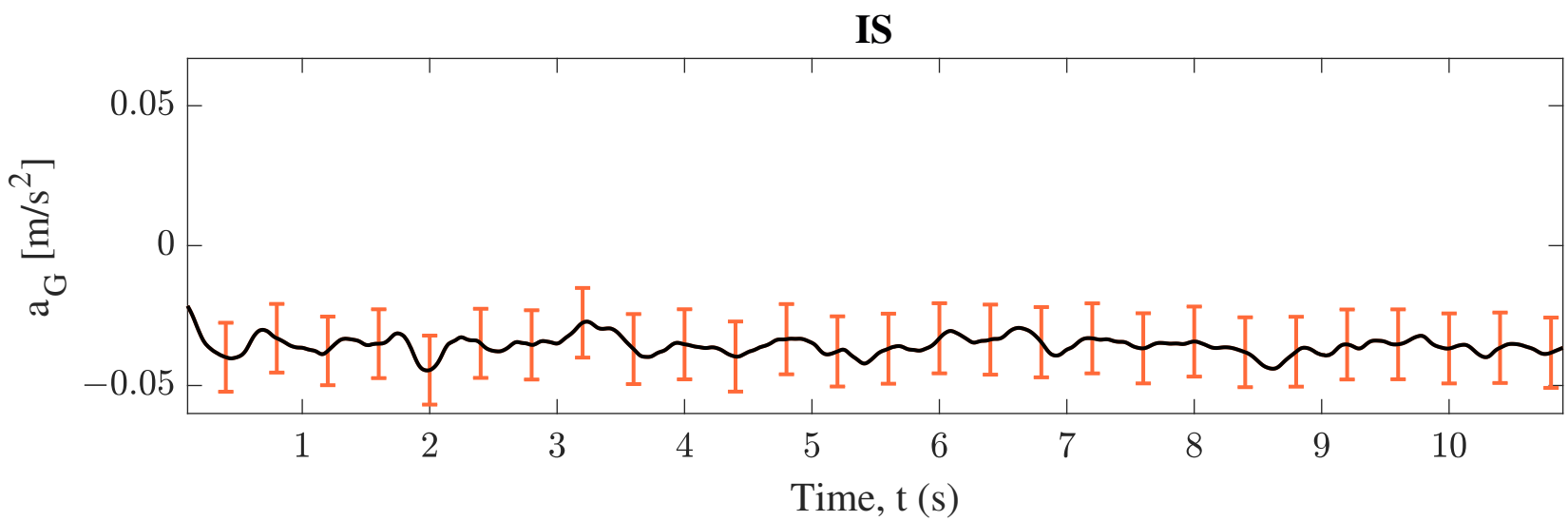

— FP - BH

Supplement: Supplementary file 1 [file sensors-25-02639-s001.zip › Validation of Mobile Devices w Force Plates for Balance Assessment/figures/Results/Trial1_Fig4.pdf]

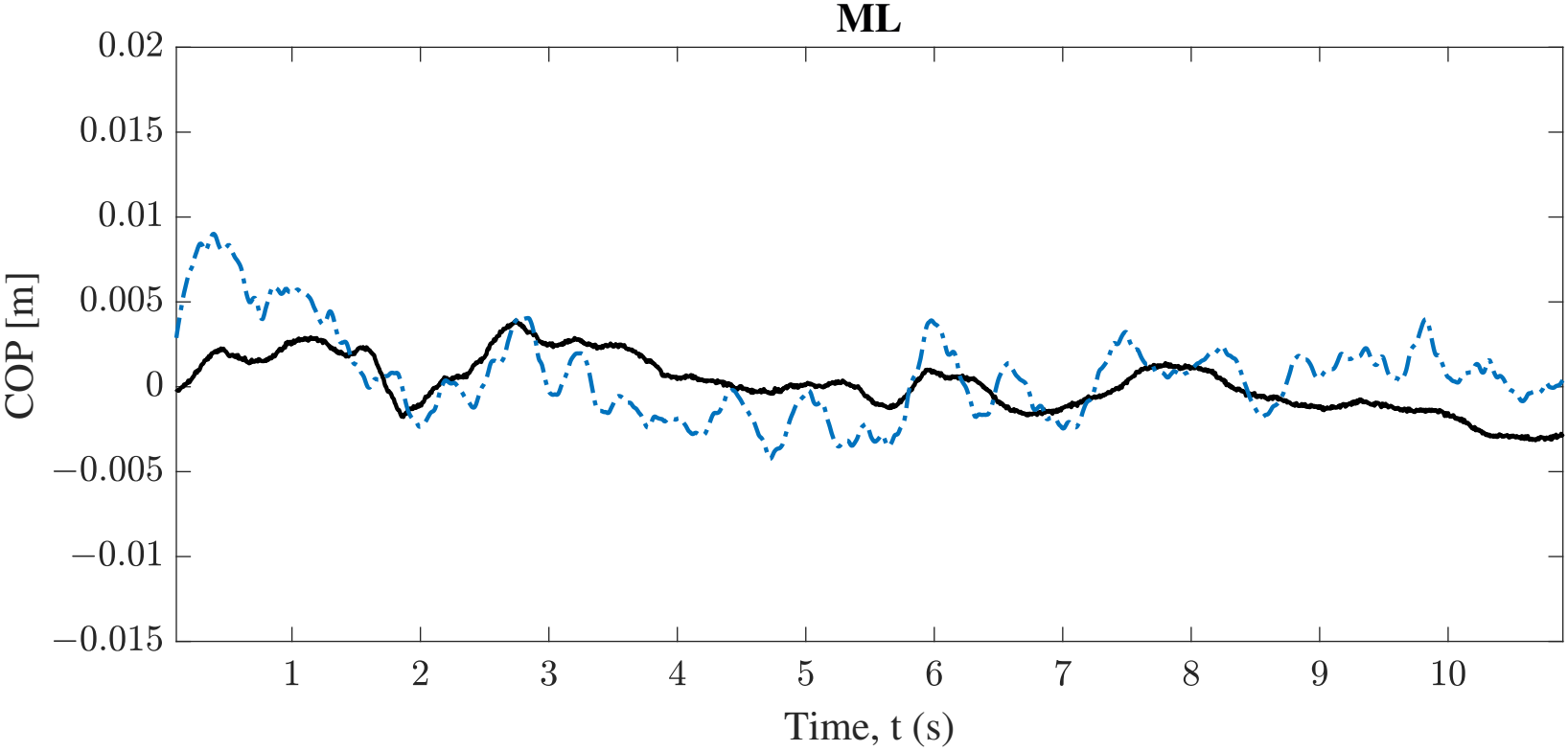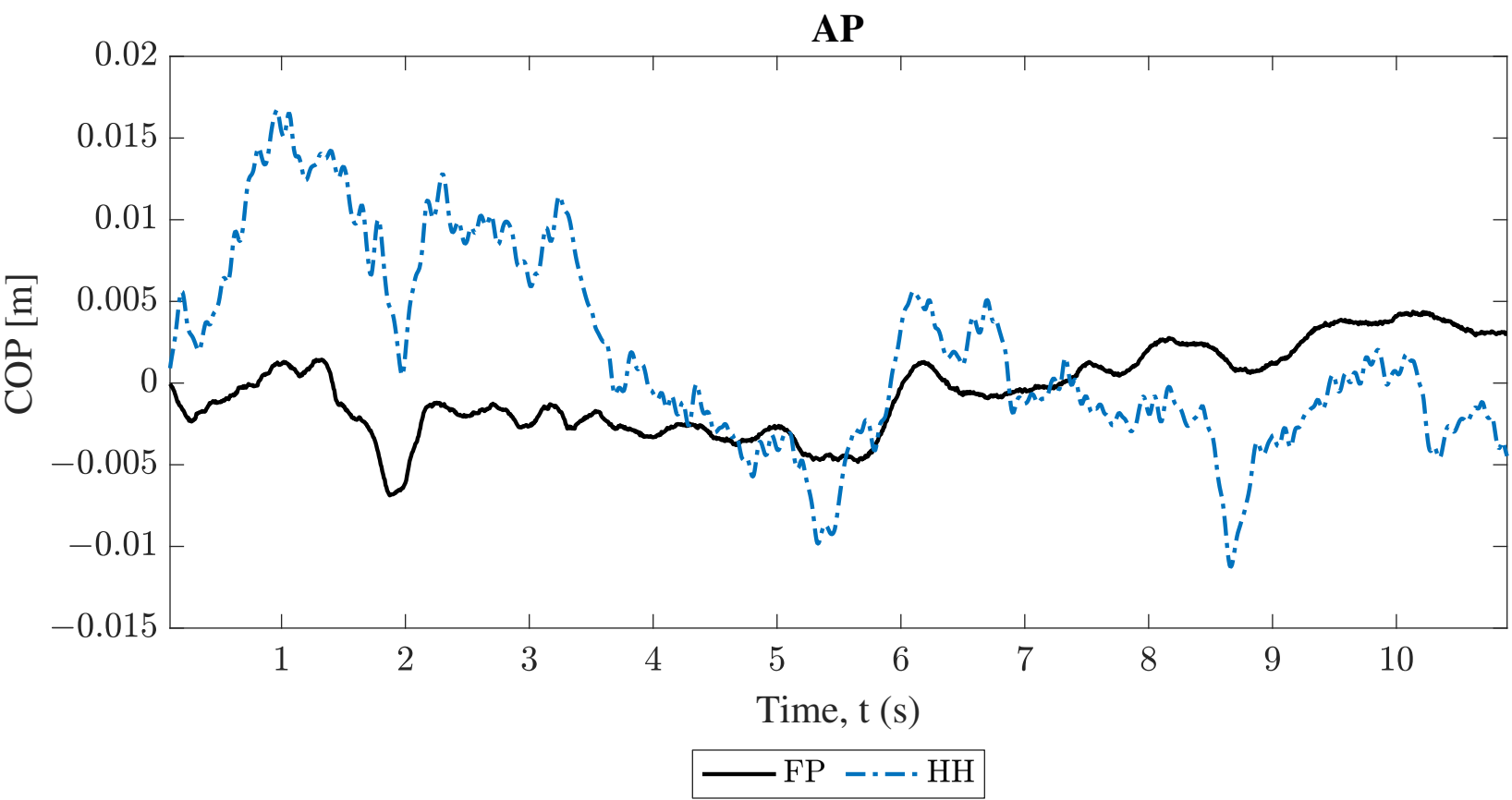

Supplement: Supplementary file 1 [file sensors-25-02639-s001.zip › Validation of Mobile Devices w Force Plates for Balance Assessment/figures/Results/Trial1_Fig5.pdf]

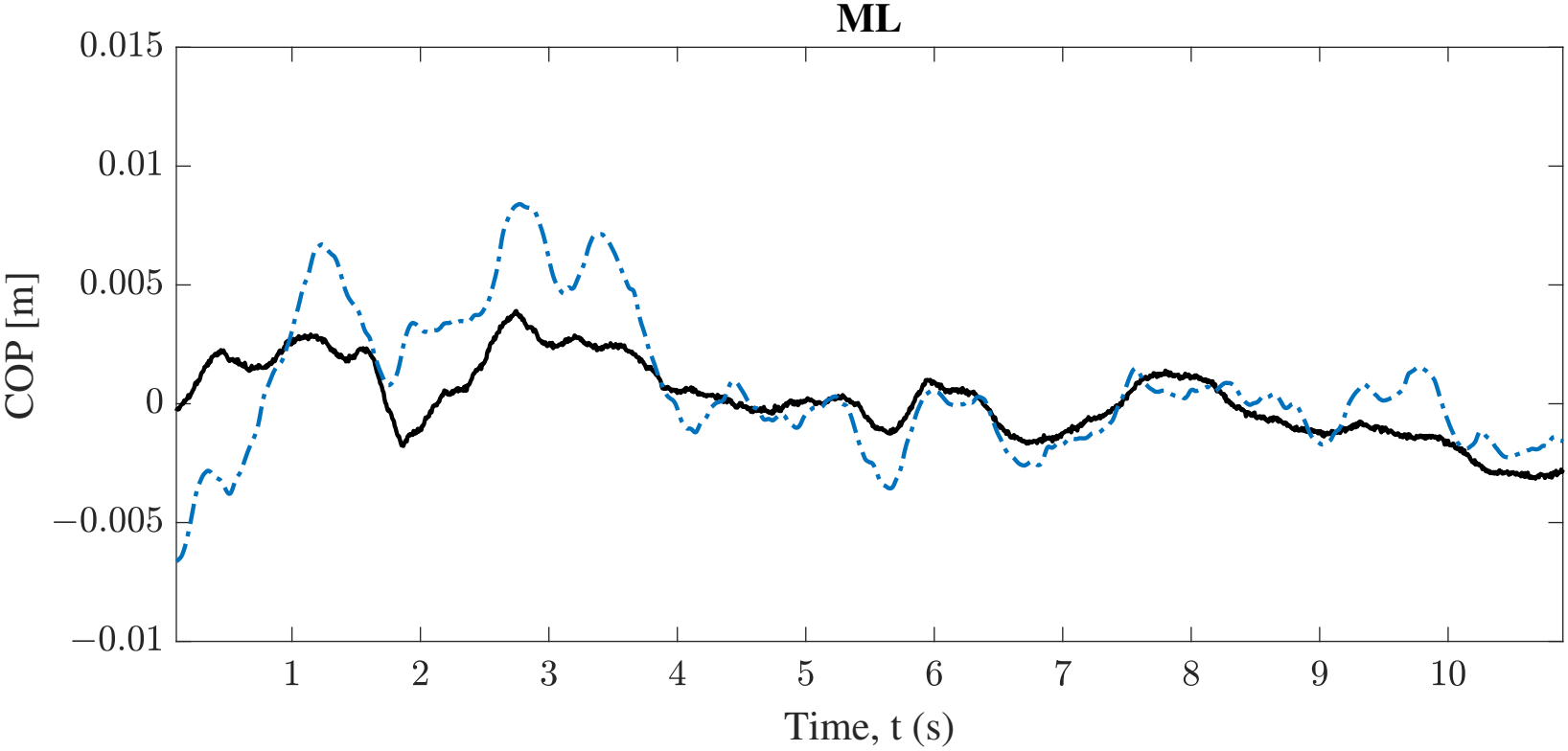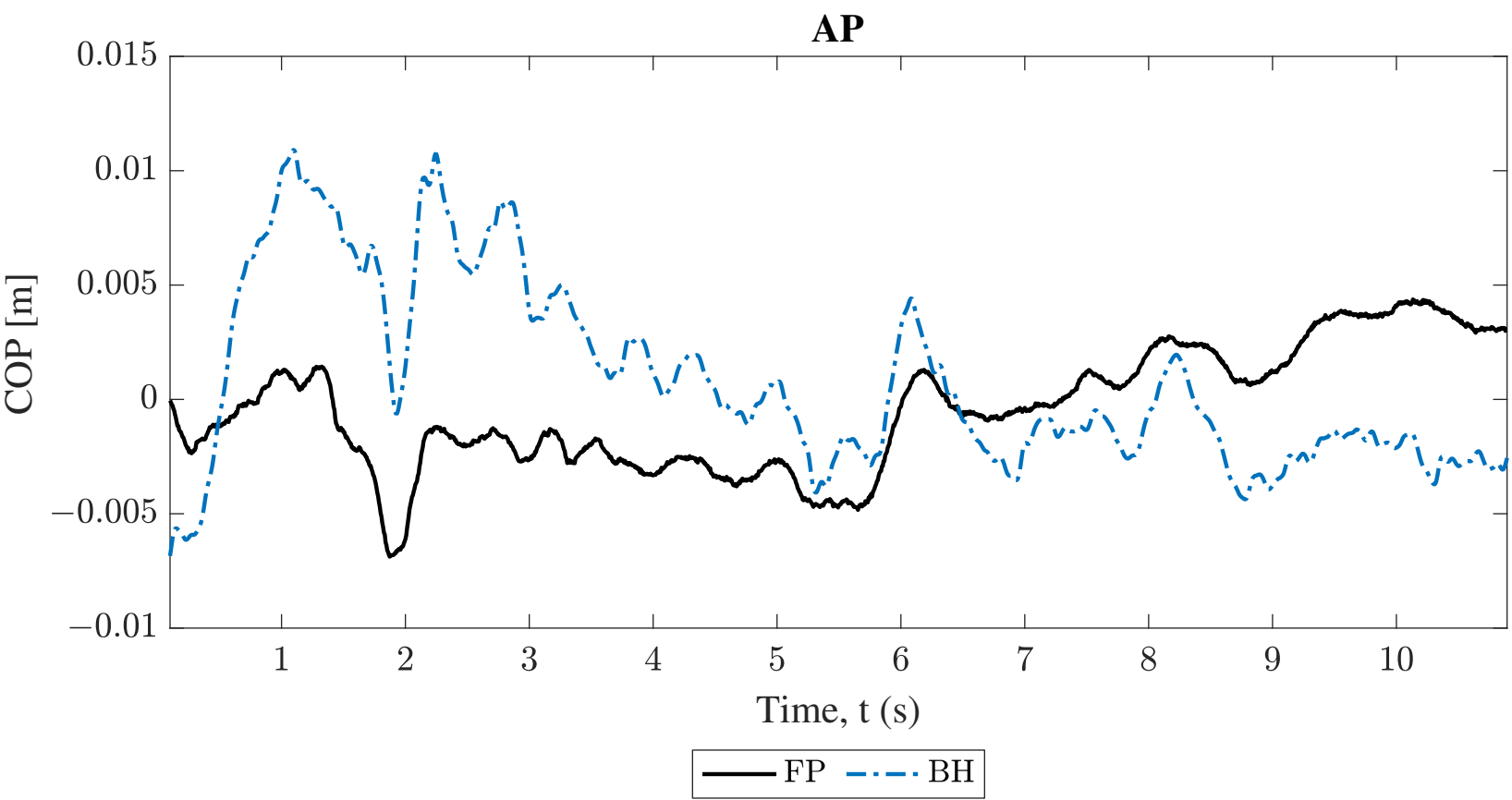

Supplement: Supplementary file 1 [file sensors-25-02639-s001.zip › Validation of Mobile Devices w Force Plates for Balance Assessment/figures/Results/Trial1_Fig6.pdf]

**ML**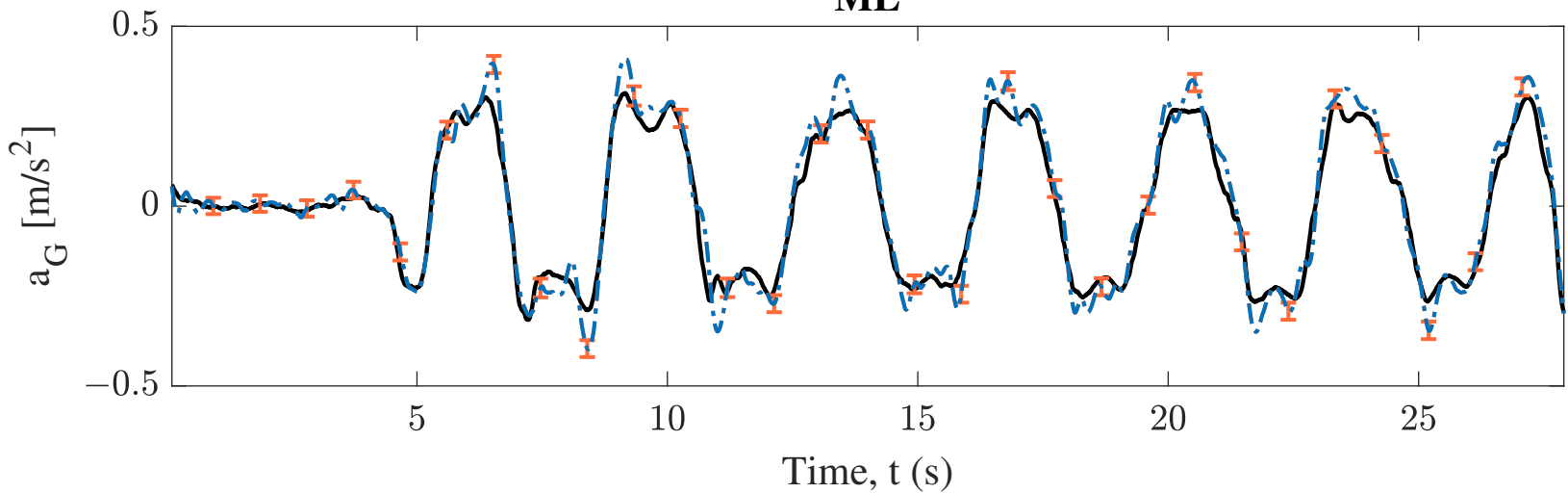**AP**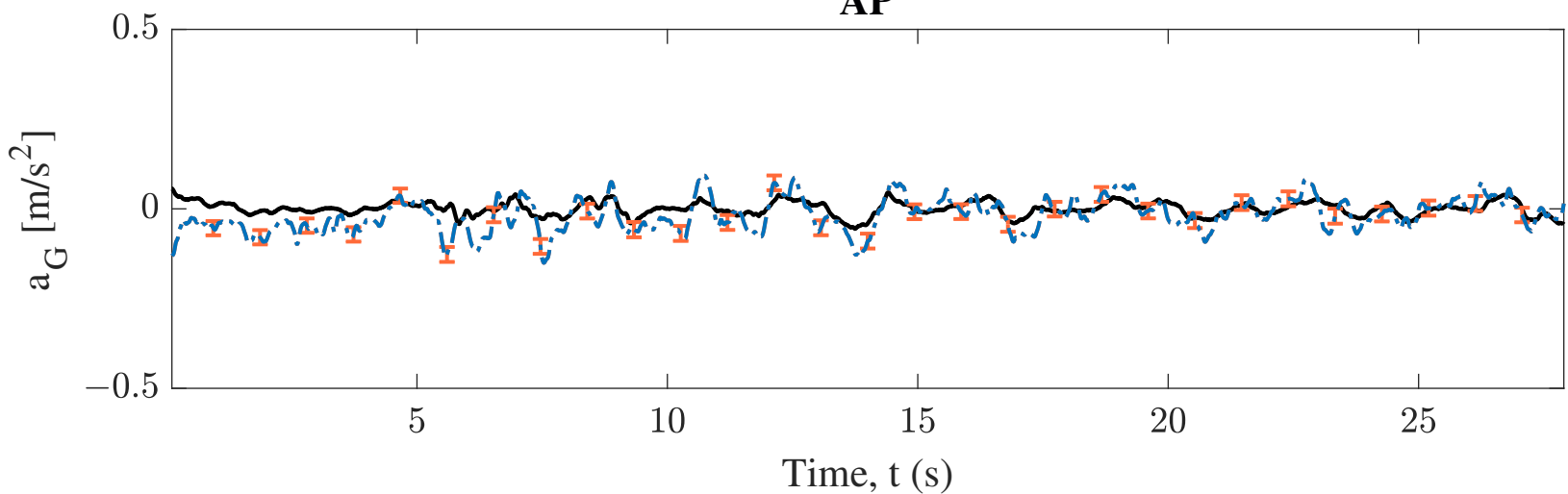**IS**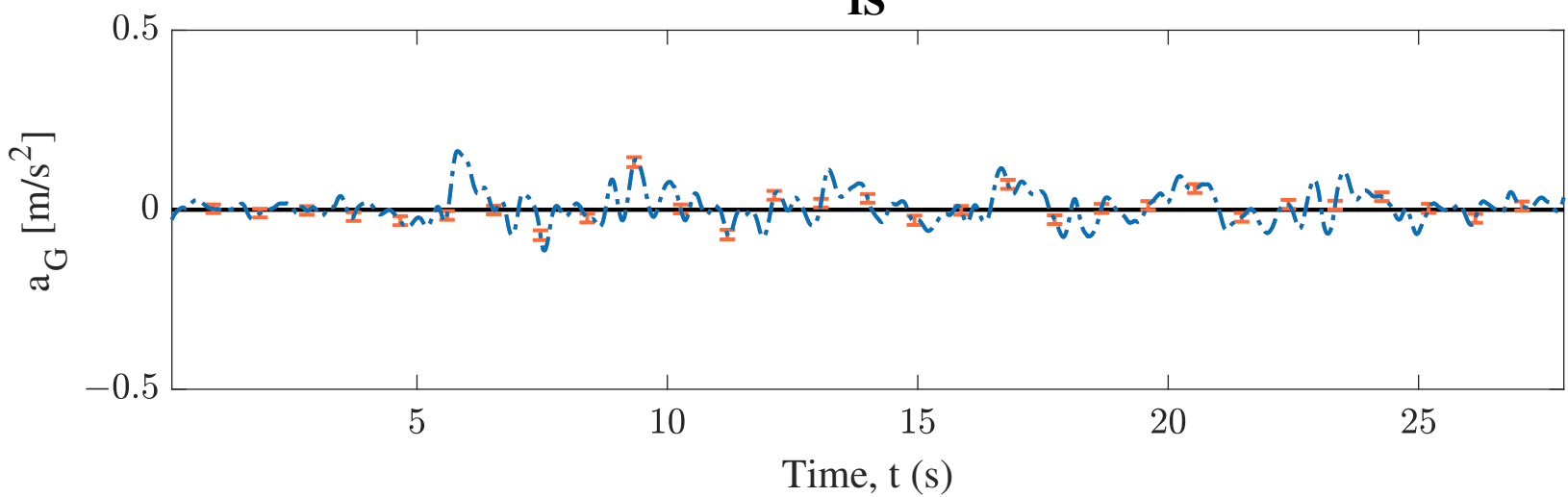

— FP    - - - HH

Supplement: Supplementary file 1 [file sensors-25-02639-s001.zip › Validation of Mobile Devices w Force Plates for Balance Assessment/figures/Results/Trial2_Fig1.pdf]

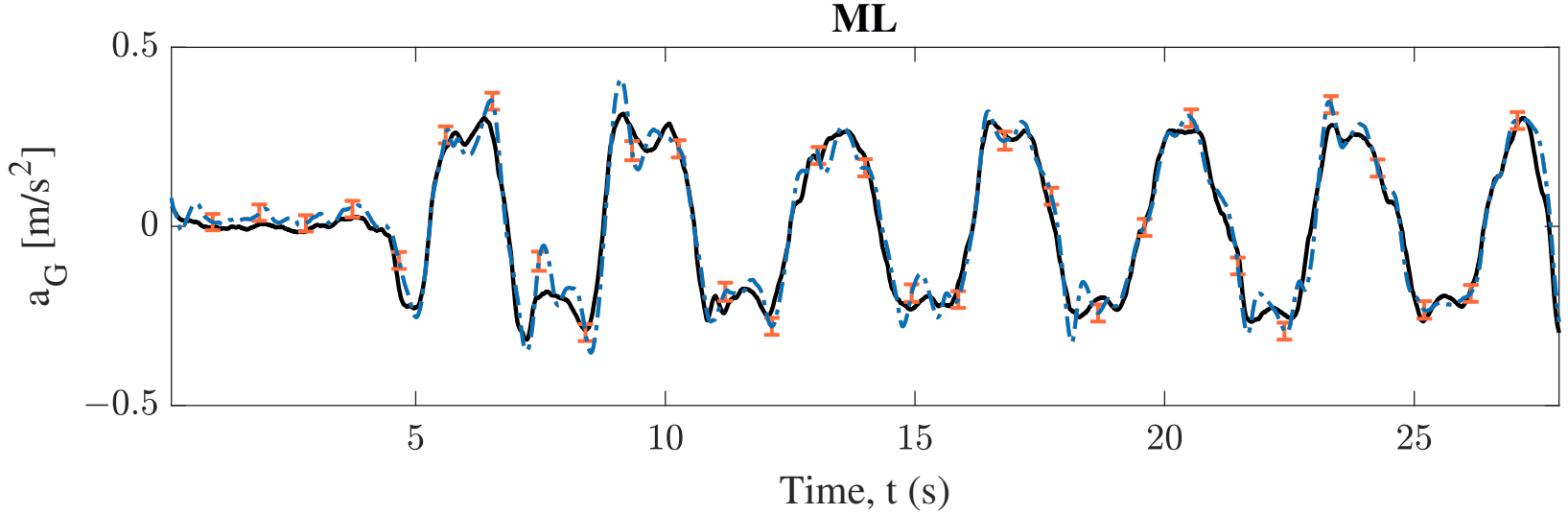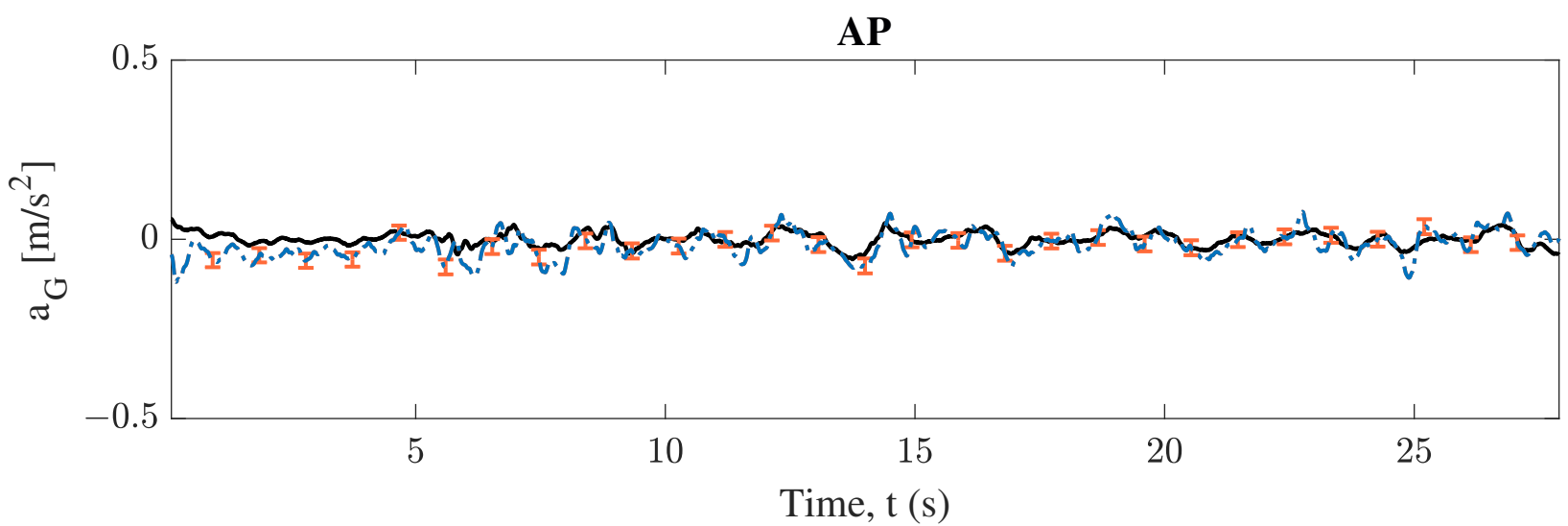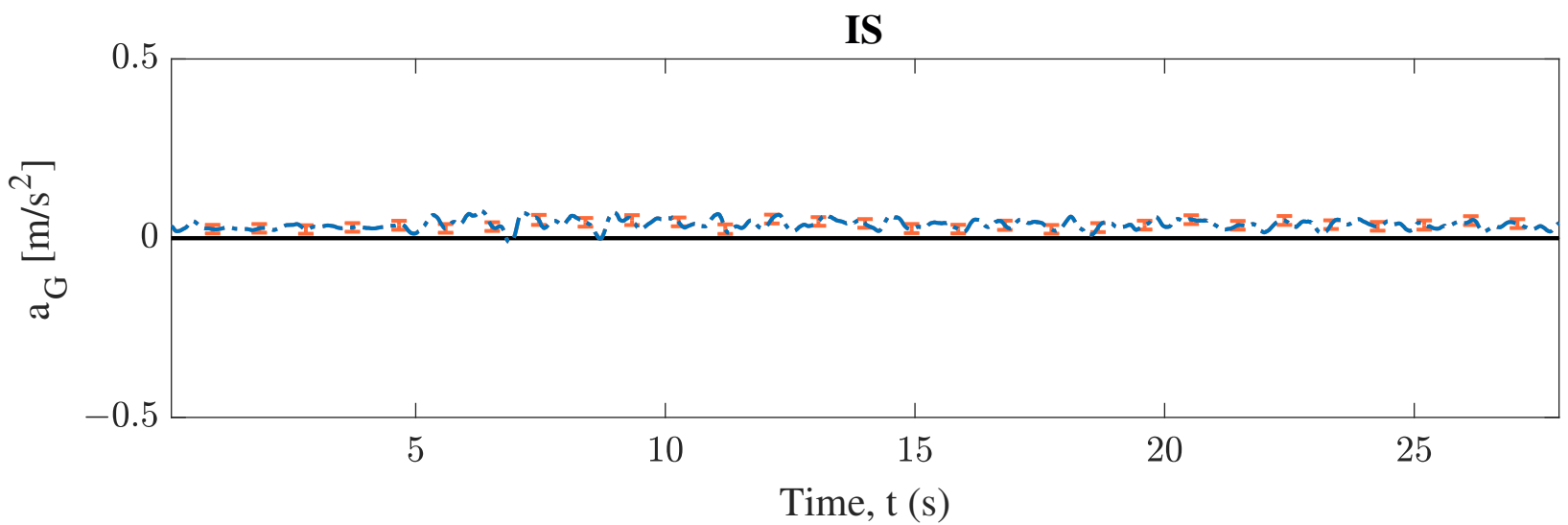

— FP    -.- BH

Supplement: Supplementary file 1 [file sensors-25-02639-s001.zip › Validation of Mobile Devices w Force Plates for Balance Assessment/figures/Results/Trial2_Fig2.pdf]

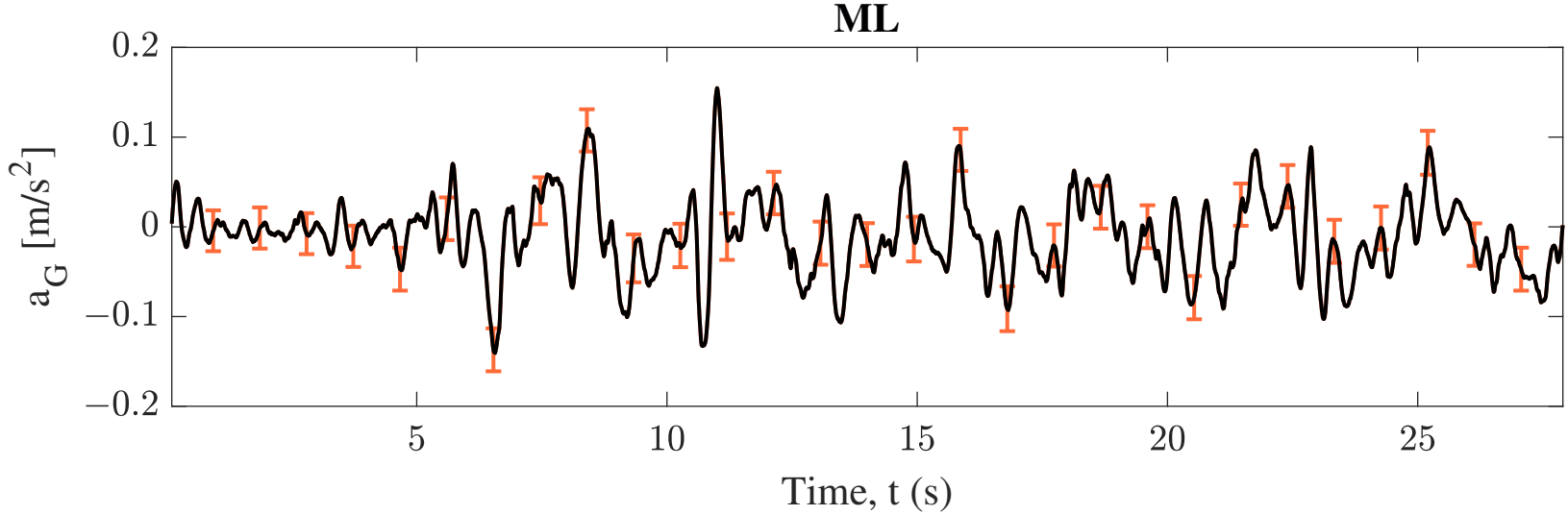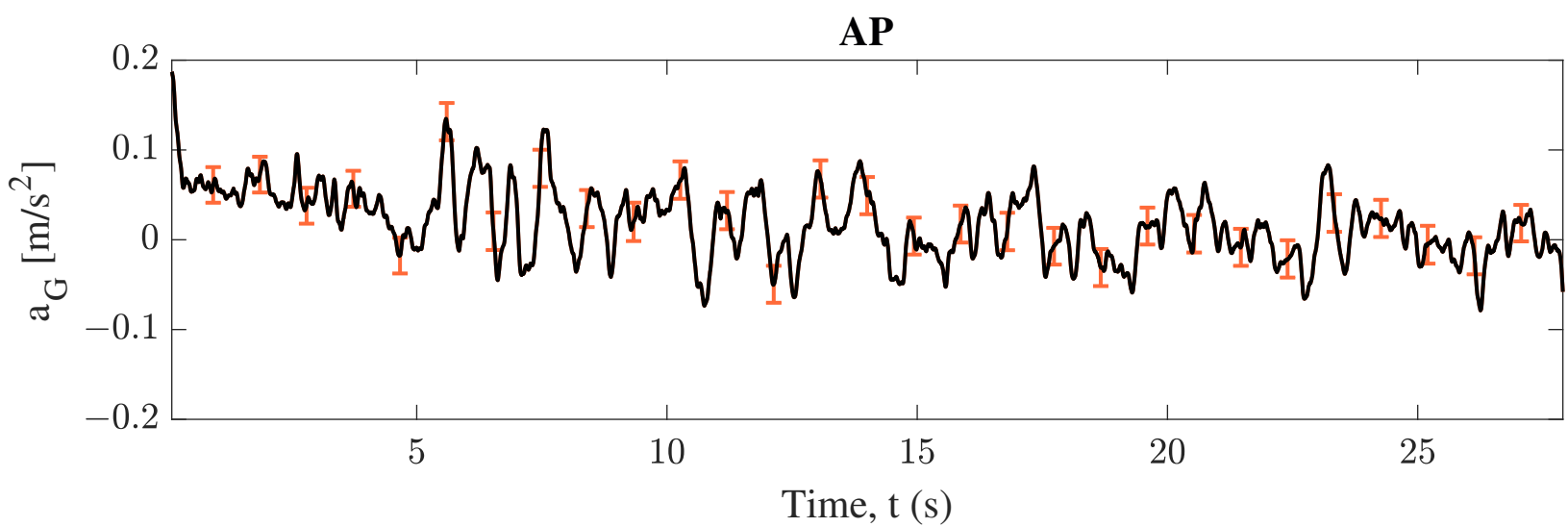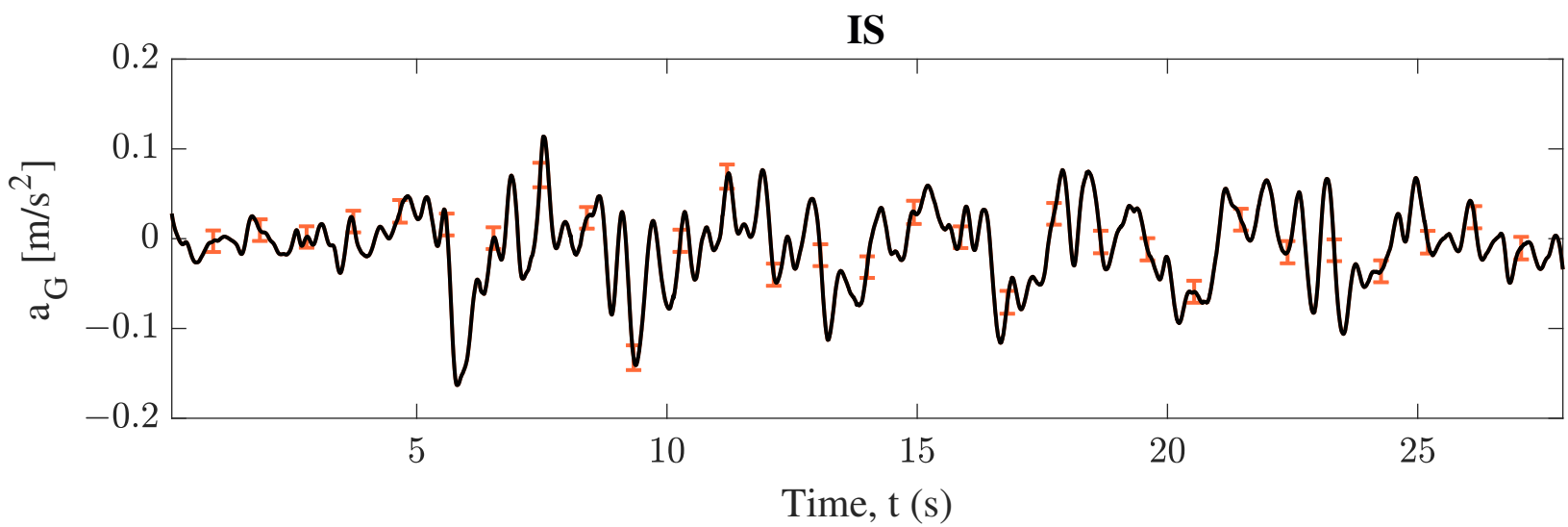

— FP — HH

Supplement: Supplementary file 1 [file sensors-25-02639-s001.zip › Validation of Mobile Devices w Force Plates for Balance Assessment/figures/Results/Trial2_Fig3.pdf]

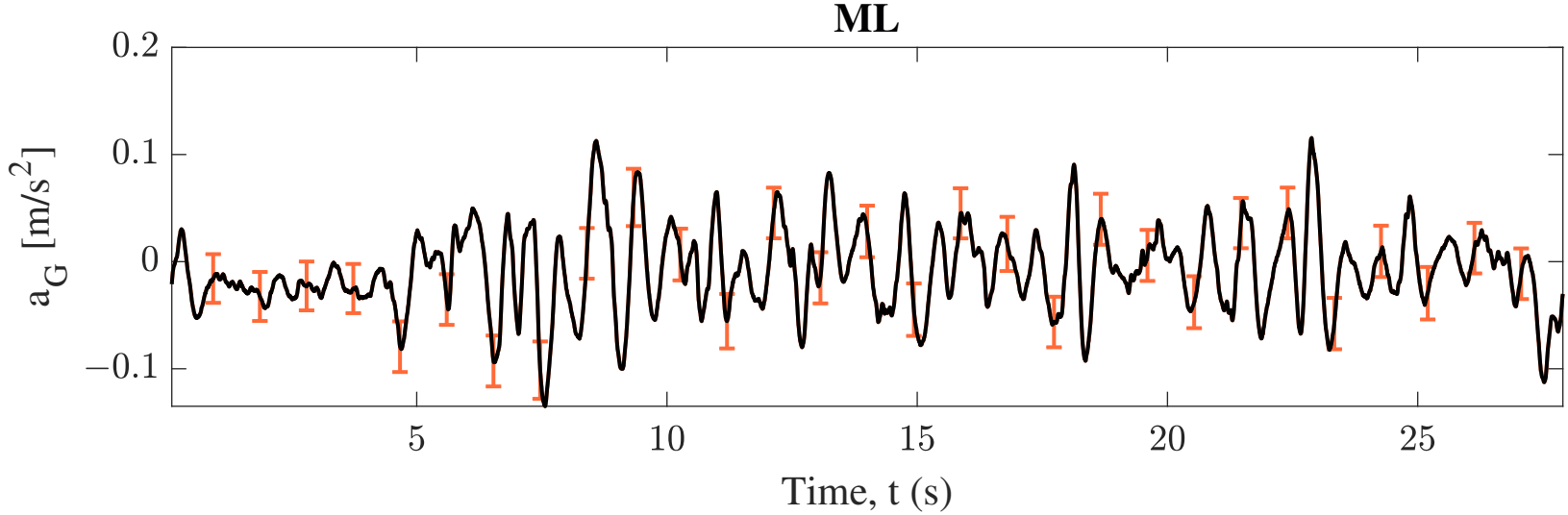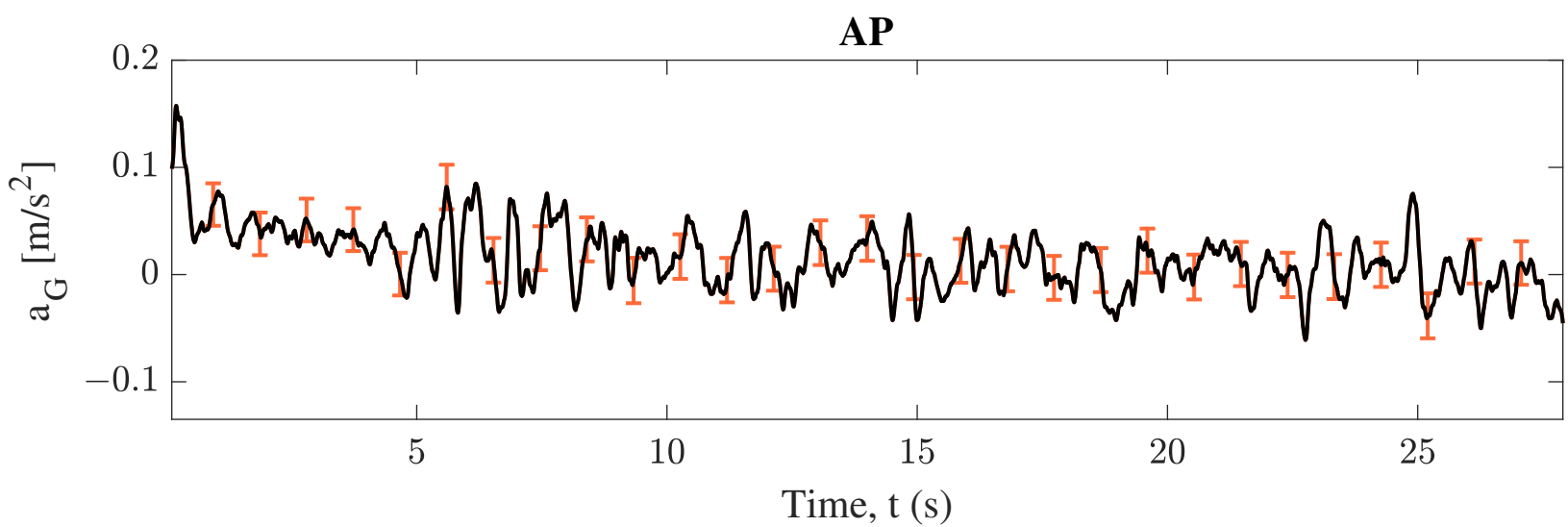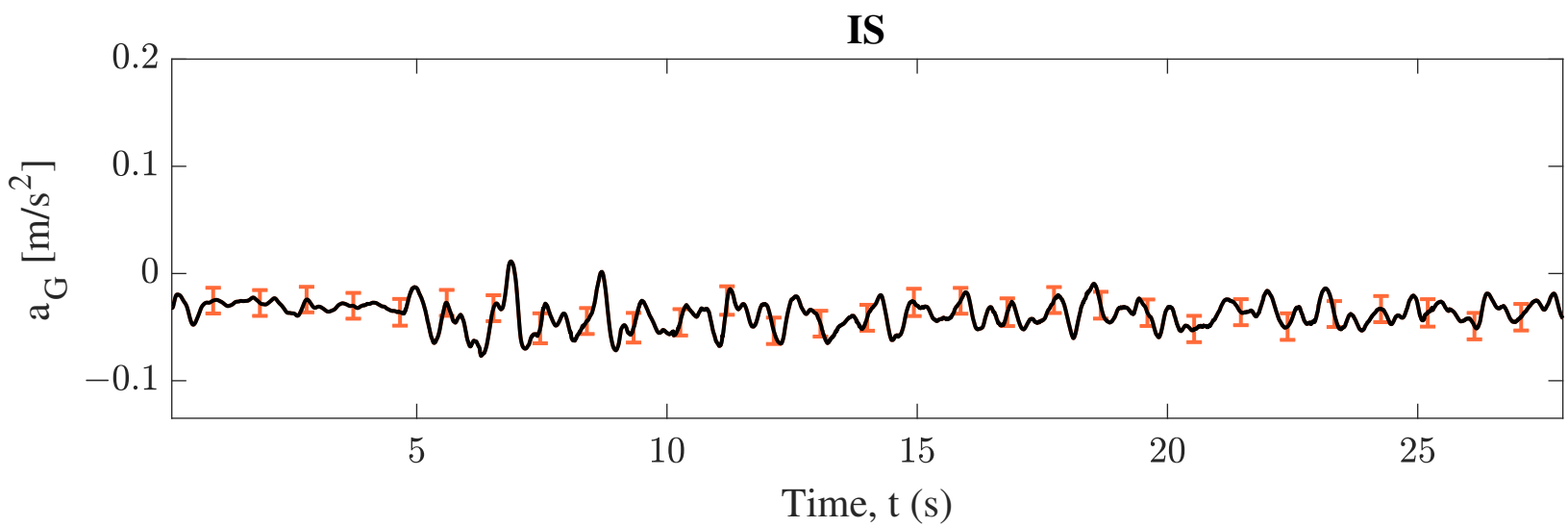

— FP — BH

Supplement: Supplementary file 1 [file sensors-25-02639-s001.zip › Validation of Mobile Devices w Force Plates for Balance Assessment/figures/Results/Trial2_Fig4.pdf]

**ML**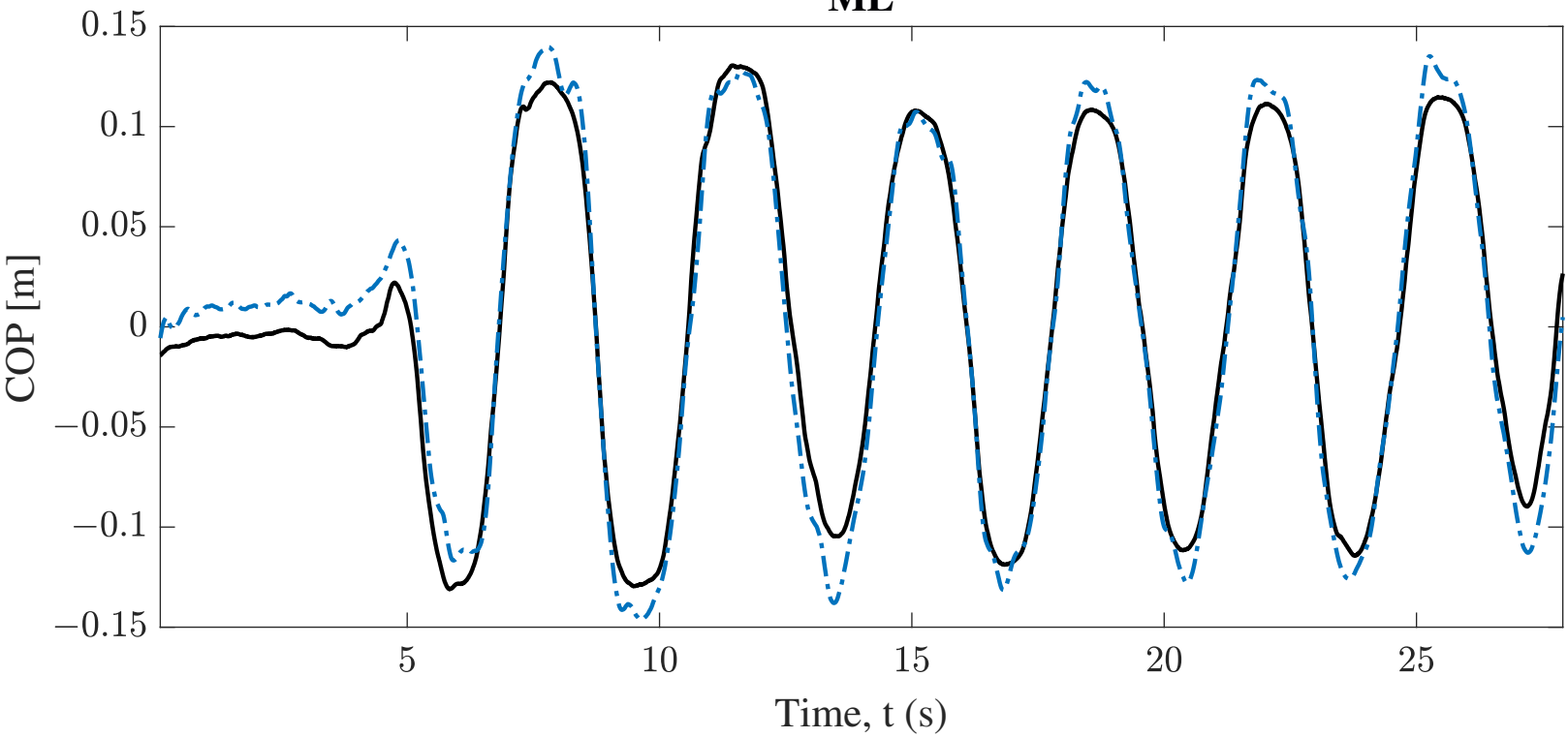**AP**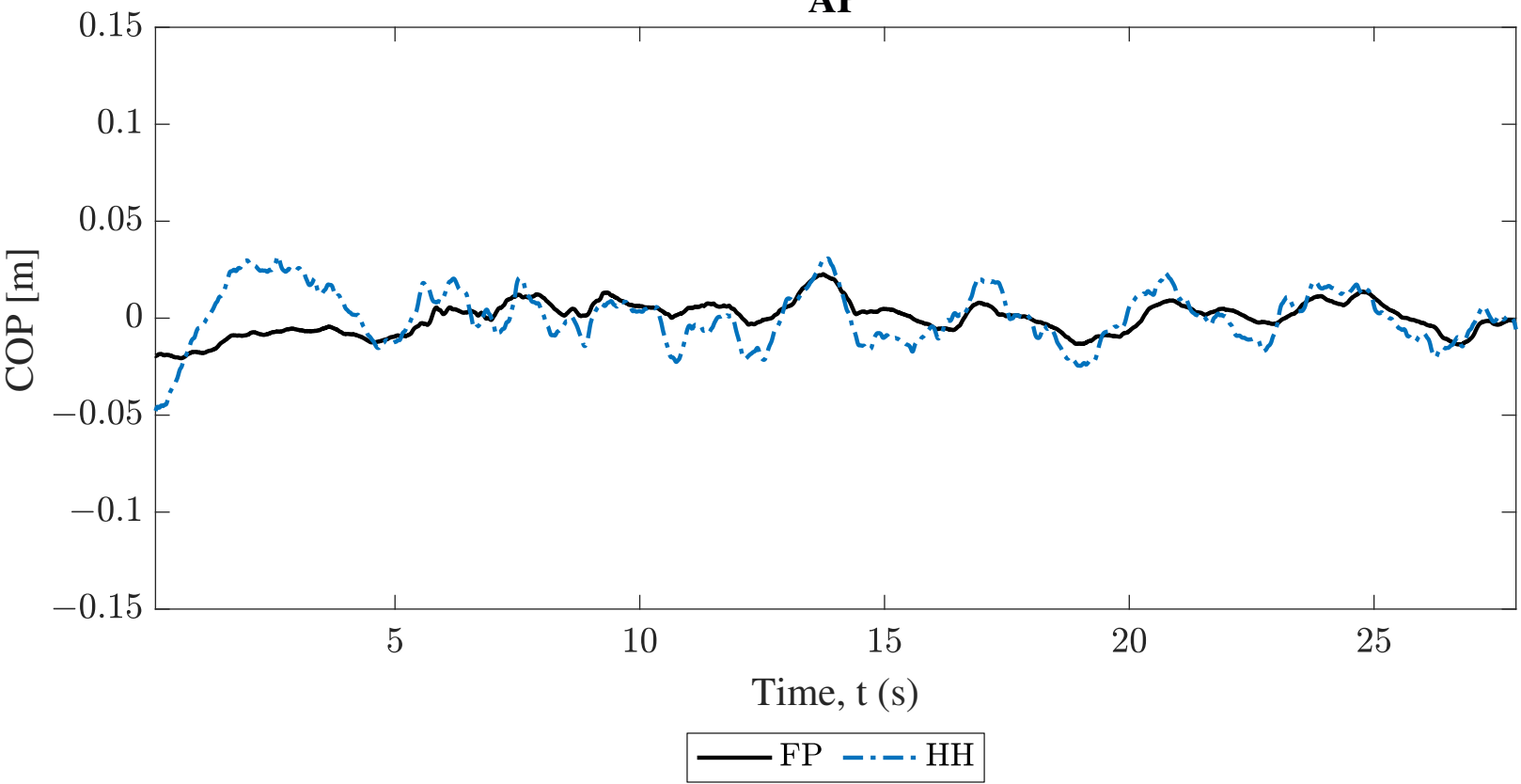

Supplement: Supplementary file 1 [file sensors-25-02639-s001.zip › Validation of Mobile Devices w Force Plates for Balance Assessment/figures/Results/Trial2_Fig5.pdf]

**ML**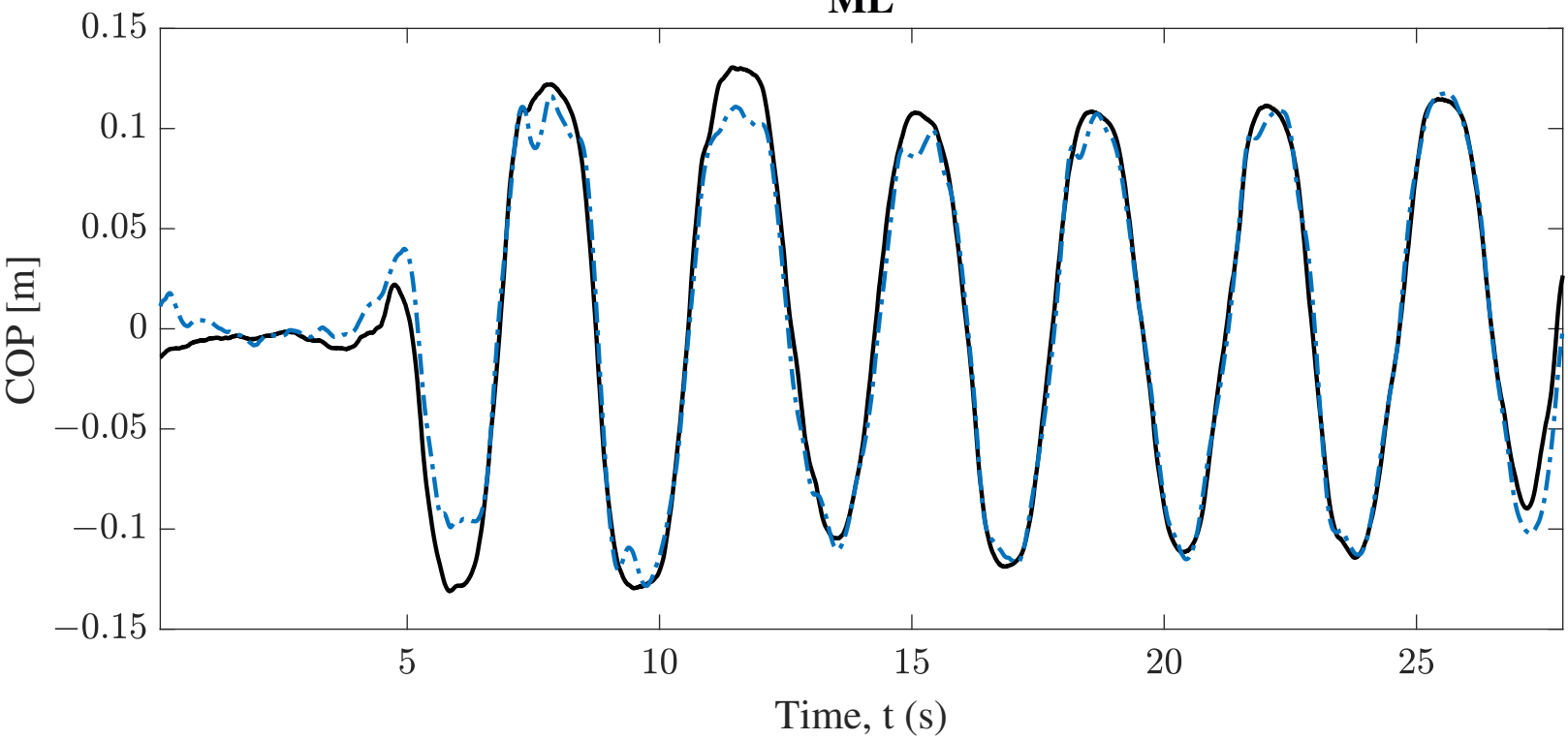**AP**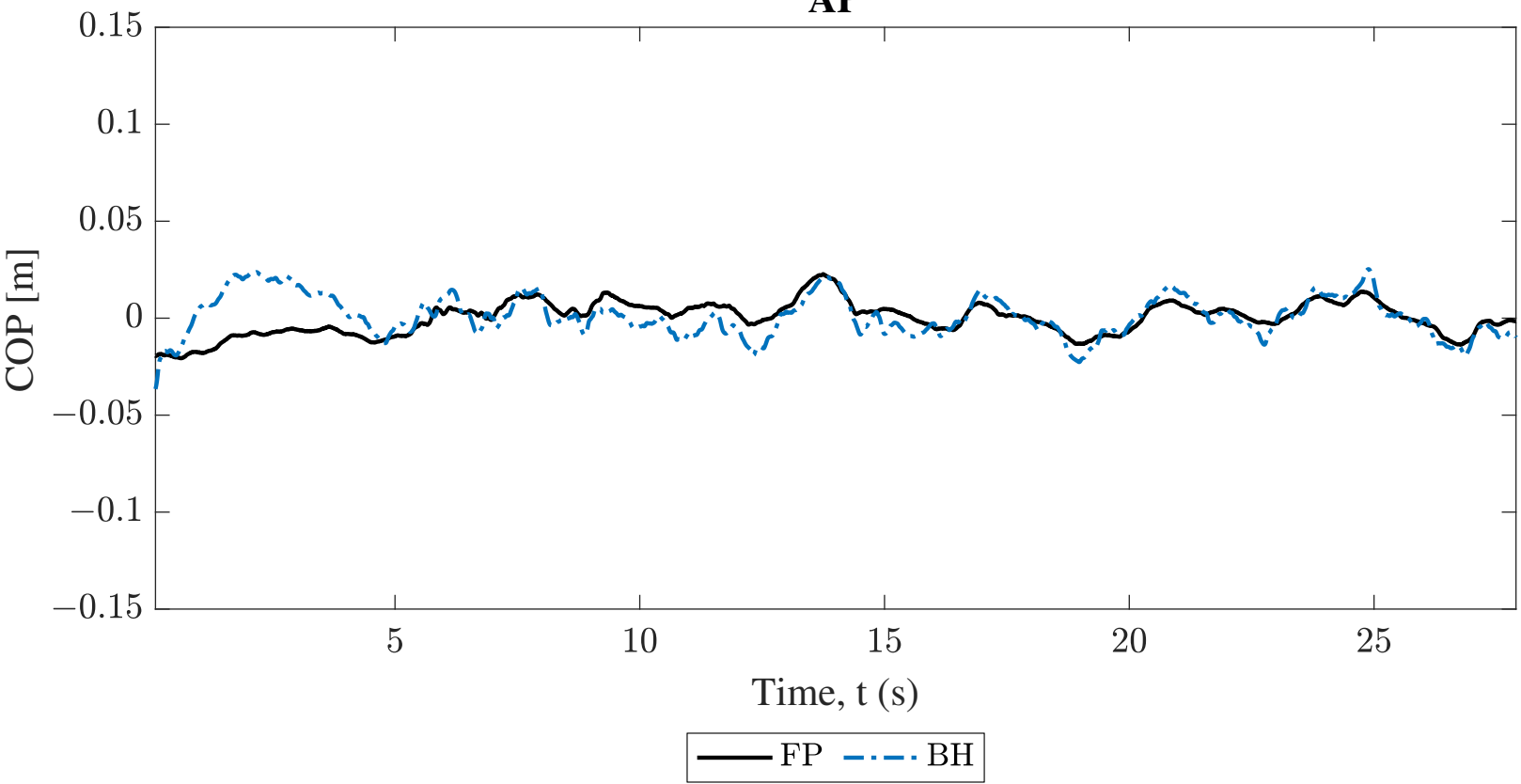

Supplement: Supplementary file 1 [file sensors-25-02639-s001.zip › Validation of Mobile Devices w Force Plates for Balance Assessment/figures/Results/Trial2_Fig6.pdf]

**ML**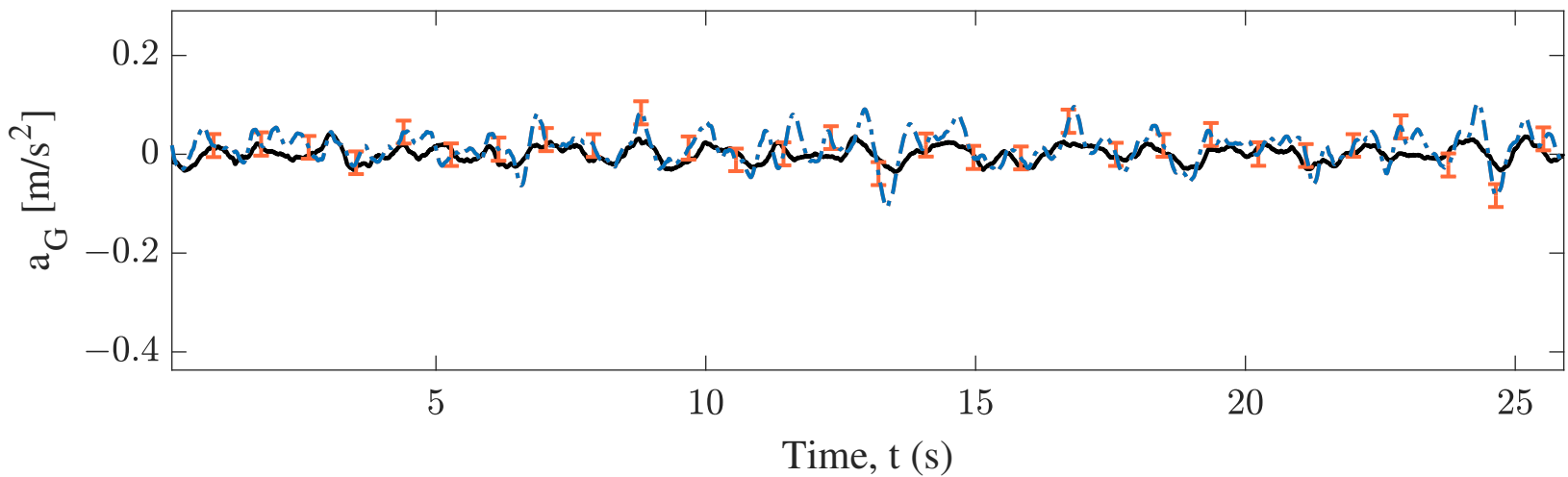**AP**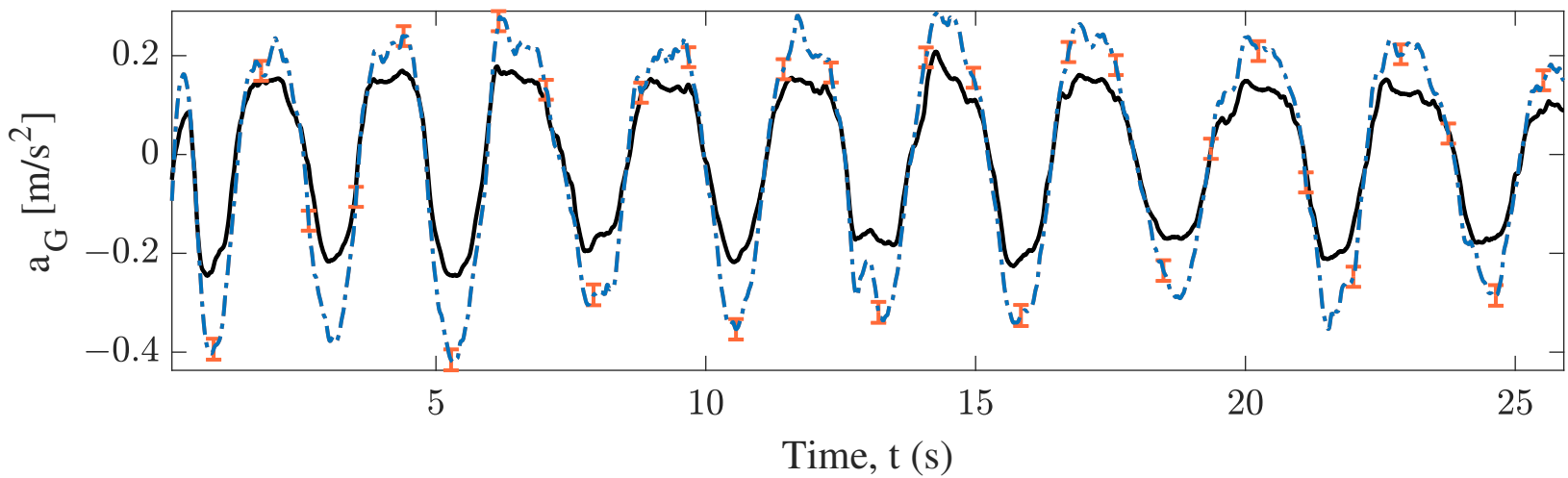**IS**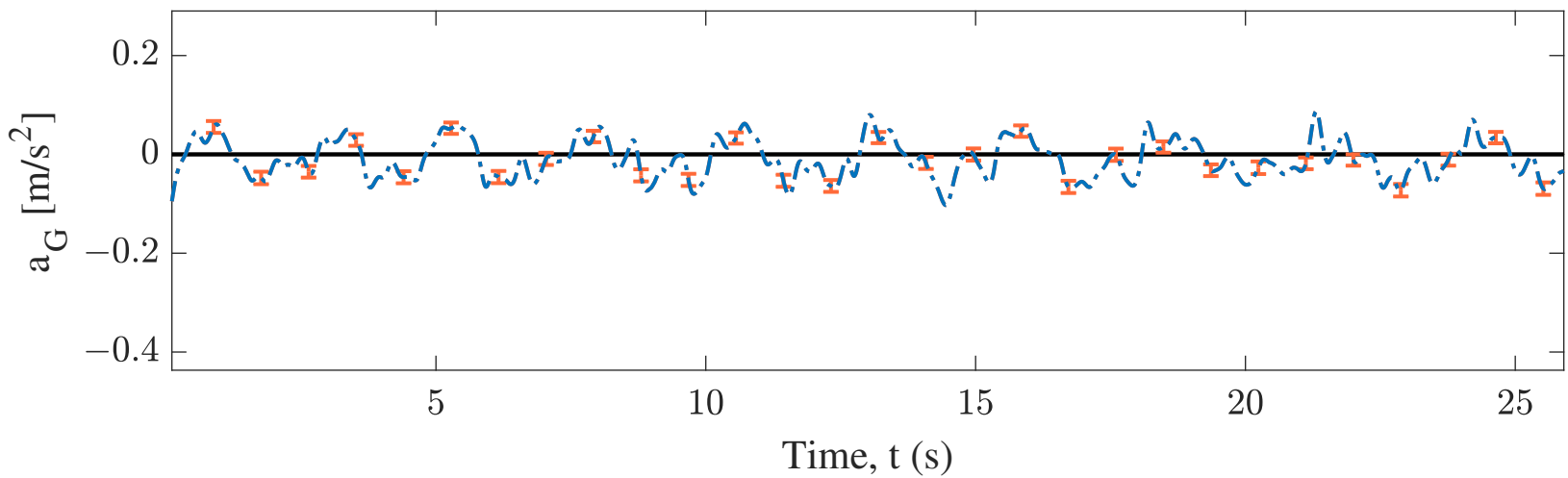

— FP    - - - HH

Supplement: Supplementary file 1 [file sensors-25-02639-s001.zip › Validation of Mobile Devices w Force Plates for Balance Assessment/figures/Results/Trial3_Fig1.pdf]

**ML**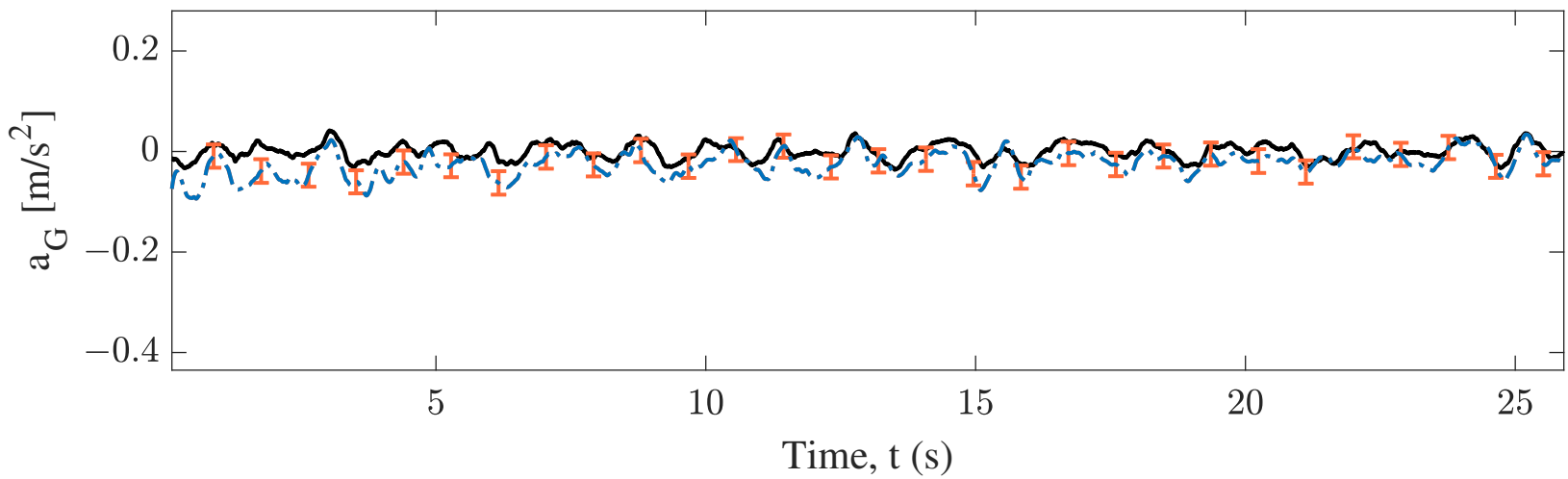**AP**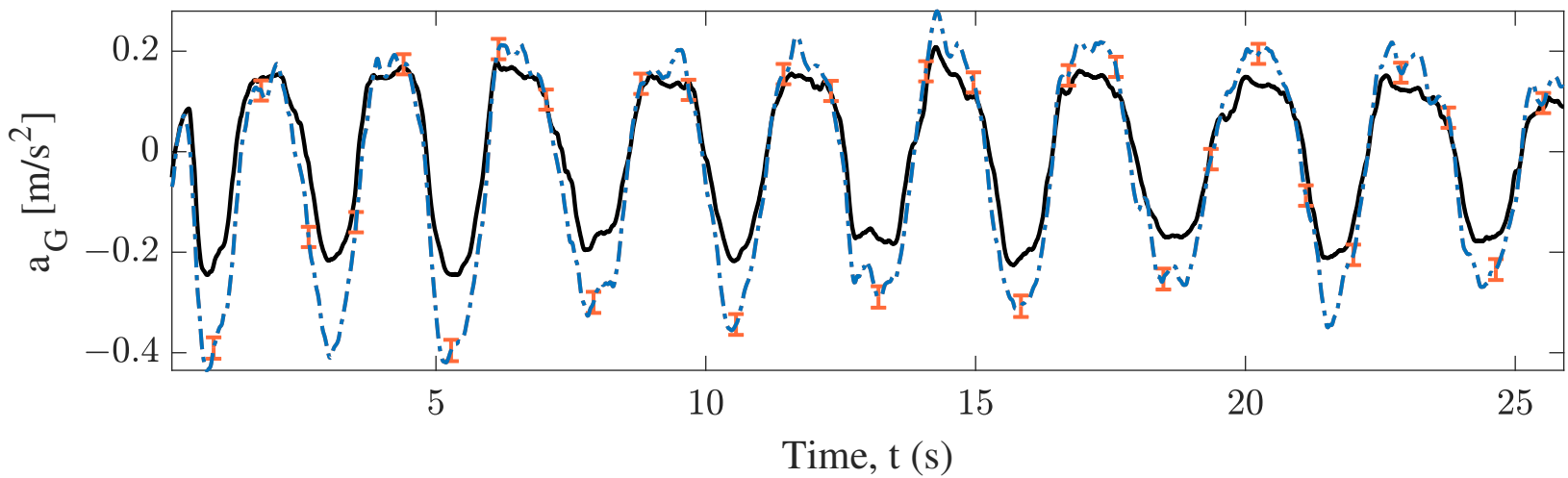**IS**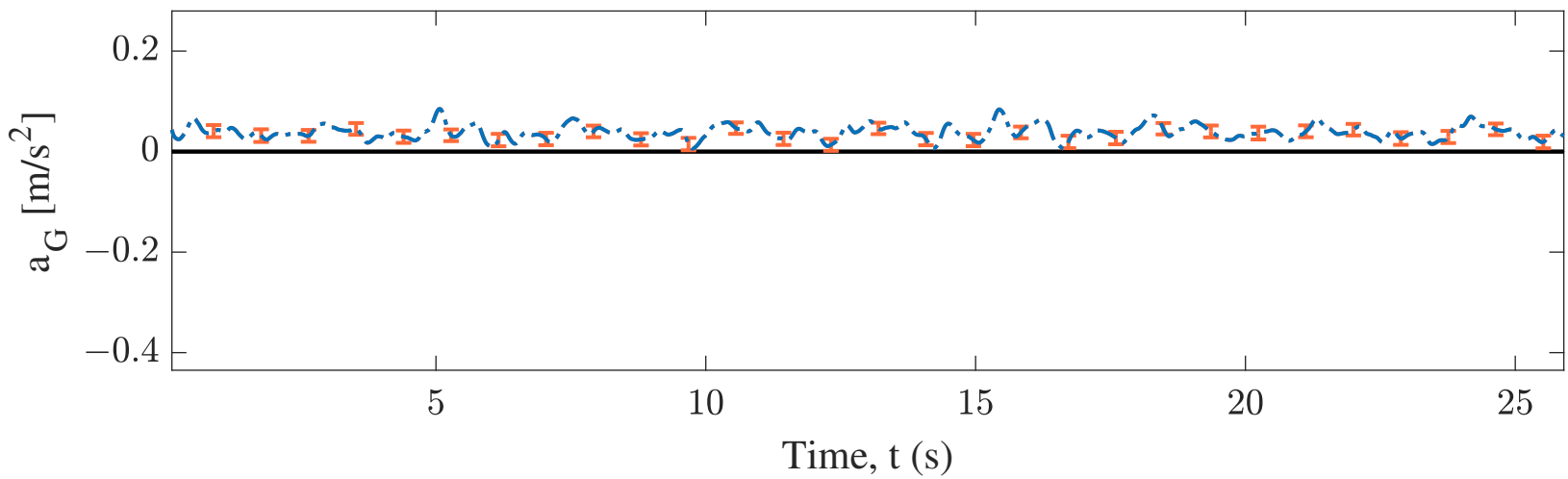

— FP    -.- BH

Supplement: Supplementary file 1 [file sensors-25-02639-s001.zip › Validation of Mobile Devices w Force Plates for Balance Assessment/figures/Results/Trial3_Fig2.pdf]

**ML**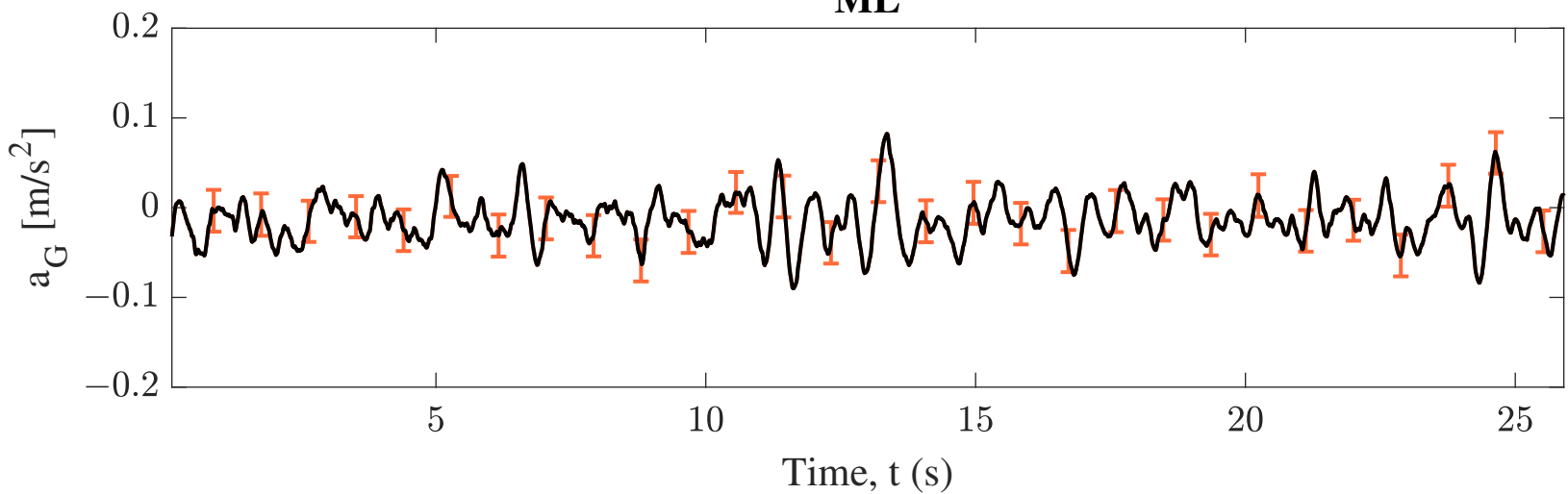**AP**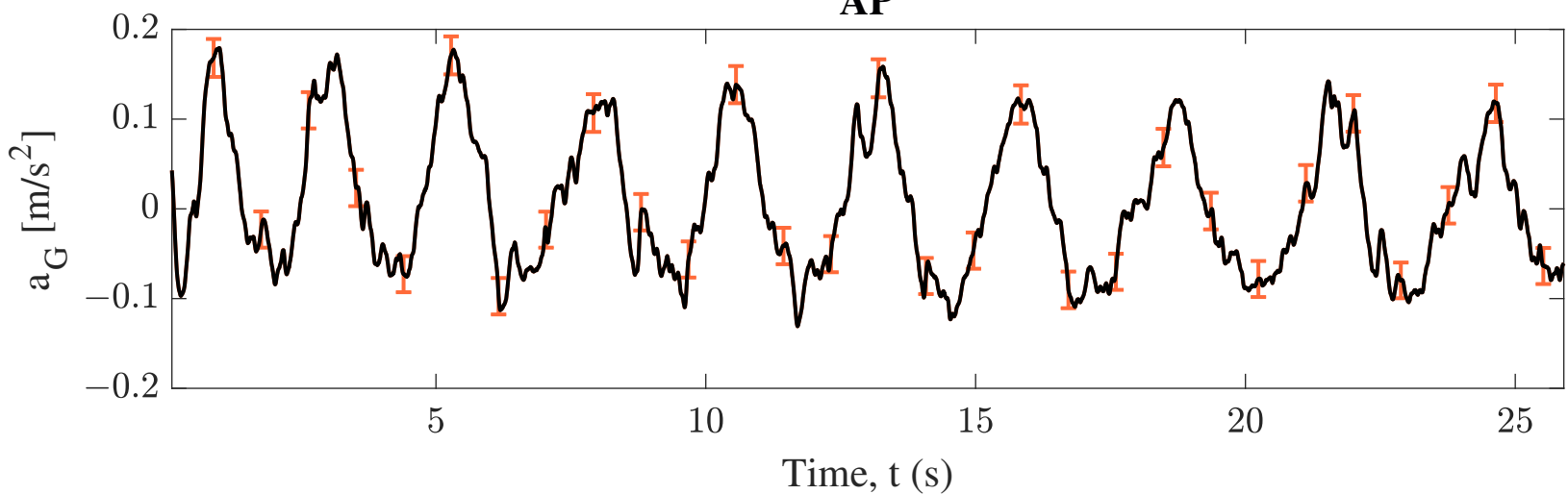**IS**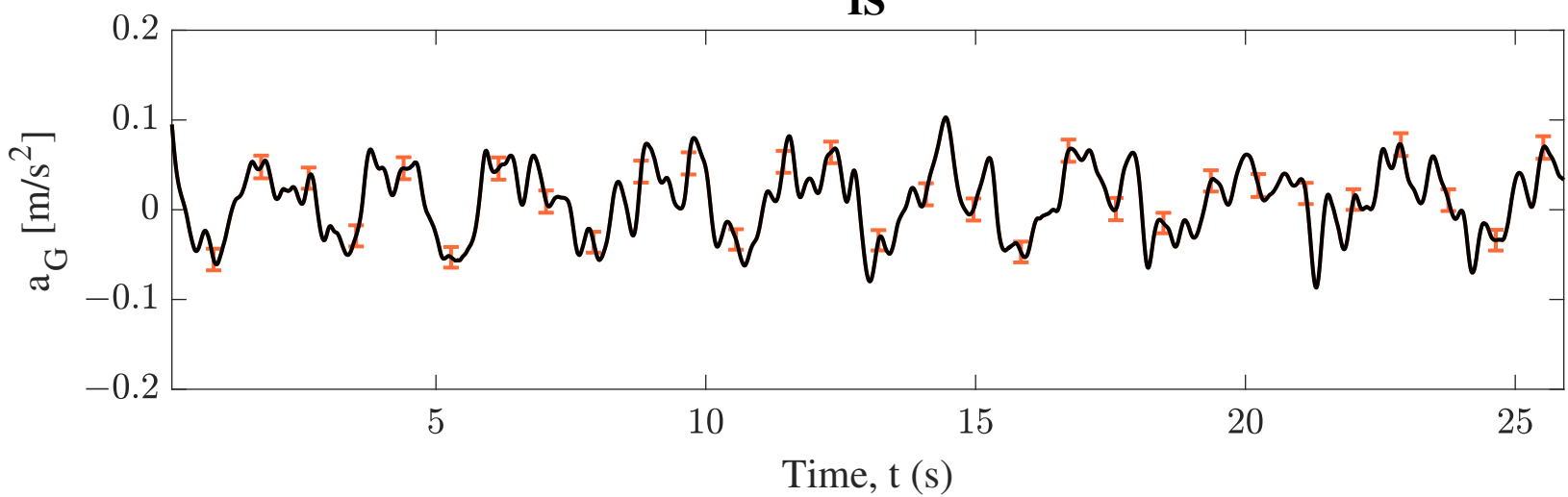

— FP – HH

Supplement: Supplementary file 1 [file sensors-25-02639-s001.zip › Validation of Mobile Devices w Force Plates for Balance Assessment/figures/Results/Trial3_Fig3.pdf]

**ML**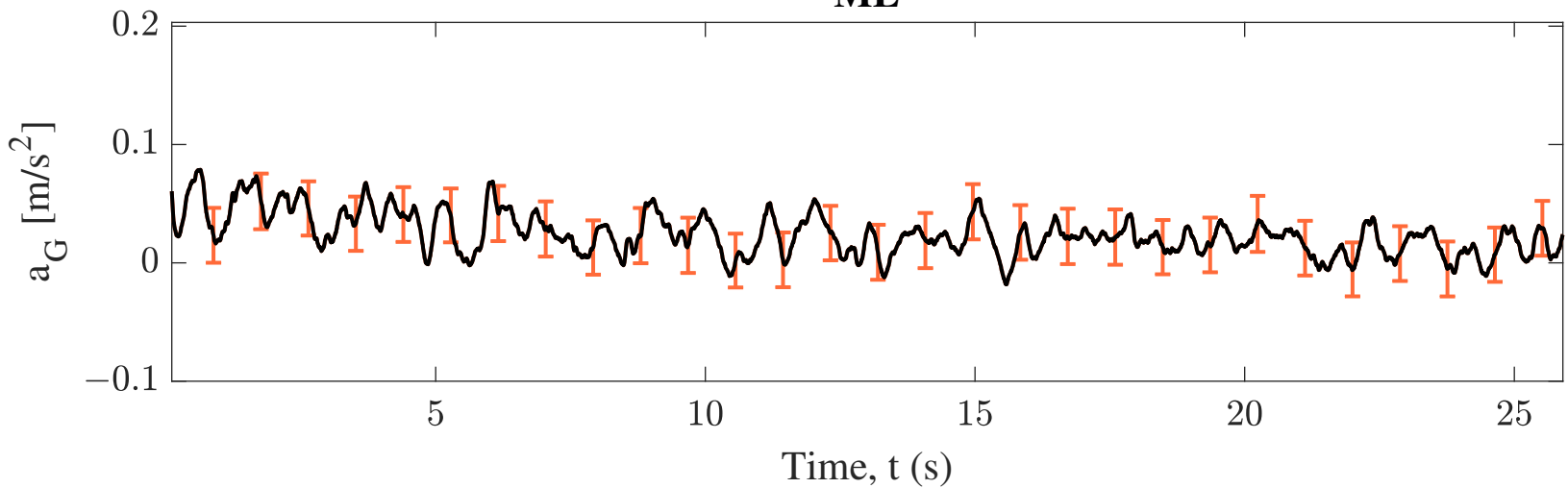**AP**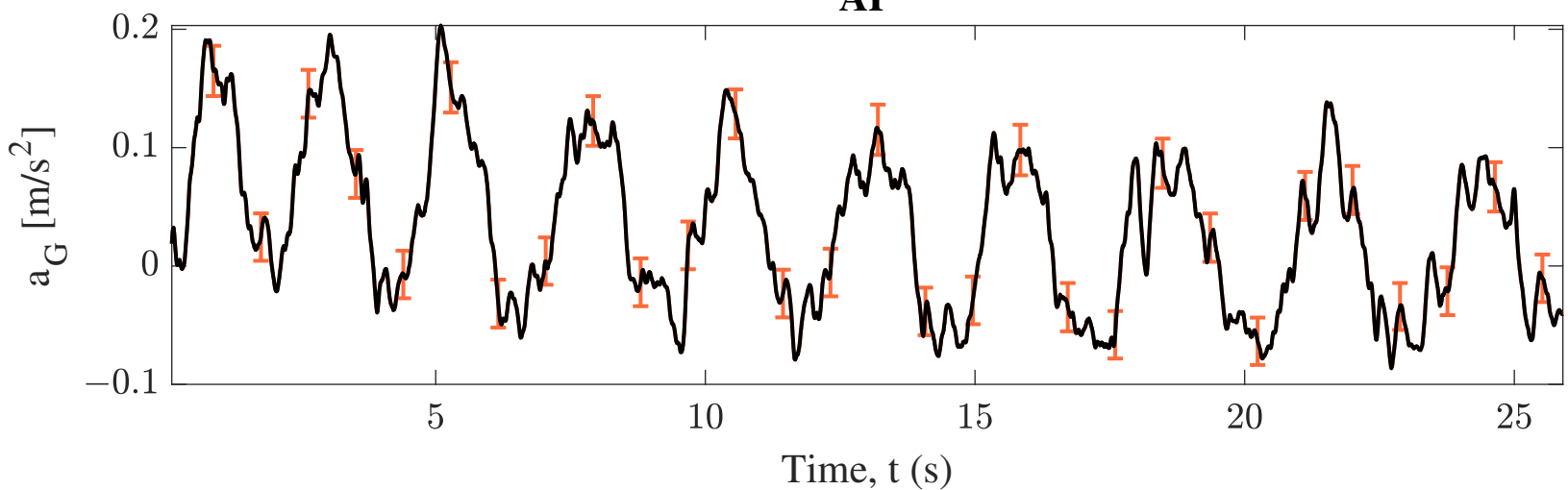**IS**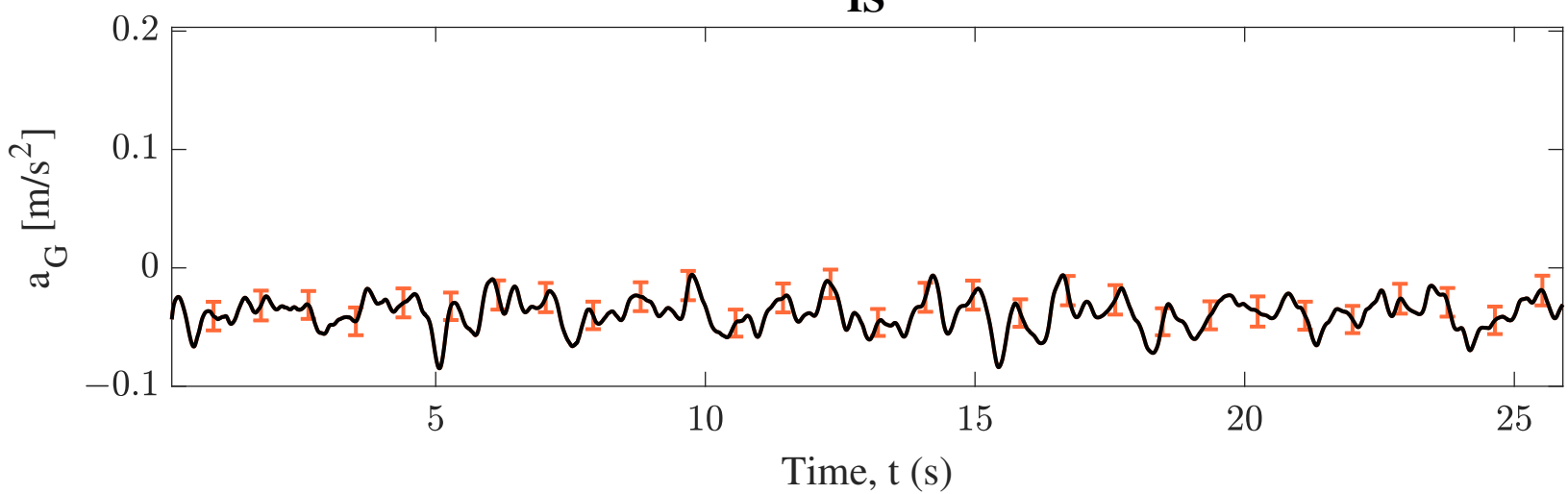

— FP - BH

Supplement: Supplementary file 1 [file sensors-25-02639-s001.zip › Validation of Mobile Devices w Force Plates for Balance Assessment/figures/Results/Trial3_Fig4.pdf]

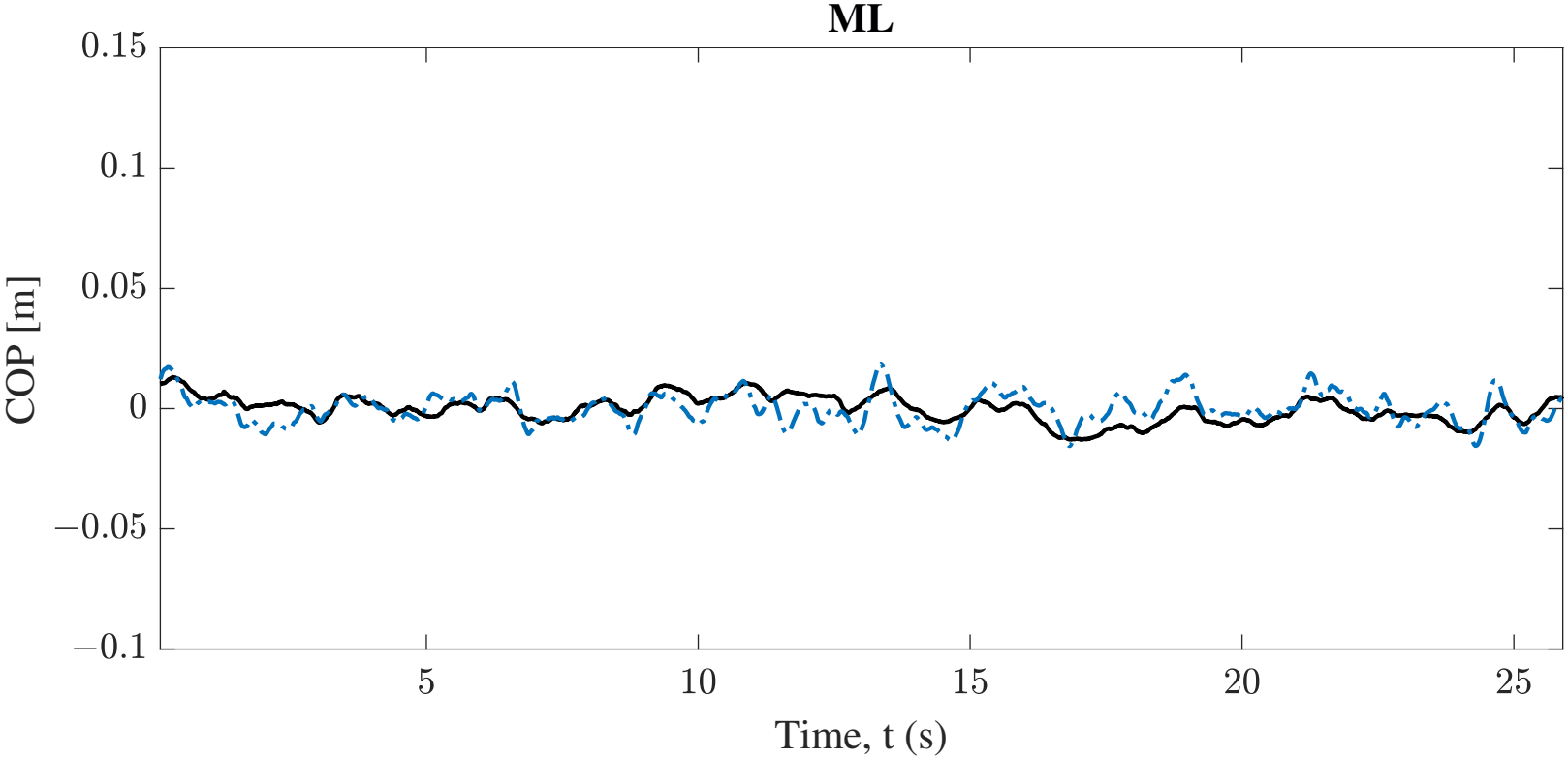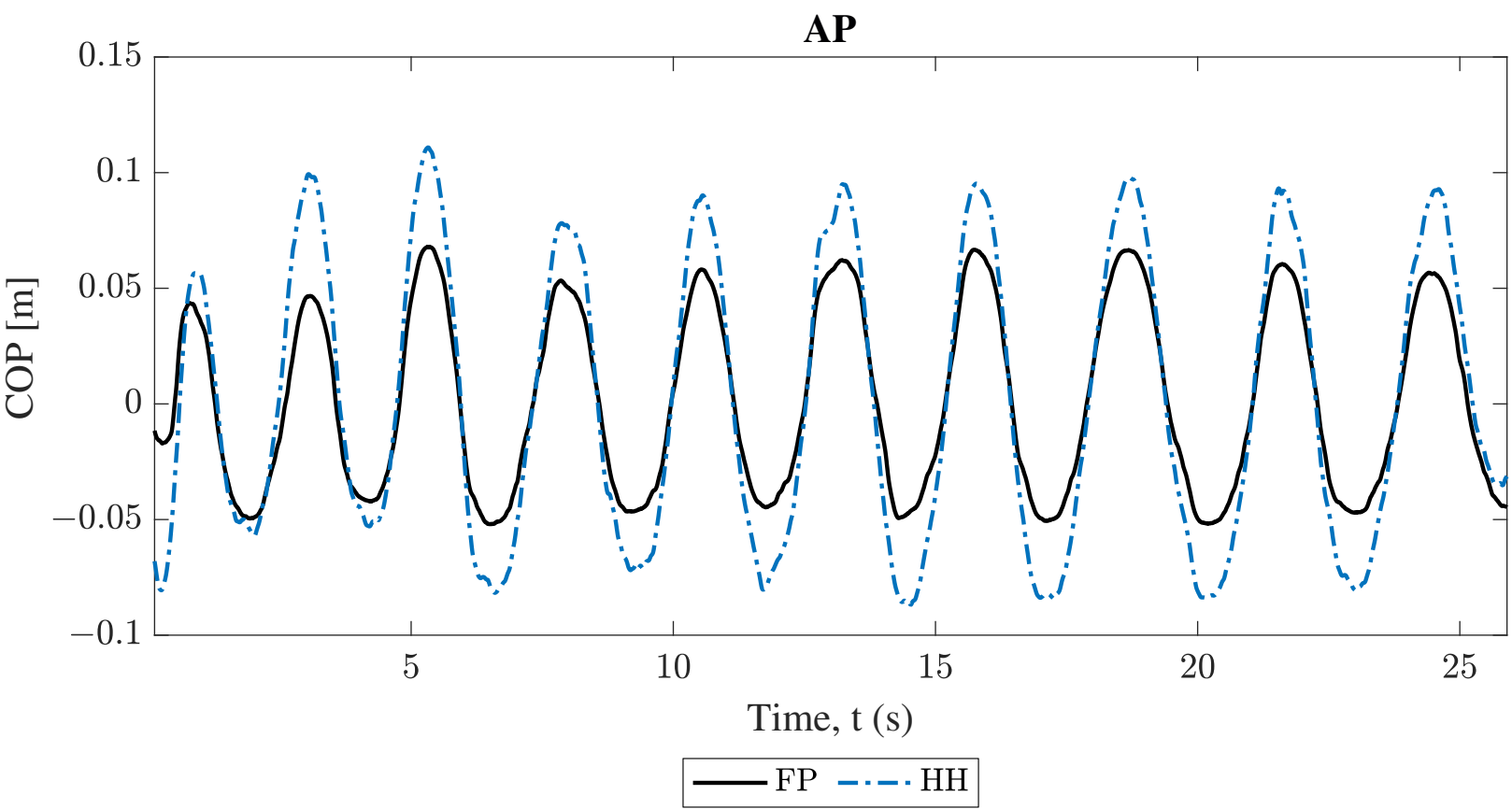

Supplement: Supplementary file 1 [file sensors-25-02639-s001.zip › Validation of Mobile Devices w Force Plates for Balance Assessment/figures/Results/Trial3_Fig5.pdf]

**ML**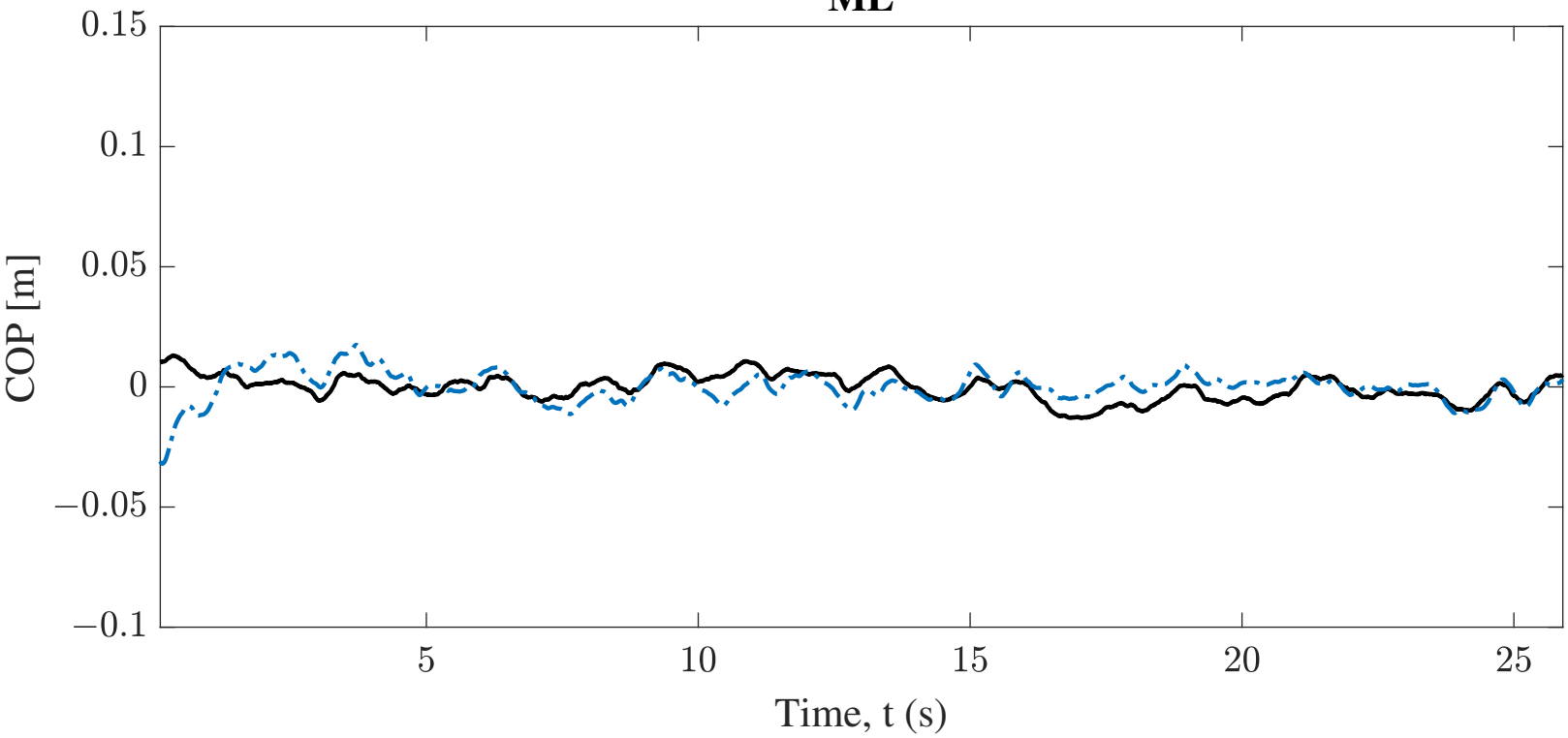**AP**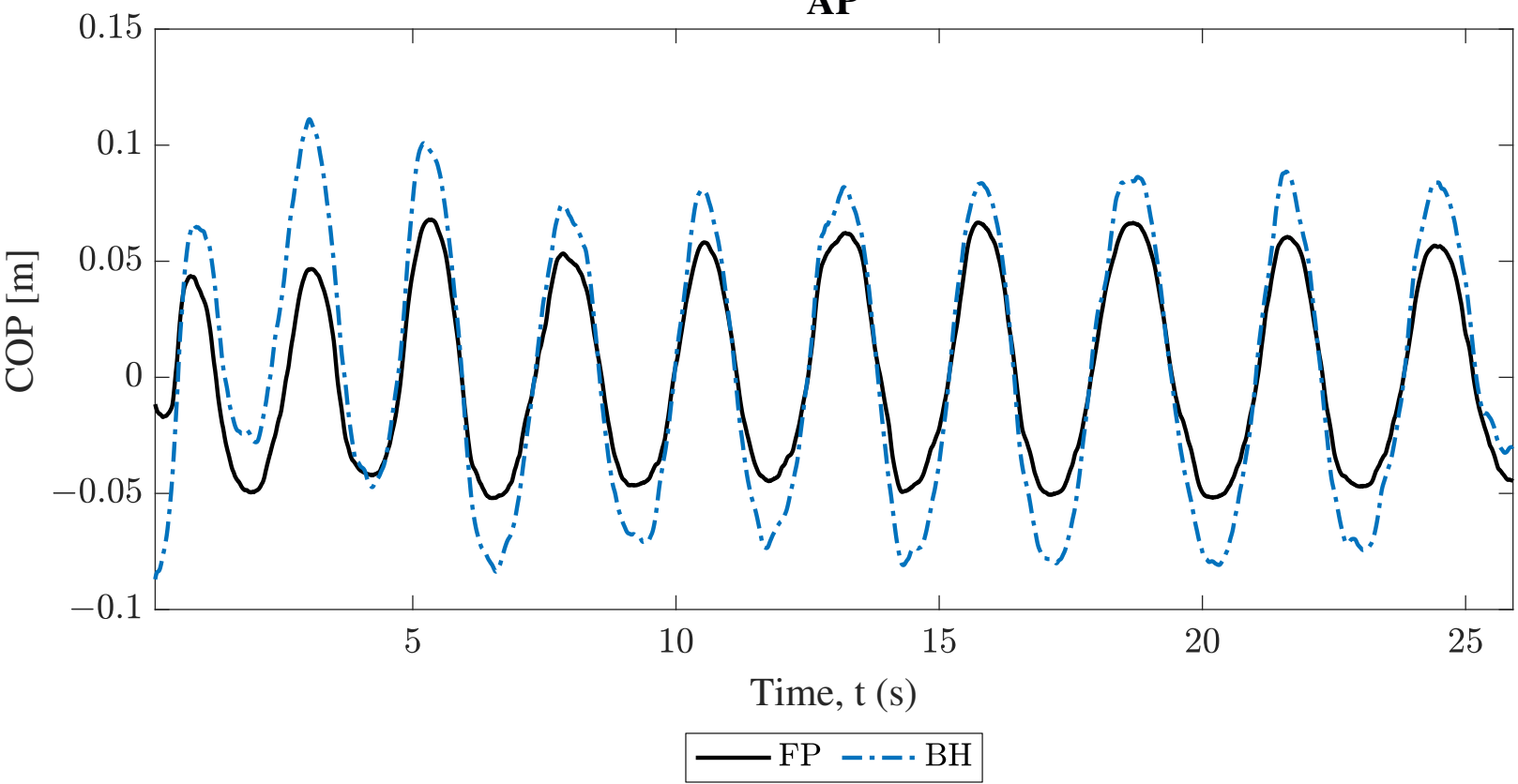

Supplement: Supplementary file 1 [file sensors-25-02639-s001.zip › Validation of Mobile Devices w Force Plates for Balance Assessment/figures/Results/Trial3_Fig6.pdf]

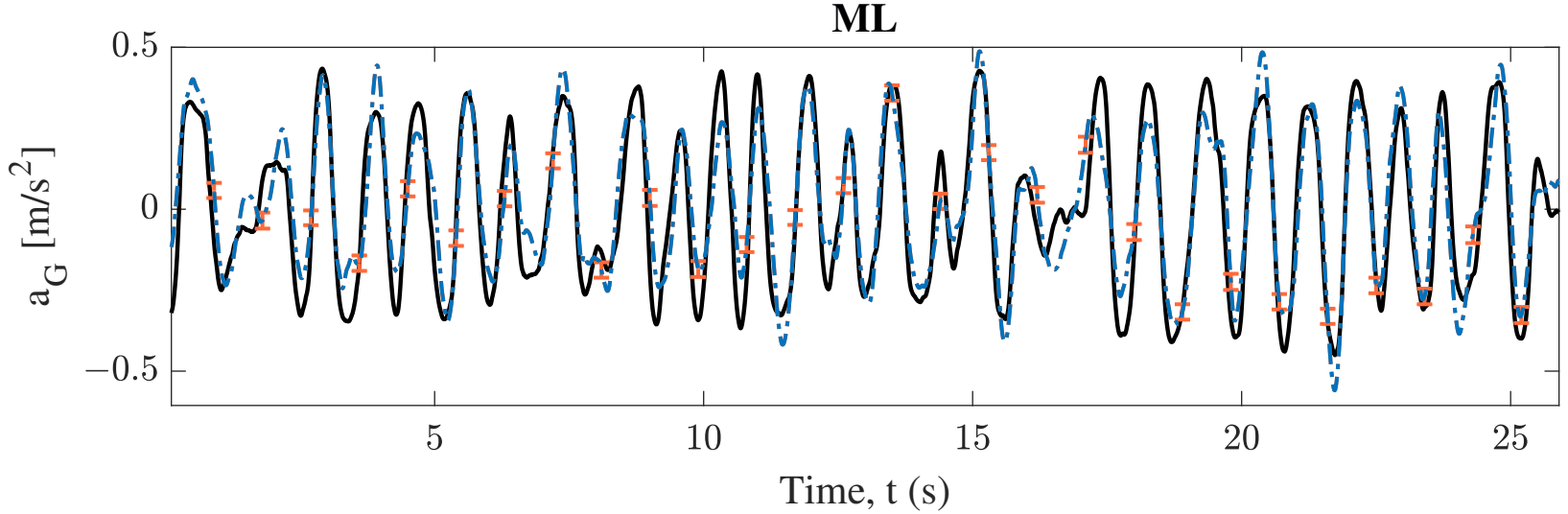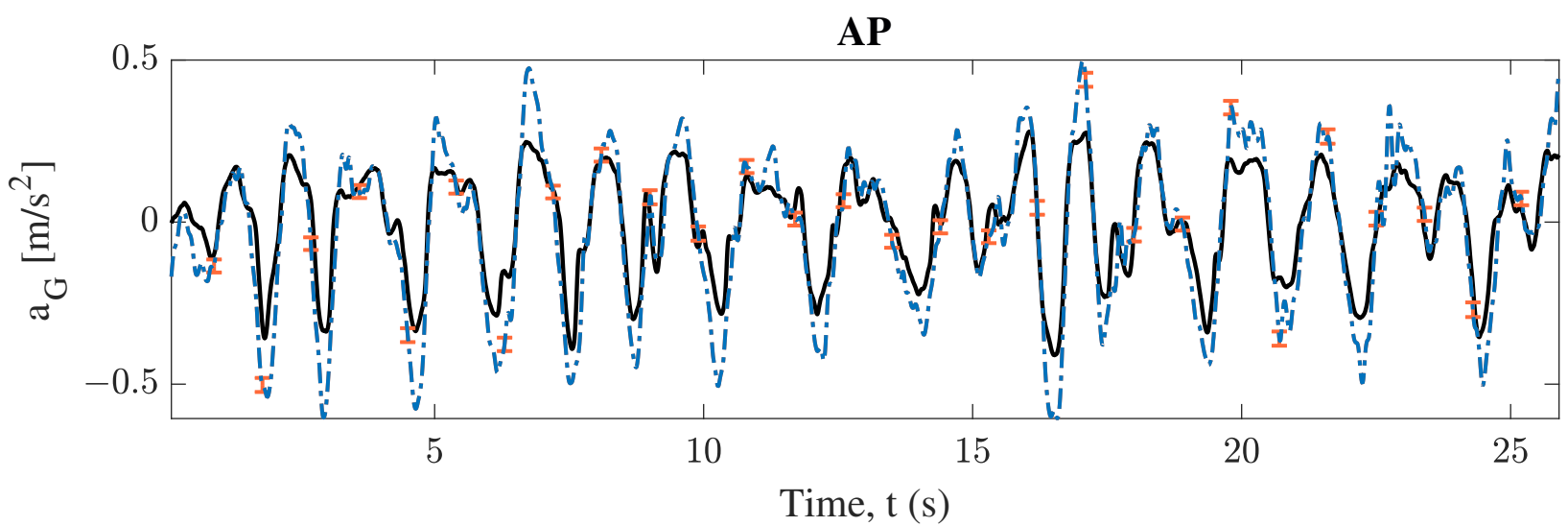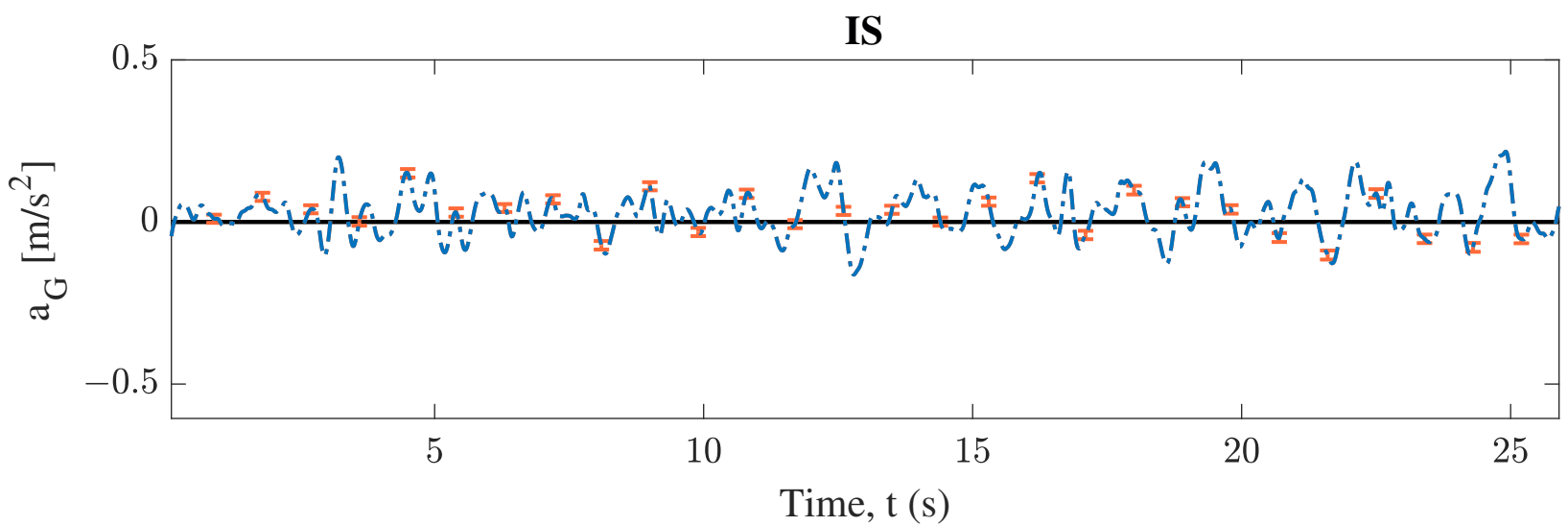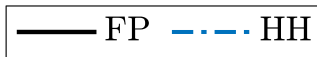

Supplement: Supplementary file 1 [file sensors-25-02639-s001.zip › Validation of Mobile Devices w Force Plates for Balance Assessment/figures/Results/Trial4_Fig1.pdf]

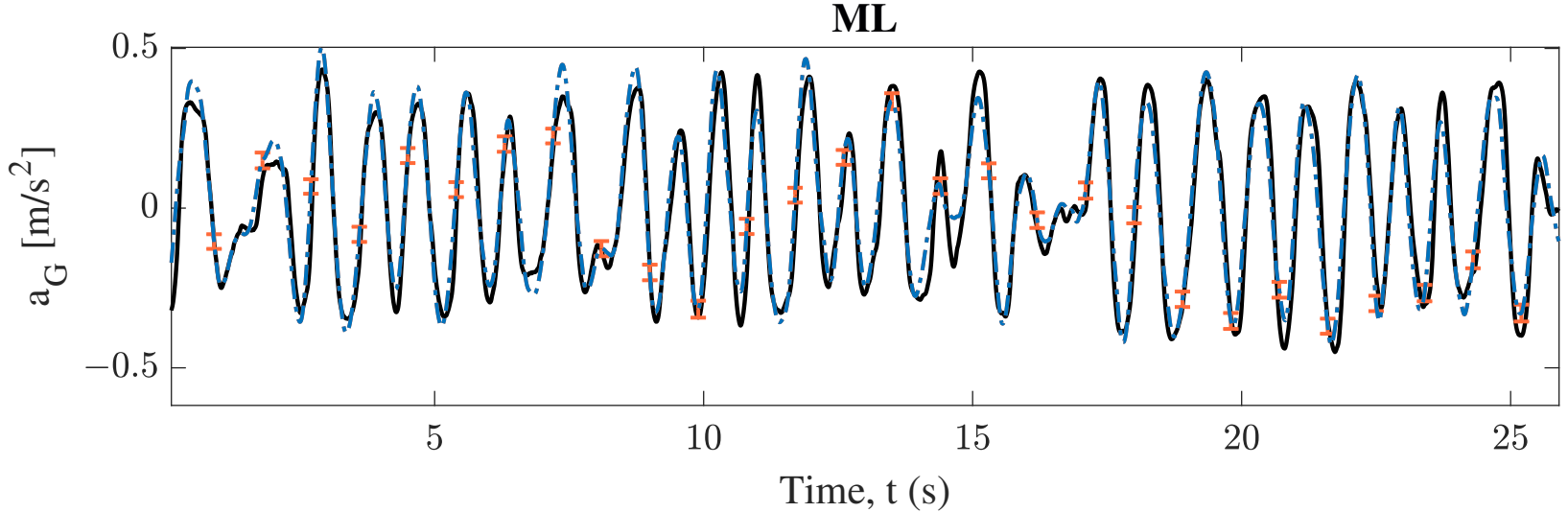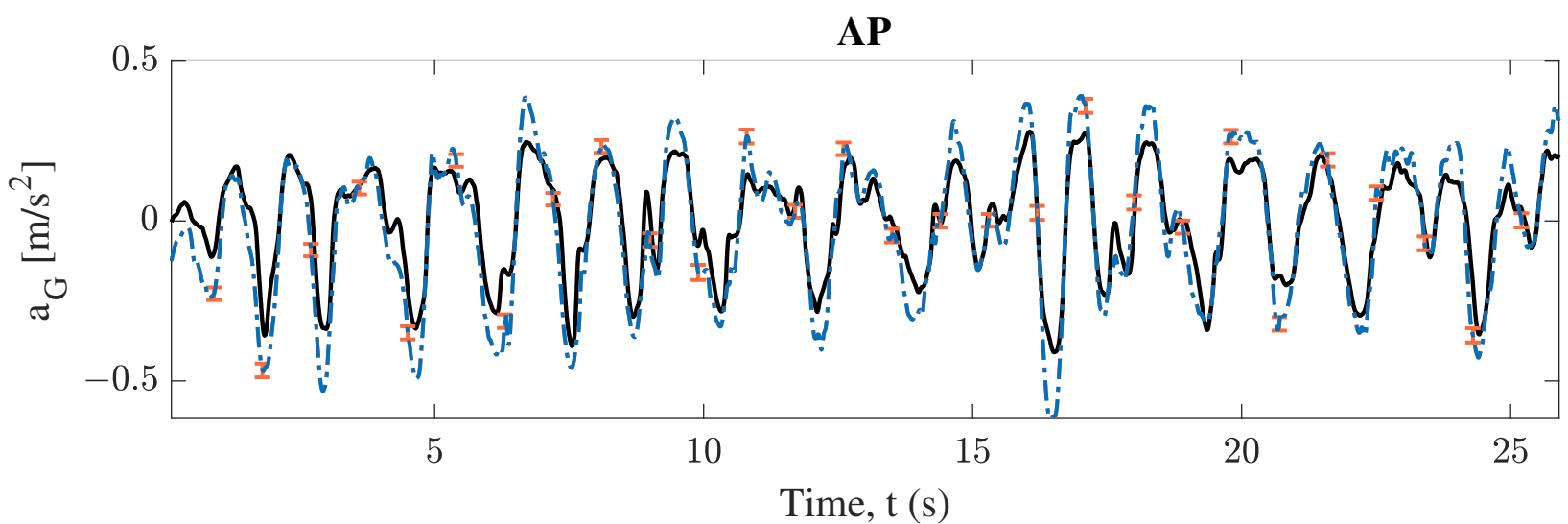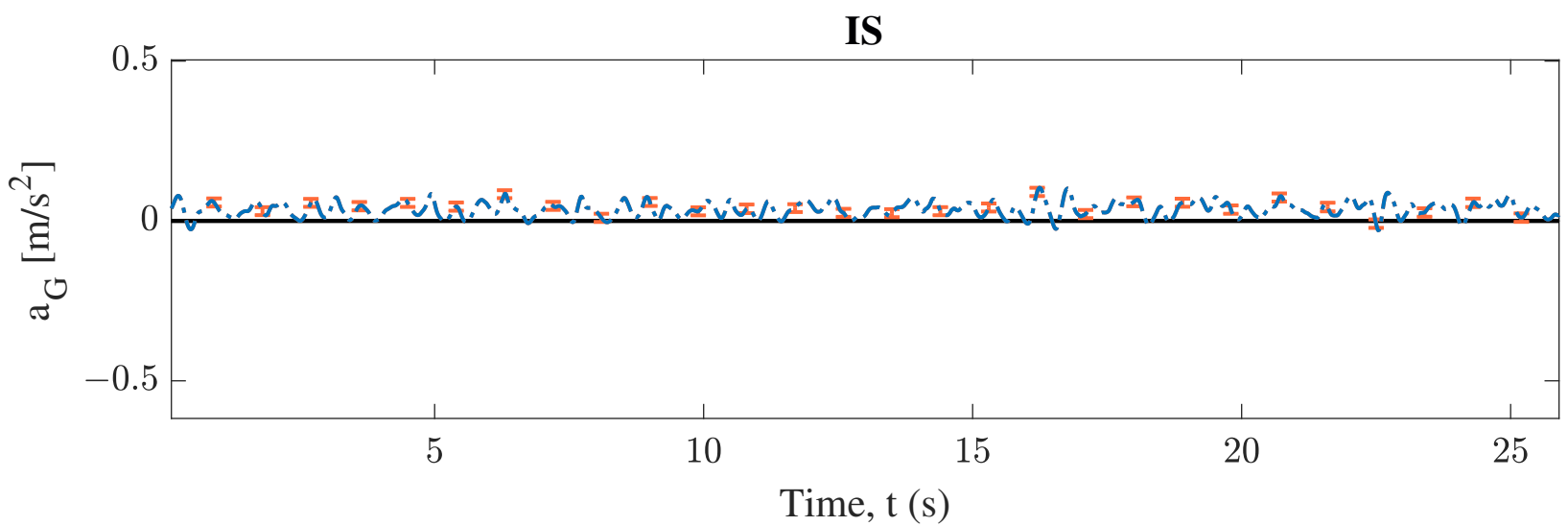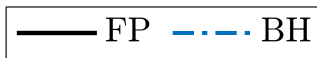

Supplement: Supplementary file 1 [file sensors-25-02639-s001.zip › Validation of Mobile Devices w Force Plates for Balance Assessment/figures/Results/Trial4_Fig2.pdf]

**ML**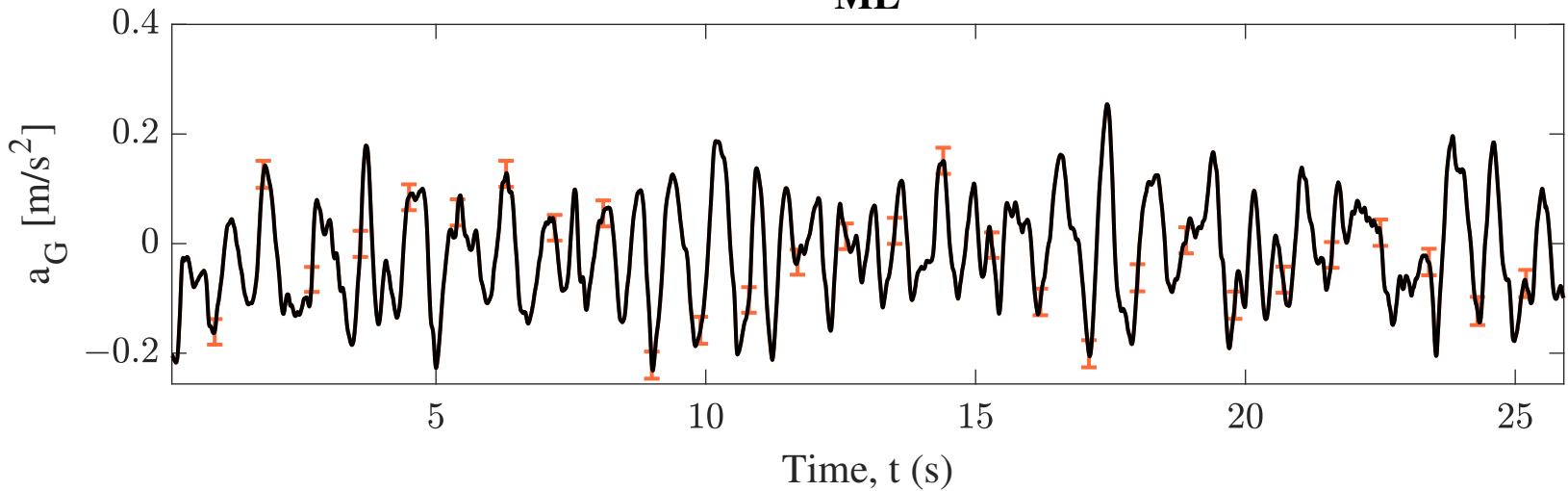**AP**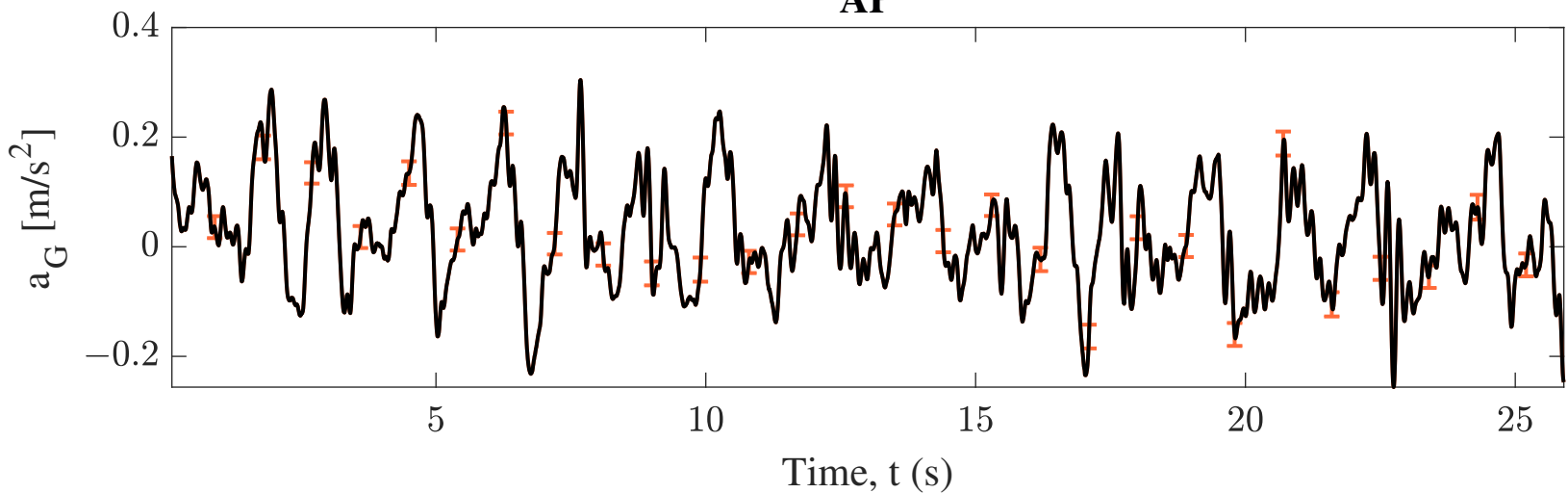**IS**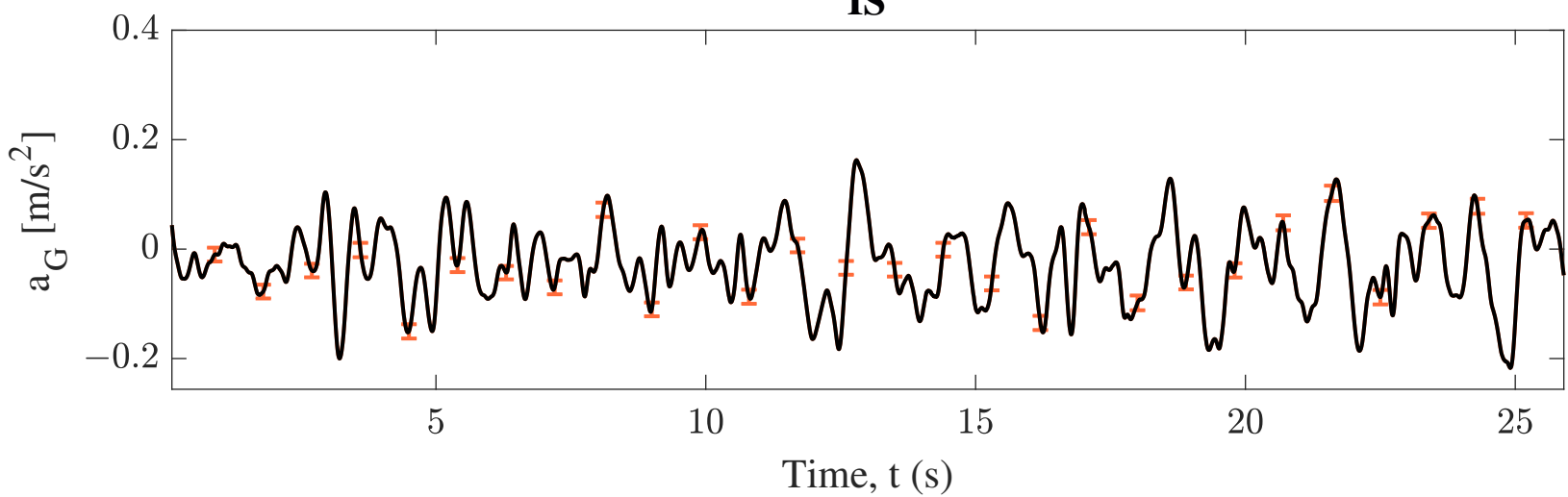

— FP — HH

Supplement: Supplementary file 1 [file sensors-25-02639-s001.zip › Validation of Mobile Devices w Force Plates for Balance Assessment/figures/Results/Trial4_Fig3.pdf]

**ML**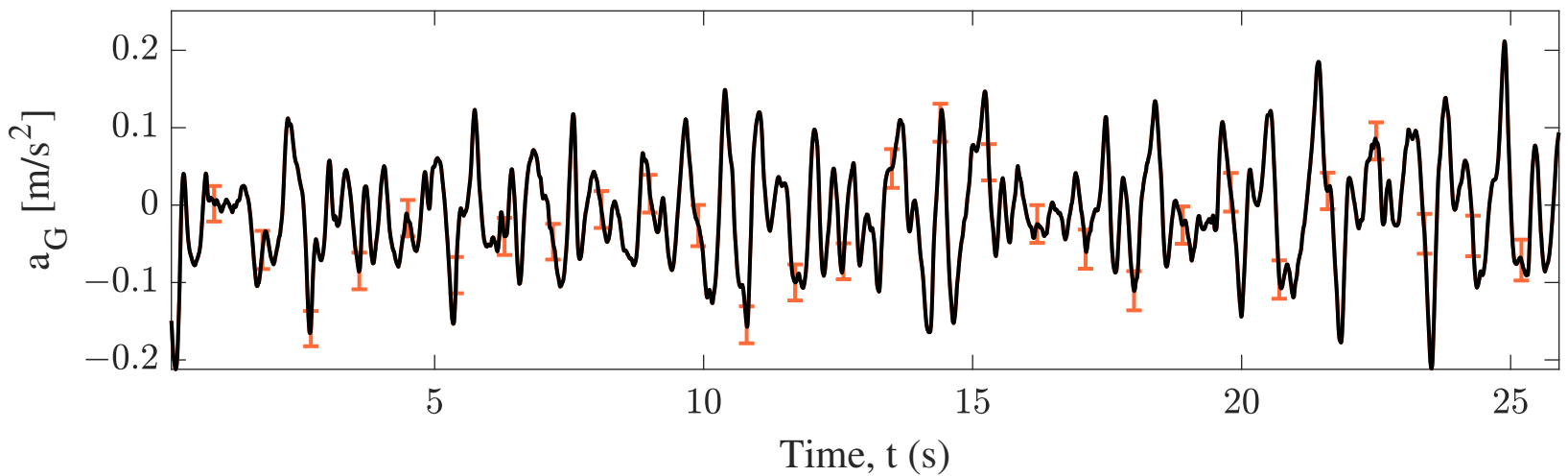**AP**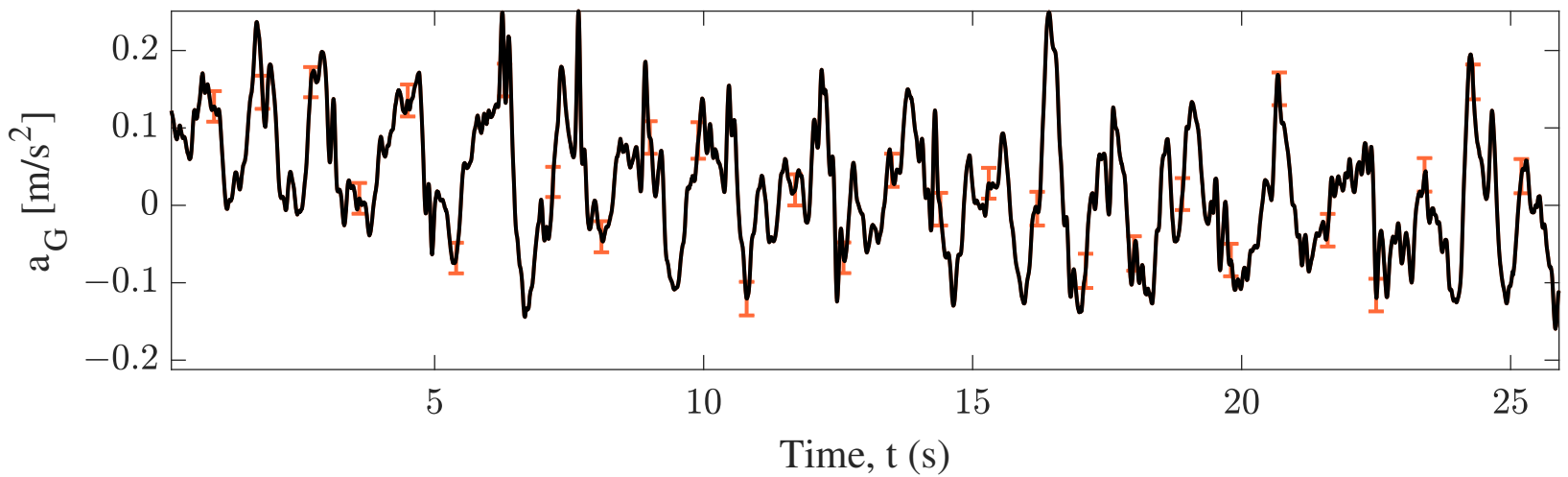**IS**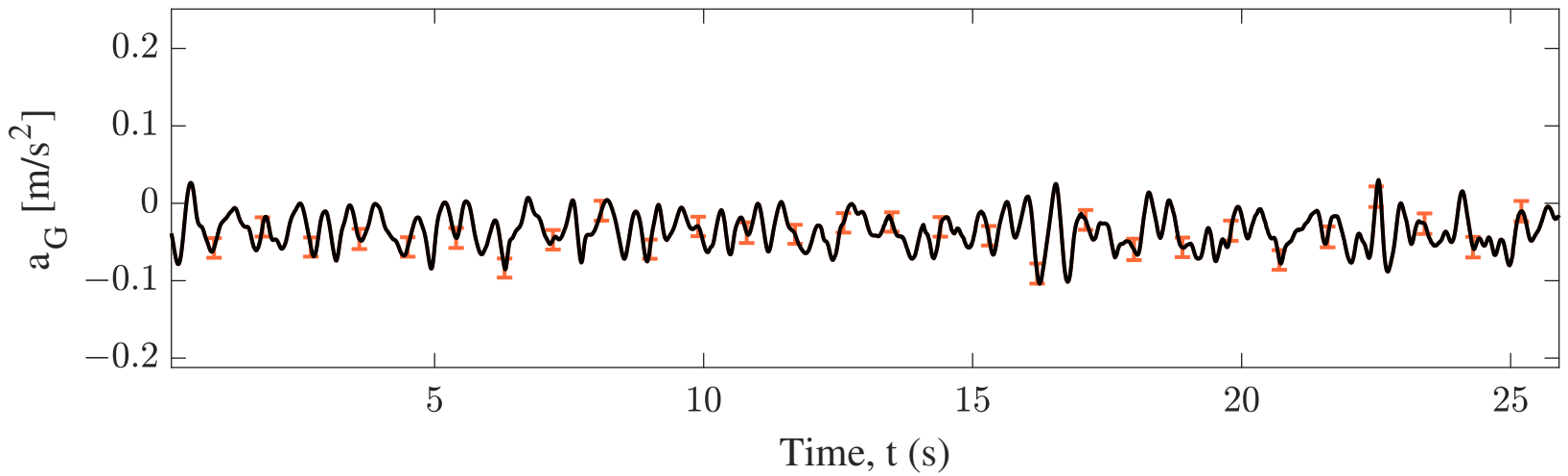

— FP - BH

Supplement: Supplementary file 1 [file sensors-25-02639-s001.zip › Validation of Mobile Devices w Force Plates for Balance Assessment/figures/Results/Trial4_Fig4.pdf]

**ML**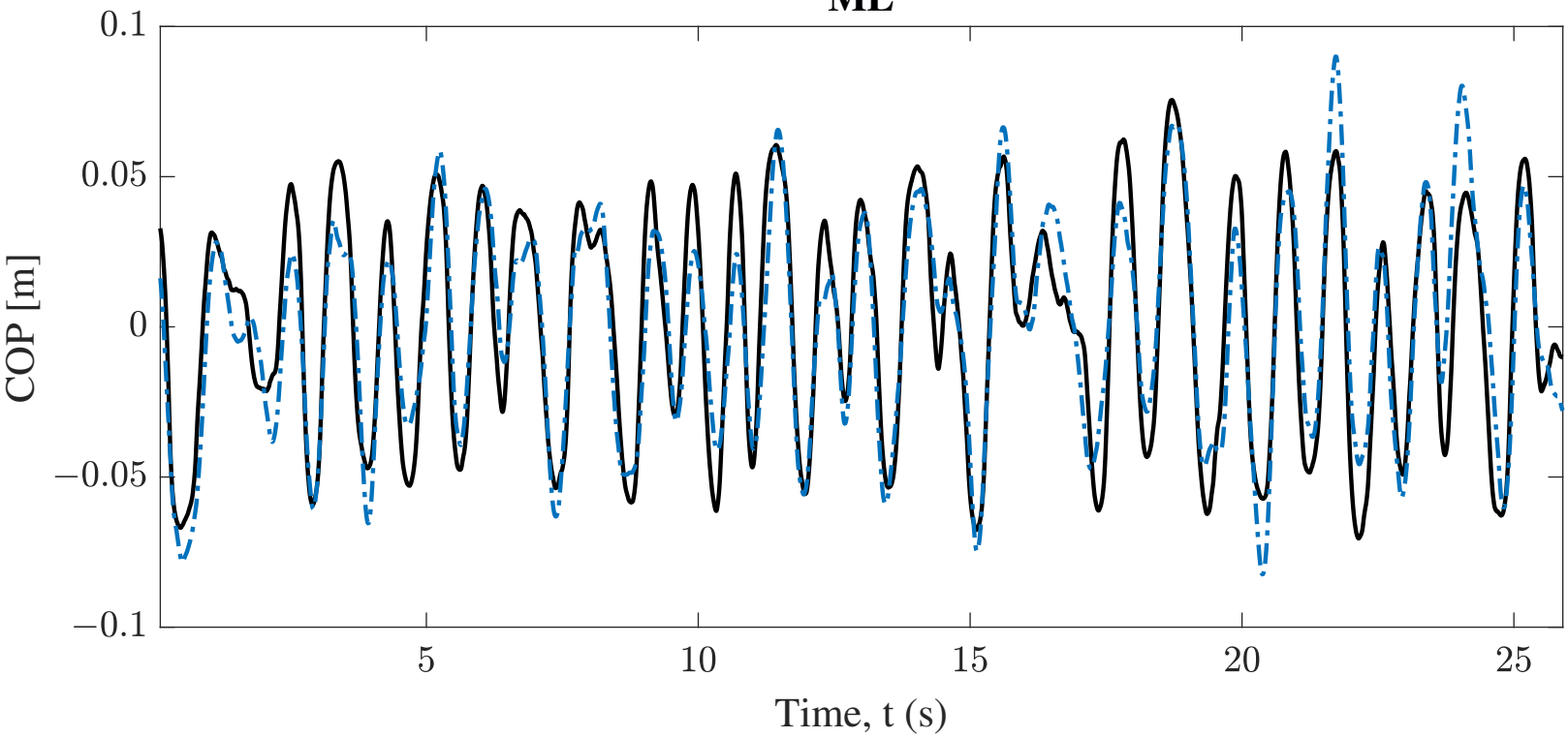**AP**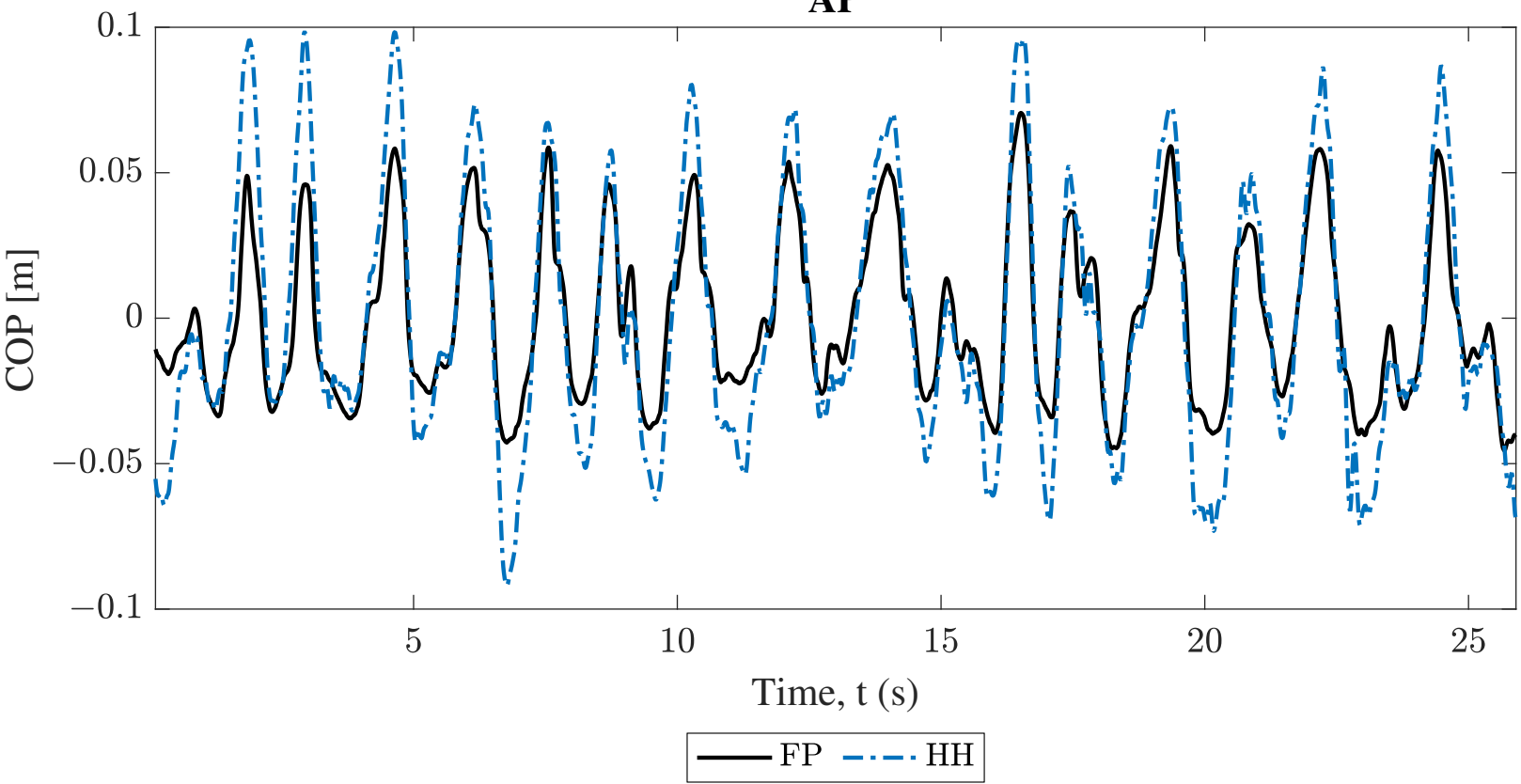

Supplement: Supplementary file 1 [file sensors-25-02639-s001.zip › Validation of Mobile Devices w Force Plates for Balance Assessment/figures/Results/Trial4_Fig5.pdf]

**ML**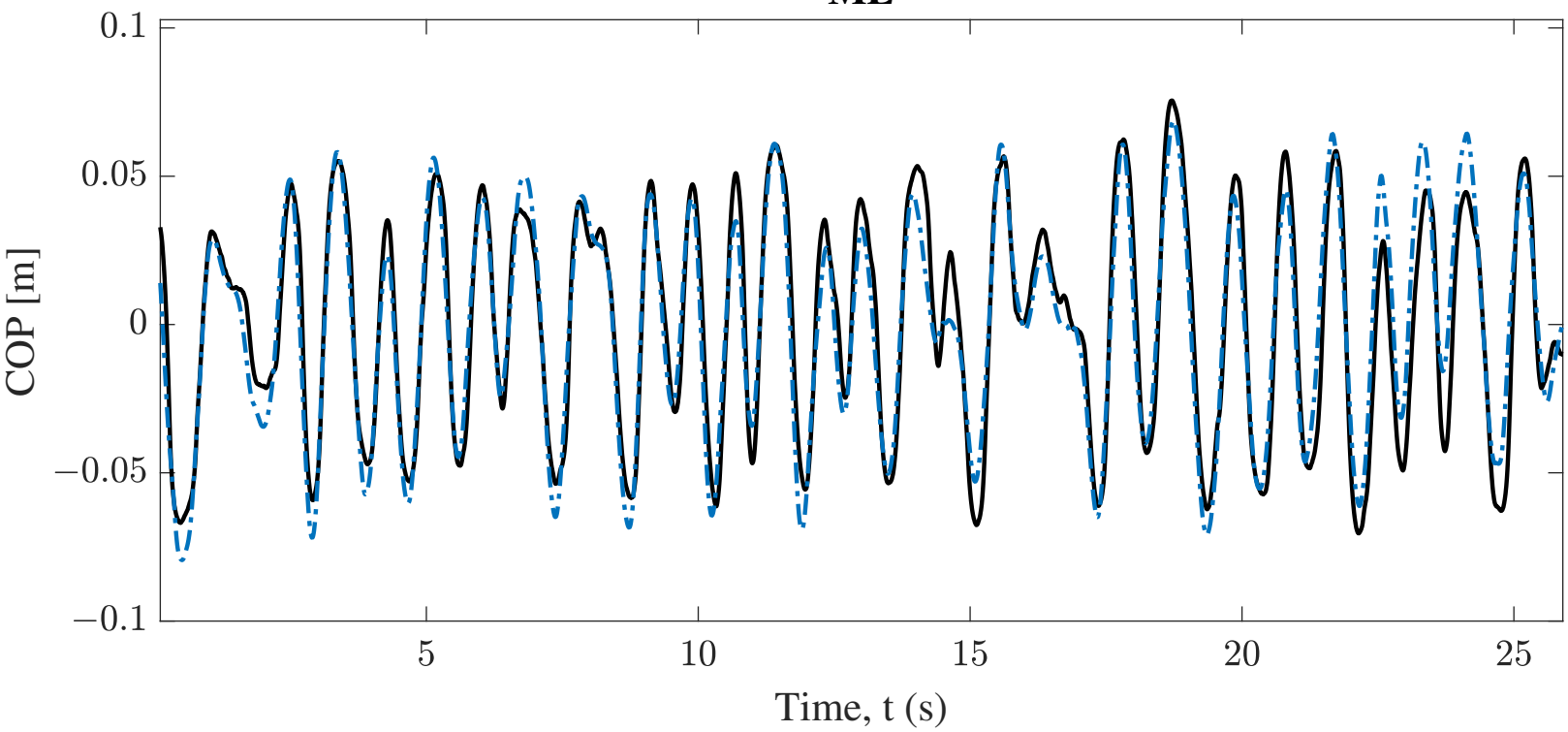**AP**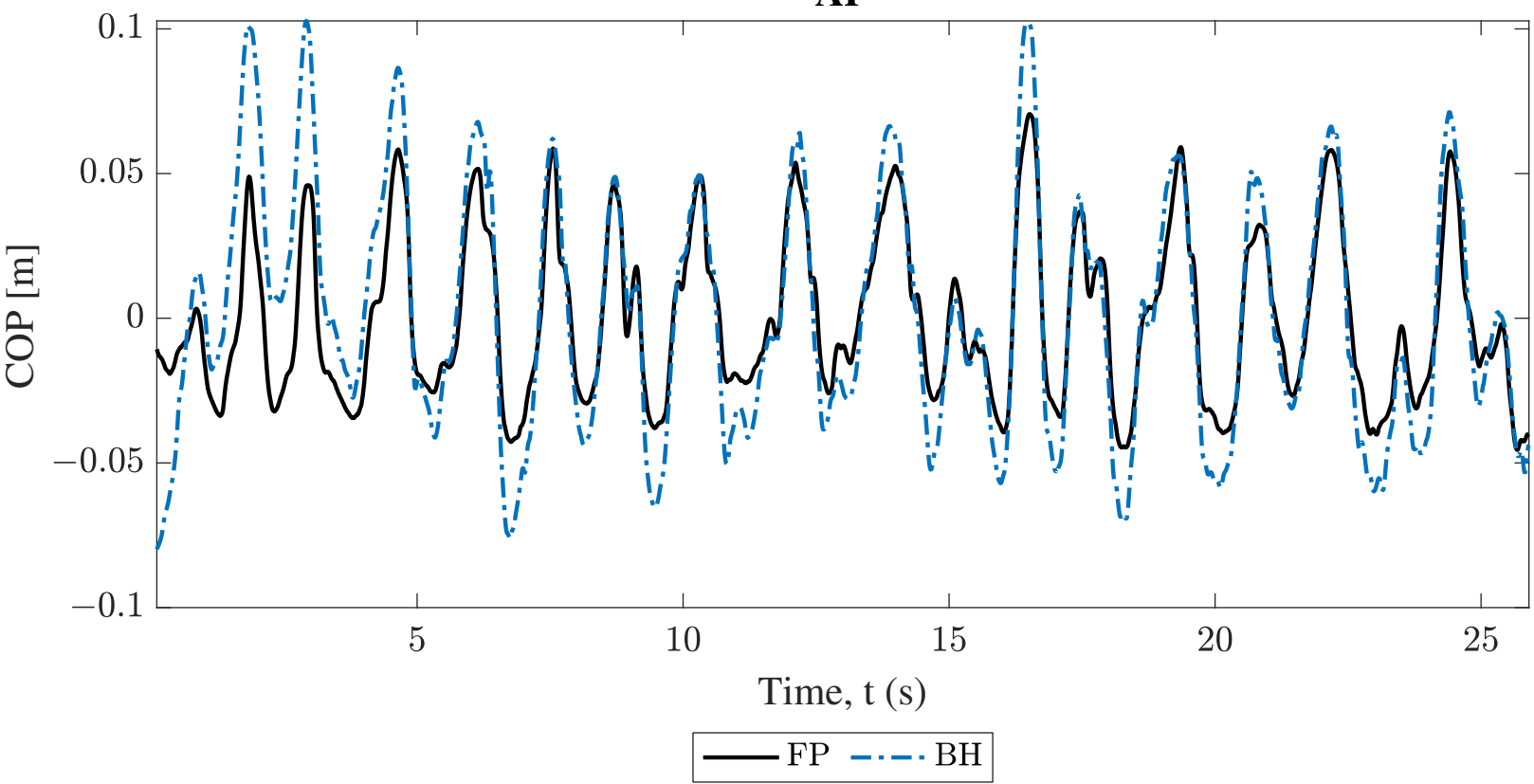

Supplement: Supplementary file 1 [file sensors-25-02639-s001.zip › Validation of Mobile Devices w Force Plates for Balance Assessment/figures/Results/Trial4_Fig6.pdf]

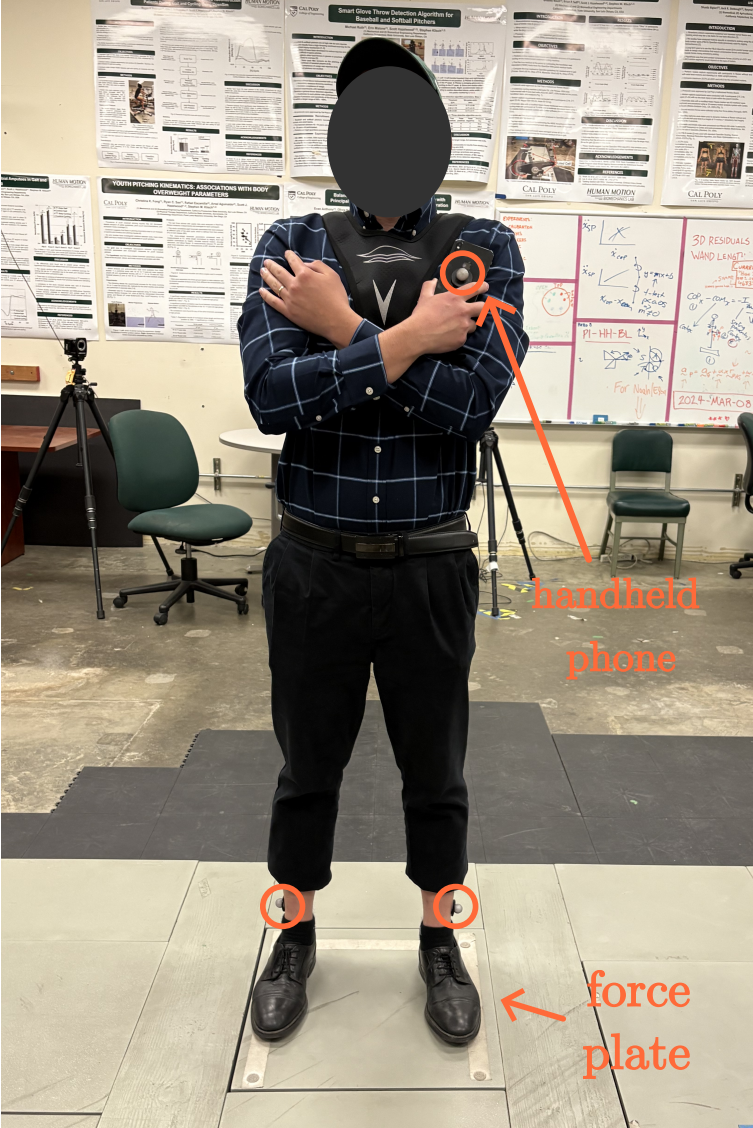

(a)

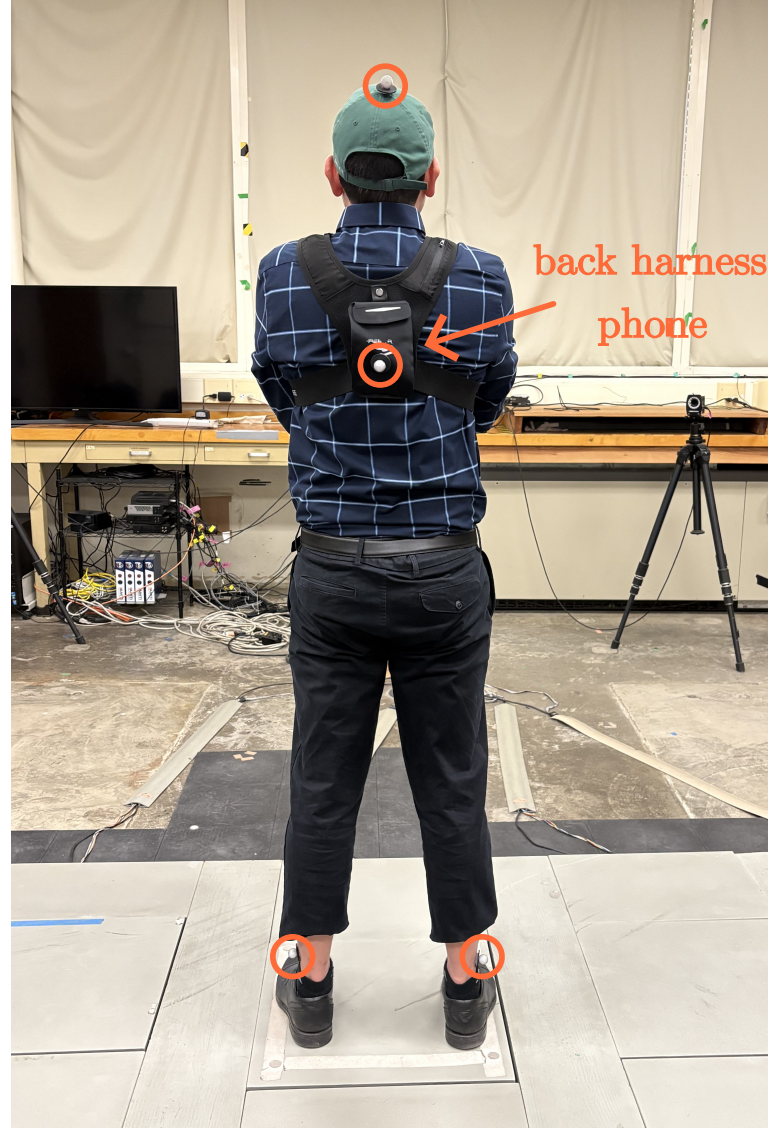

(b)

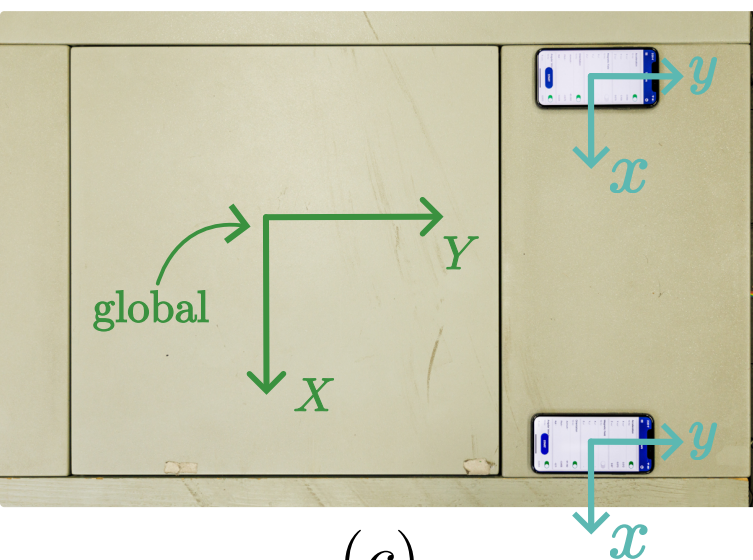

(c)

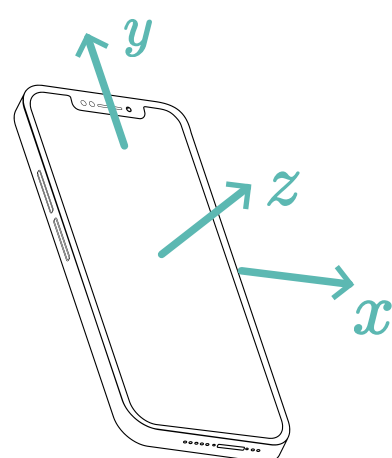

(d)

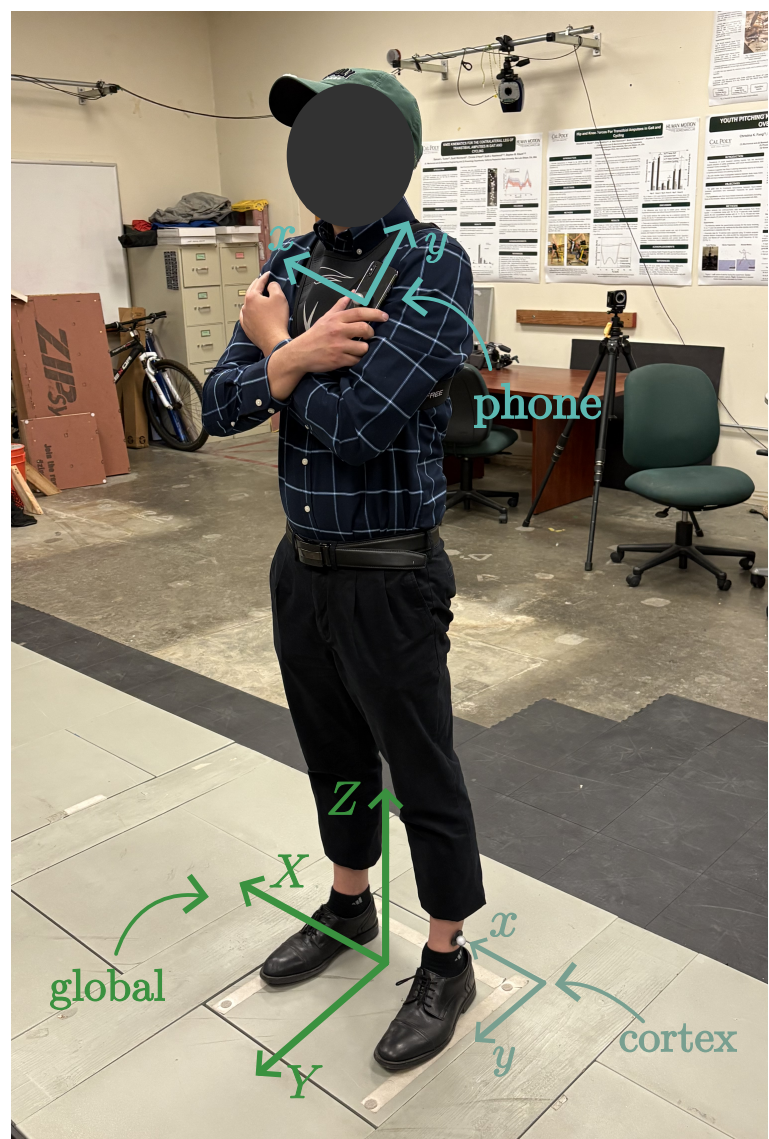

(e)

Supplement: Supplementary file 1 [file sensors-25-02639-s001.zip › Validation of Mobile Devices w Force Plates for Balance Assessment/figures/Setup_Updated.pdf]
